# Co-operative and Hierarchical Binding of c-FLIP and Caspase-8: A Unified Model Defines How c-FLIP Isoforms Differentially Control Cell Fate

**DOI:** 10.1016/j.molcel.2016.02.023

**Published:** 2016-03-17

**Authors:** Michelle A. Hughes, Ian R. Powley, Rebekah Jukes-Jones, Sebastian Horn, Maria Feoktistova, Louise Fairall, John W.R. Schwabe, Martin Leverkus, Kelvin Cain, Marion MacFarlane

**Affiliations:** 1 MRC Toxicology Unit, Hodgkin Building, P.O. Box 138, Lancaster Road, Leicester LE1 9HN, UK; 2 Henry Wellcome Laboratories of Structural Biology, Department of Molecular and Cell Biology, University of Leicester, Lancaster Road, Leicester LE1 9HN, UK; 3 Department of Dermatology, Venereology and Allergology, Medical Faculty Mannheim, University of Heidelberg, Theodor-Kutzer-Ufer 1-3, 68167 Mannheim, Germany; 4 Department of Dermatology and Allergology, Medical Faculty of the RWTH Aachen, Pauwelsstraße 30, 52074 Aachen, Germany

## Abstract

The death-inducing signaling complex (DISC) initiates death receptor-induced apoptosis. DISC assembly and activation are controlled by c-FLIP isoforms, which function as pro-apoptotic (c-FLIP_L_ only) or anti-apoptotic (c-FLIP_L_/c-FLIP_S_) regulators of procaspase-8 activation. Current models assume that c-FLIP directly competes with procaspase-8 for recruitment to FADD. Using a functional reconstituted DISC, structure-guided mutagenesis, and quantitative LC-MS/MS, we show that c-FLIP_L/S_ binding to the DISC is instead a co-operative procaspase-8-dependent process. FADD initially recruits procaspase-8, which in turn recruits and heterodimerizes with c-FLIP_L/S_ via a hierarchical binding mechanism. Procaspase-8 activation is regulated by the ratio of unbound c-FLIP_L/S_ to procaspase-8, which determines composition of the procaspase-8:c-FLIP_L/S_ heterodimer. Thus, procaspase-8:c-FLIP_L_ exhibits localized enzymatic activity and is preferentially an activator, promoting DED-mediated procaspase-8 oligomer assembly, whereas procaspase-8:c-FLIP_S_ lacks activity and potently blocks procaspase-8 activation. This co-operative hierarchical binding model explains the dual role of c-FLIP_L_ and crucially defines how c-FLIP isoforms differentially control cell fate.

## Highlights

• c-FLIP isoforms (L/S) do not directly compete with caspase-8 for binding to FADD
• c-FLIP binds to the DISC via a co-operative hierarchical caspase-8-dependent process
• Co-operative and hierarchical binding crucially explains the dual function of c-FLIP_L_
• Our unified model defines how c-FLIP isoforms differentially direct cell fate

## Graphical Abstract

**Figure:**
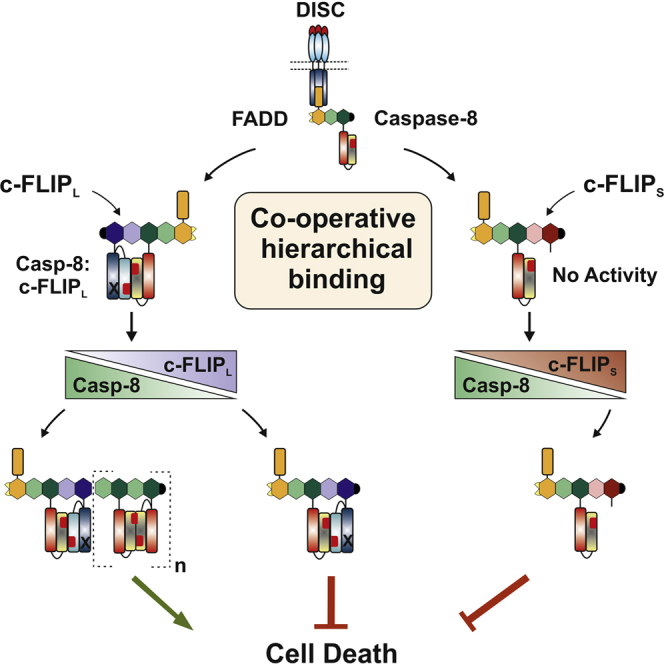

## Teaser

It is unclear how c-FLIP isoforms can differentially regulate caspase-8 activation to direct cell fate. Hughes et al. show that c-FLIP_L/S_ recruitment to FADD is indirect and requires caspase-8. This co-operative hierarchical binding process explains the conundrum of the dual role of c-FLIP_L_ and defines how c-FLIP_L/S_ differentially control cell fate.

## Introduction

Apoptotic cell death, which plays a fundamental role during development and homeostasis of multicellular organisms, is orchestrated by the caspase family of cysteine proteases. Deregulated apoptosis is a hallmark of several diseases, including autoimmunity, neurodegeneration, and cancer. The extrinsic apoptotic pathway is initiated by “death ligand”-induced ligation of death receptors (DR), such as CD95 (Fas/Apo1), TRAIL (TNF-related apoptosis-inducing ligand) receptors-1/-2, (TRAIL-R1/R2) and tumor necrosis factor (TNF) receptor-1 (TNF-R1), which form part of the TNFR superfamily (Dickens et al., 2012b). Stimulation of CD95 or TRAIL-R1/R2 by their cognate ligands or agonistic antibodies triggers formation of a multiprotein death-inducing signaling complex (DISC), comprising receptors, the bipartite adaptor molecule FADD (Fas-associated death domain protein), the initiator caspases-8 and -10, and the catalytically inactive caspase-8 homolog, c-FLIP (Kischkel et al., 1995). FADD is recruited to DR through direct interactions of the death domains (DD) present on both proteins; this exposes the FADD death effector domain (DED) (Esposito et al., 2010, Scott et al., 2009, Wang et al., 2010), promoting recruitment of DED-only proteins including procaspase-8. Once recruited to FADD, multiple procaspase-8 molecules interact via their tandem DEDs forming a DED chain-based procaspase-8 activation platform (Dickens et al., 2012a, Schleich et al., 2012), thereby facilitating both proximity-induced dimerization and proteolytic cleavage of procaspase-8, which are required for initiation of apoptotic cell death (Hughes et al., 2009, Oberst et al., 2010).

In addition to its key role in apoptosis, caspase-8 has a survival role as it is required for embryonic development (Dillon et al., 2012, Varfolomeev et al., 1998), immune cell proliferation (Salmena et al., 2003), and resistance to RIPK1-RIPK3-mediated programmed necrosis (Kaiser et al., 2011, Oberst et al., 2011). In all of these roles, c-FLIP is a key regulator that determines the activity of caspase-8 (Dillon et al., 2012, Hinshaw-Makepeace et al., 2008, Koenig et al., 2014, Oberst et al., 2011). Although c-FLIP has multiple splice forms at the mRNA level, two major protein isoforms predominate, namely c-FLIP long (c-FLIP_L_) and c-FLIP short (c-FLIP_S_) (Irmler et al., 1997, Scaffidi et al., 1999). c-FLIP_S_ is a truncated version of procaspase-8 containing tandem DEDs only, whereas c-FLIP_L_ closely resembles full-length procaspase-8 but critically lacks the active site catalytic cysteine residue and proteolytic activity. c-FLIP_S_ inhibits DR-mediated apoptosis by blocking caspase-8 activation at the DISC (Krueger et al., 2001b, Scaffidi et al., 1999). While c-FLIP_S_ appears to act purely as an antagonist of caspase-8 activity, c-FLIP_L_ has a more controversial role, being variously reported as either an activator or inhibitor of procaspase-8 (reviewed in Thome and Tschopp, 2001, Oztürk et al., 2012). Hence, during both development and immune cell proliferation, c-FLIP_L_:procaspase-8 heterodimers function to inhibit RIPK1-RIPK3-mediated programmed necrosis (Oberst et al., 2011). Likewise, the Ripoptosome, which is formed upon genotoxic stress or loss of inhibitor-of-apoptosis proteins (IAPs), is regulated by c-FLIP_L/S_:procaspase-8 heterodimers (Feoktistova et al., 2011, Feoktistova et al., 2012, Tenev et al., 2011). Thus, in a variety of signaling complexes, regulation of caspase-8 by c-FLIP isoforms is a critical step in determining signaling outcome resulting in cell survival or diverse modes of cell death.

The key question is how does c-FLIP modulate procaspase-8 activation/activity to produce diverse signaling outcomes? Current models propose that c-FLIP competes directly with procaspase-8 for binding to FADD through homotypic DED interactions, thus inhibiting procaspase-8 recruitment and activation at the DISC (Irmler et al., 1997, Rasper et al., 1998, Yang et al., 2005). Thus, both c-FLIP_S_ and overexpressed c-FLIP_L_ can block DISC-dependent procaspase-8 activation (Fricker et al., 2010, Kavuri et al., 2011, Krueger et al., 2001b, Scaffidi et al., 1999). However, lower physiological levels of c-FLIP_L_ enhance procaspase-8 activity as c-FLIP_L_ forms a highly active heterodimeric complex with procaspase-8 (Boatright et al., 2004), resulting in increased apoptosis (Chang et al., 2002, Fricker et al., 2010, Micheau et al., 2002, Scaffidi et al., 1999). Although difficult to explore biochemically, studies using truncated c-FLIP_L_ and caspase-8 proteins lacking their DED-containing prodomains have revealed that heterodimerization of c-FLIP_L_ with procaspase-8 leads to rearrangement of the catalytic site of procaspase-8, producing an active conformation (Chang et al., 2002, Micheau et al., 2002, Yu et al., 2009). Similarly, in vitro and structural studies show that caspase-8 has a higher affinity for c-FLIP_L_ than for itself, suggesting that procaspase-8:c-FLIP_L_ heterodimers may be preferred over procaspase-8 homodimers. Furthermore, c-FLIP_L_ can heterodimerize with and activate non-cleavable procaspase-8 without autoprocessing, resulting in a limited/selective substrate specificity (Boatright et al., 2004, Pop et al., 2011, Yu et al., 2009). Thus, it is possible that procaspase-8:c-FLIP_L_ heterodimers can potentially initiate different signaling outcomes. However, the mechanism of c-FLIP_L/S_ recruitment into these FADD-containing signaling complexes and how this controls procaspase-8 recruitment/activation to effect and direct alternate signaling outcomes has not been delineated.

We now reveal a hitherto unknown mechanism that explains how c-FLIP_L/S_ are recruited to the DISC and differentially regulate caspase-8 activation to control cell fate. An in vitro DISC reconstitution model utilizing structure-guided DED mutants of full-length FADD, procaspase-8, and c-FLIP_L/S_ has revealed that, contrary to current thinking, c-FLIP isoforms do not directly compete with procaspase-8 for binding to FADD. Surprisingly, optimal c-FLIP recruitment to the DISC is a co-operative and hierarchical process in which procaspase-8 binding to FADD is the primary initiating event, which in turn promotes recruitment of c-FLIP via heterodimerization with procaspase-8. Thus, we have identified a regulatory mechanism involving procaspase-8:c-FLIP_L/S_ heterodimers where procaspase-8 activation is determined by composition of the heterodimer. Consequently, procaspase-8:c-FLIP_L_, which exhibits local heterodimer-dependent enzymatic activity, functions primarily as a DISC activator by promoting DED-mediated recruitment of multiple procaspase-8 molecules. In contrast, procaspase-8:c-FLIP_S_ lacks enzymatic activity and potently blocks procaspase-8 activation. Moreover, using quantitative mass spectrometry and confocal imaging, we show that high levels of c-FLIP_S_ (or c-FLIP_L_) inhibit DISC activation by blocking formation of DED-mediated procaspase-8 oligomers. Significantly, this alternative mechanism for c-FLIP_L/S_ recruitment to the DISC and regulation of DED:DED interactions critically determines procaspase-8 function. We now propose a co-operative and hierarchical binding model that explains the conundrum of the dual functionality of c-FLIP_L_ and crucially defines how c-FLIP isoforms differentially control cell fate. This unified model represents a paradigm shift in terms of our understanding of how c-FLIP regulates the DISC as well as other caspase-8-dependent signaling platforms involved in development, physiology, and disease.

## Results

### Procaspase-8 Dimerization, but Not Cleavage, Is Essential for DISC-Associated c-FLIP_L_ Processing

**Figure 1:**
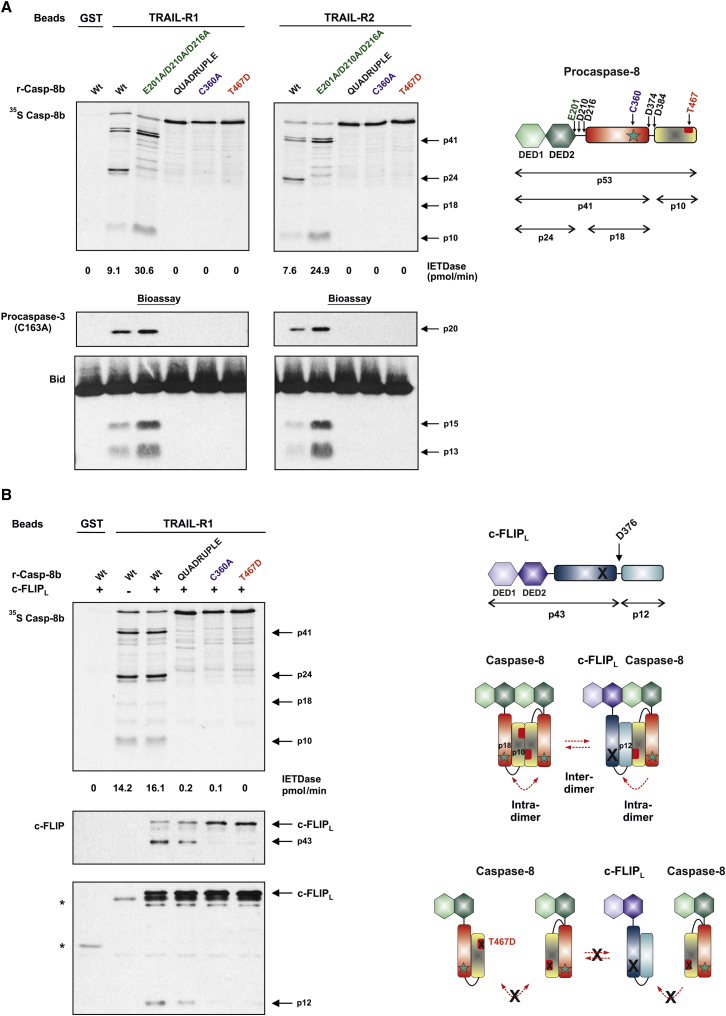
Procaspase-8 Dimerization Is Required for Procaspase-8 Cleavage of c-FLIP_L_ at the DISC (A) Domain structure of procaspase-8b showing cleavage sites, active site (C360), and dimerization residue (T467) (Machα1 numbering). Reconstituted TRAIL DISC was assembled using GST-TRAIL-R1/R2 intracellular domain (TRAIL-R1/R2-IcD), recombinant FADD (r-FADD), and ^35^S-labeled recombinant procaspase-8b (^35^S r-Casp-8b, 100 μl) wild-type (WT), highly active (E201A/D210A/D216A), non-cleavable Quadruple (D210A/D216A/D374A/D384A), active site (C360A), or dimerization (T467D) mutants. TRAIL-R1/R2 DISCs were analyzed for r-Casp-8b cleavage fragments, IETDase activity, and proteolytic cleavage of procaspase-3 (C163A) or Bid. See also Figure S1. (B) TRAIL-R1 DISC (r-DISC) assembled using indicated ^35^S r-Casp-8b variants (100 μl) alone (WT only) or in combination with c-FLIP_L_ (25 μl). Beads were assessed for r-Casp-8b cleavage, c-FLIP_L_ cleavage, and IETDase activity. Domain structure of c-FLIP_L_ showing caspase-8 cleavage site (D376). Scheme shows intra-dimer/inter-dimer cleavage of c-FLIP_L_ when combined with r-Casp-8b WT or dimerization mutant (T467D). ^∗^non-specific band.

In this study, we have reconstituted a fully functional TRAIL DISC using only GST-tagged TRAIL-R1/R2 intracellular domain (TRAIL-R1/R2-IcD) bound to glutathione beads, full-length recombinant FADD (r-FADD), and procaspase-8b (procaspase-8). In this model system, procaspase-8 cleavage to its signature fragments and IETDase activity were only detected in the presence of TRAIL-R1/R2-IcD, r-FADD, and ^35^S-labeled procaspase-8 (Figures 1A and S1A). Significantly, introducing an *lpr*-like mutation into TRAIL-R1/R2-IcD, which inhibits CD95 DD-mediated FADD recruitment and receptor signaling, or DISC reconstitution with TRAIL-R4-IcD, which has a truncated intracellular domain and cannot signal for apoptosis, abolished FADD recruitment, procaspase-8 binding, and IETDase activity (Figure S1B). These results demonstrate that the reconstituted TRAIL-R1/R2 DISC fully recapitulates the functional assembly and proteolytic activity of a native DISC.

We then investigated the mechanism of procaspase-8 activation in the TRAIL-R1/R2 DISC using a cleavage bioassay for exogenous DISC substrates essential for receptor-mediated apoptosis, namely procaspase-3 and Bid. Both procaspase-3 (C163A) and Bid were efficiently cleaved by a DISC reconstituted with wild-type (WT) procaspase-8 (Figure 1A). Moreover, the E201A/D210A/D216A procaspase-8 mutant, which only undergoes initial autocatalytic cleavage at D374/D384 generating a highly active p41/p10 form of caspase-8, further enhanced DISC-associated IETDase activity and procaspase-3/Bid cleavage. In marked contrast, although non-cleavable (Quadruple), active-site (C360A), or dimerization (T467D) mutants of procaspase-8 were efficiently recruited to the TRAIL-R1/R2 DISC, they did not support IETDase activity or cleavage of procaspase-3/Bid (Figure 1A). Thus, both dimerization and cleavage of procaspase-8 are absolutely required to achieve a fully functional TRAIL-R1/R2 DISC, further supporting the two-step procaspase-8 activation mechanism initially proposed for the CD95 DISC (Hughes et al., 2009).

As well as being a regulator of DISC function, c-FLIP_L_ is also a substrate for active caspase-8, which cleaves c-FLIP_L_ at D376, generating a DED containing p43 fragment and a C-terminal p12 fragment (Figure 1B) (Krueger et al., 2001b, Micheau et al., 2002, Scaffidi et al., 1999, Shu et al., 1997). To elucidate the mechanism of procaspase-8-mediated cleavage of c-FLIP_L_, we assessed whether various caspase-8 mutants could cleave c-FLIP_L_ within a reconstituted TRAIL-R1 DISC (r-DISC). c-FLIP_L_ was cleaved to its signature p43/p12 fragments by both activated WT and non-cleavable (Quadruple) procaspase-8, but not by an active site (C360A) or dimerization (T467D) mutant (Figure 1B), demonstrating that procaspase-8 dimerization is essential for processing adjacent c-FLIP_L_ molecules. Procaspase-8 can also form heterodimers with c-FLIP_L_, facilitating intra-dimer/inter-dimer -mediated c-FLIP_L_ cleavage (Chang et al., 2002). Thus, procaspase-8/c-FLIP_L_ heterodimerization and/or procaspase-8 homodimerization (Figure 1B, scheme), but not procaspase-8 cleavage, is crucial for c-FLIP_L_ processing within the DISC.

### c-FLIP_L_/Procaspase-8 Heterodimerization Is Essential for Concentration-Dependent c-FLIP_L_ Activation of Procaspase-8

**Figure 2:**
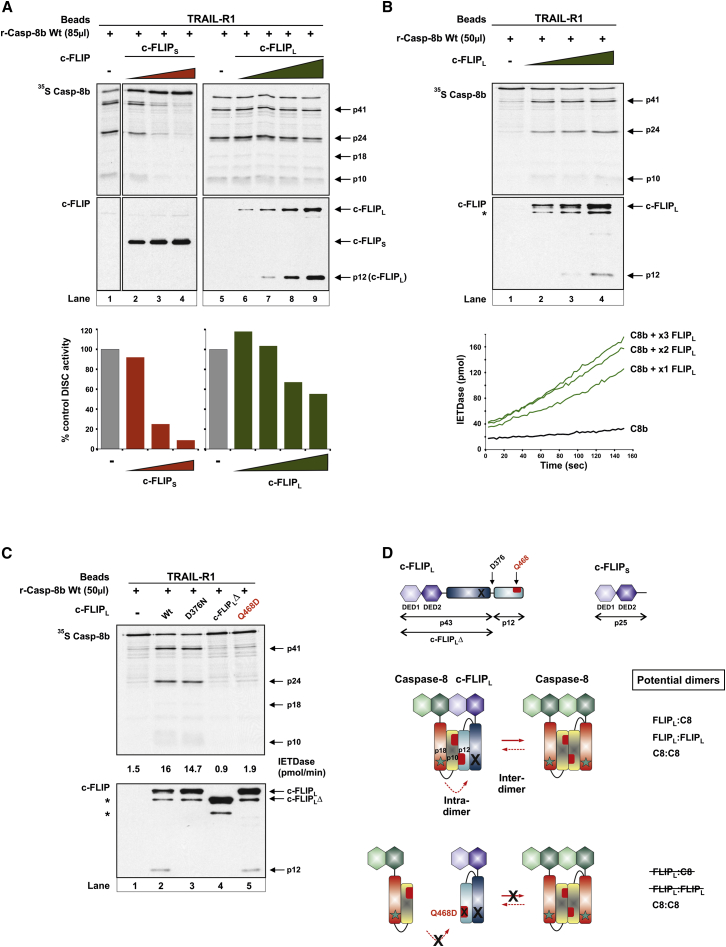
c-FLIP_L_/Procaspase-8 Heterodimerization Is Critical for c-FLIP-Mediated Procaspase-8 Activation (A) r-DISCs assembled with ^35^S r-Casp-8b (85 μl) alone (−) or with increasing amounts of c-FLIP_S_ (0–75 μl) or c-FLIP_L_ (0–225 μl). Beads were analyzed for r-Casp-8b cleavage, c-FLIP_L/S_, and IETDase activity; control (−) r-DISC activity expressed as 100%. (B) r-DISC reconstituted (16 hr at 16°C) using sub-optimal levels of ^35^S r-Casp-8b (50 μl) with increasing amounts of c-FLIP_L_ (0–75 μl). Beads were analyzed as in (A); graph shows time-dependent IETD.AFC hydrolysis. (C) r-DISCs assembled (16 hr at 16°C) with ^35^S r-Casp-8b (50 μl) alone (−) or with c-FLIP_L_ mutants (50 μl) shown in (D). Beads were analyzed for r-Casp-8b cleavage, c-FLIP_L_, and IETDase activity. (D) Domain organization of c-FLIP_L/S_ showing c-FLIP_L_ caspase-8 cleavage site (D376), dimerization residue (Q468), and p43 fragment (FLIP_L_ Δ). Scheme shows intra-dimer/inter-dimer activation of r-Casp-8b WT when combined with c-FLIP_L_ WT or dimerization mutant (Q468D). ^∗^non-specific band.

c-FLIP is a key regulator of procaspase-8 in several cell death signaling platforms including the DISC (Fricker et al., 2010, Krueger et al., 2001a, Oztürk et al., 2012), the necrosome (Dillon et al., 2012, Oberst et al., 2011, Vanlangenakker et al., 2012), and ripoptosome (Feoktistova et al., 2011, Feoktistova et al., 2012, Tenev et al., 2011). At high levels of ectopic expression, c-FLIP_L_ and c-FLIP_S_ function as anti-apoptotic regulators (Irmler et al., 1997, Kavuri et al., 2011, Krueger et al., 2001a, Scaffidi et al., 1999). However, c-FLIP_L_ can also function as a critical activator of procaspase-8, amplifying DISC-mediated apoptosis (Chang et al., 2002, Micheau et al., 2002, Shu et al., 1997). To solve this apparent conundrum and to investigate how c-FLIP isoforms can differentially regulate procaspase-8, we investigated how c-FLIP_L_ or c-FLIP_S_ modulate procaspase-8 activation in the r-DISC (Figure 2A).

First, we showed that r-DISC assembly with increasing amounts of c-FLIP_S_ (Figure 2A, lanes 1–4) resulted in concentration-dependent inhibition of procaspase-8 cleavage/IETDase activity; thus, c-FLIP_S_ functions exclusively as an inhibitor of procaspase-8 activation. In contrast, c-FLIP_L_ exhibited a biphasic effect on r-DISC-mediated activation of procaspase-8 (Figure 2A, lanes 5–9), with low concentrations of c-FLIP_L_ promoting procaspase-8 activation, whereas higher levels of c-FLIP_L_ resulted in concentration-dependent inhibition of procaspase-8 activity. Thus, the c-FLIP_L_:procaspase-8 ratio within the DISC critically determines procaspase-8 activation and signaling outcome. We further characterized the ability of low levels of c-FLIP_L_ to enhance DISC-mediated procaspase-8 activation and apoptosis by reconstituting the r-DISC using sub-optimal levels of procaspase-8. Sub-optimal amounts of procaspase-8 displayed negligible proteolytic cleavage or activity against IETD.AFC (Figure 2B, lane 1). However, co-incubation with increasing amounts of c-FLIP_L_ produced a concentration-dependent activation of procaspase-8, as shown by autocatalytic cleavage of procaspase-8, its substrate c-FLIP_L_, and increased r-DISC-associated IETDase activity (Figure 2B, lanes 2–4; lower graph). Thus, high levels of c-FLIP_L_ inhibit DISC-mediated procaspase-8 activation, whereas at physiological concentrations (Chang et al., 2002), c-FLIP_L_ activates DISC-bound procaspase-8.

To characterize how c-FLIP_L_ activates procaspase-8, we assessed the impact of various c-FLIP_L_ mutants on r-DISC-mediated procaspase-8 activation (Figure 2C). The non-cleavable c-FLIP_L_ mutant, D376N, fully activated procaspase-8, resulting in IETDase activity comparable to WT c-FLIP_L_ (Figure 2C, lanes 1–3). In contrast, a c-FLIP_L_ mutant lacking the C-terminal p12 domain (c-FLIP_L_Δ) did not activate procaspase-8 (Figure 2C, lane 4). In agreement with a key role for c-FLIP_L_ p12 domain, a mutant defective in the predicted heterodimer interface of c-FLIP_L_ (Q468D) (Micheau et al., 2002, Yu et al., 2009) completely abrogated c-FLIP_L_-mediated activation of procaspase-8 (Figure 2C, lane 5). Thus, c-FLIP_L_/procaspase-8 heterodimerization, without c-FLIP_L_ cleavage, is essential for c-FLIP_L_-mediated procaspase-8 activation within the DISC (Figure 2D, scheme).

### DISC Reconstitution Reveals Co-operative Recruitment of c-FLIP and Procaspase-8

**Figure 3:**
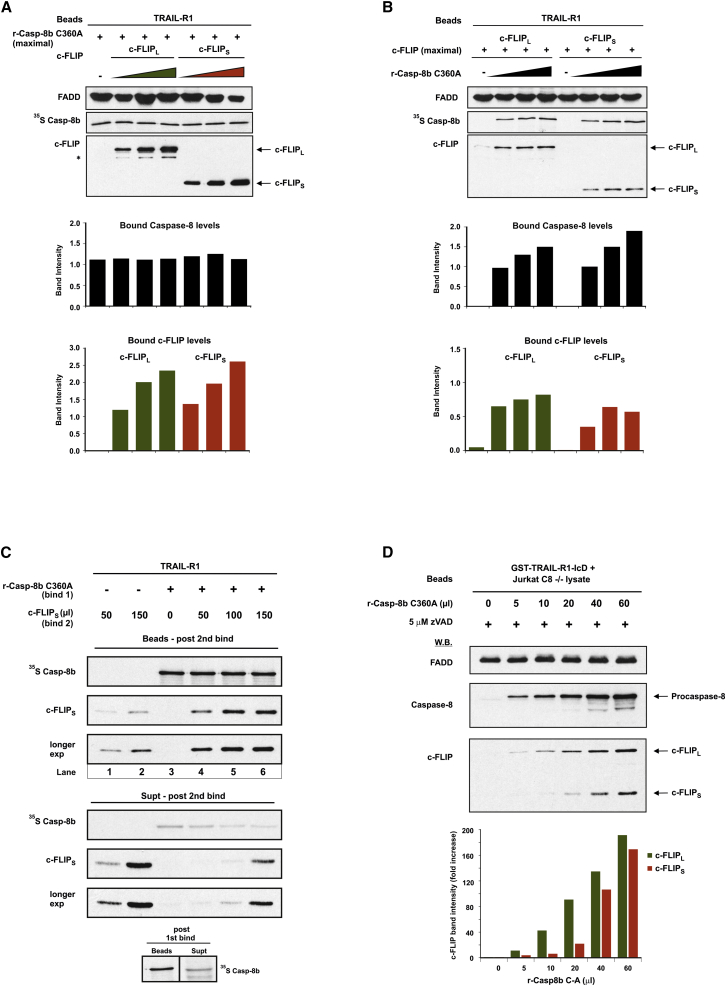
DISC Reconstitution Reveals Co-operative and Hierarchical Recruitment of c-FLIP_L/S_ and Procaspase-8 (A) r-DISCs assembled using ^35^S r-Casp-8b C360A (100 μl) with increasing amounts of c-FLIP_L/S_ (0–75 μl). Beads were analyzed for FADD, r-Casp-8b, and c-FLIP_L/S_ binding and quantified by densitometry (bar graphs). (B) r-DISCs reconstituted with c-FLIP_L/S_ (85 μl) and increasing amounts of ^35^S r-Casp-8b C360A (0–75 μl) were analyzed as in (A). (C) r-DISC was pre-assembled using TRAIL-R1-IcD, r-FADD, and ^35^S r-Casp-8b C360A (160 μl), and beads were washed to remove unbound protein before re-incubating with increasing amounts of c-FLIP_S_ (0–150 μl). Beads and supernatants, post first and second bind, were analyzed for r-Casp-8b and c-FLIP_S_. (D) TRAIL-R1-IcD pull downs from caspase-8 null Jurkat lysates combined with exogenous r-Casp-8b C360A (0–60 μl) were analyzed for FADD, procaspase-8, and c-FLIP_L/S_ binding and quantified by densitometry. ^∗^non-specific band. See also Figure S2.

Current models propose that c-FLIP inhibits apoptosis by competitively inhibiting procaspase-8 recruitment and activation within the DISC (Irmler et al., 1997, Rasper et al., 1998, Yang et al., 2005). We investigated this hypothesis further using the DISC reconstitution model to determine whether c-FLIP_L/S_ competes with procaspase-8 for binding to FADD. To assess binding of procaspase-8 and c-FLIP_L/S_, we reconstituted the r-DISC using excess levels of catalytically inactive (C360A) procaspase-8. Strikingly, co-incubation with increasing amounts of c-FLIP_L/S_ did not affect procaspase-8 recruitment to FADD. Indeed, despite the presence of procaspase-8, c-FLIP_L/S_ recruitment increased in a concentration-dependent manner (Figure 3A). Moreover, incubating excess amounts of c-FLIP_L/S_ with a pre-assembled r-DISC, containing maximal levels of procaspase-8 (C360A), failed to displace procaspase-8 from FADD, and both c-FLIP_L_ and c-FLIP_S_ were recruited to the pre-assembled complex (Figure S2A). Significantly, these data indicate that, contrary to current dogma, c-FLIP_L/S_ do not directly compete with procaspase-8 for binding to FADD. Instead, DISC recruitment of c-FLIP occurs via a mechanism that is different from procaspase-8.

To explore this further, the r-DISC was reconstituted with excess c-FLIP_L/S_ and increasing amounts of procaspase-8 (C360A). Remarkably, without procaspase-8, there was negligible recruitment of c-FLIP_L/S_ to FADD. However, increasing amounts of procaspase-8 resulted in concentration-dependent recruitment of both c-FLIP_L/S_ and procaspase-8 (Figure 3B), concomitant with decreasing amounts of unbound c-FLIP remaining in the supernatant (Figure S2B). Thus, r-DISC (TRAIL-R1 or CD95) recruitment of c-FLIP_L/S_ to FADD is significantly enhanced when procaspase-8 is present (Figures 3B and S2C). This conclusion was confirmed by adding increasing amounts of c-FLIP_S_ to a pre-assembled r-DISC, which, without procaspase-8 (C360A), recruited only minimal amounts of c-FLIP_S_ (Figure 3C). Conversely, when the r-DISC was pre-assembled with procaspase-8, c-FLIP_S_ recruitment was greatly enhanced (Figure 3C, compare lanes 1 and 2 with lanes 4–6). Intriguingly, it appears that only a finite amount of c-FLIP_S_ can be recruited to a fully pre-assembled r-DISC since addition of more c-FLIP_S_ did not further increase c-FLIP_S_ recruitment to the complex (Figure 3C, lanes 5 and 6). Crucially, our data reveal that c-FLIP does not displace procaspase-8 from FADD; instead, optimal c-FLIP recruitment to the DISC is critically dependent on procaspase-8.

To confirm that procaspase-8 is required for c-FLIP recruitment to the DISC, we extended our findings in a cellular context using caspase-8-deficient Jurkat T cells. TRAIL-R1 DISC formation was assessed in caspase-8-deficient cell lysates, supplemented with increasing amounts of recombinant procaspase-8 (C360A) (Figure 3D). Strikingly, despite efficient recruitment of FADD to TRAIL-R1-IcD, in the absence of procaspase-8 no endogenous c-FLIP_L/S_ was recruited to the DISC. However, addition of increasing amounts of exogenous procaspase-8 resulted in a concentration-dependent increase in recruitment of both c-FLIP_L_/c-FLIP_S_ to the DISC, concomitant with a decrease in c-FLIP_L/S_ in cell lysates (Figures 3D and S2D). Thus, c- FLIP_L/S_ do not compete with procaspase-8 for binding to FADD; instead, c-FLIP recruitment to the DISC requires procaspase-8. This provides strong evidence for an alternative model of DISC assembly involving co-operative recruitment of c-FLIP and procaspase-8.

### Mutation of Procaspase-8 DED1 Pocket Prevents Caspase-8 Recruitment to the DISC

**Figure 4:**
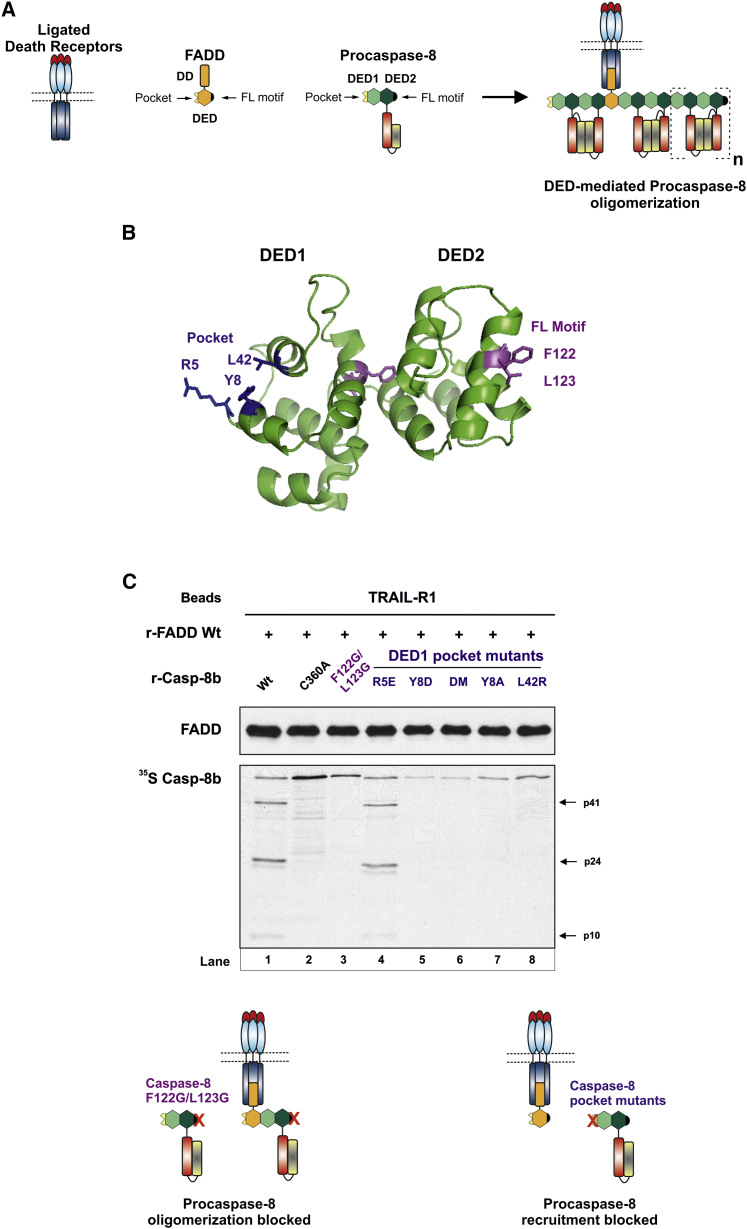
DISC-Bound FADD Recruits Procaspase-8 via Procaspase-8 DED1 Pocket (A) DISC model in which multiple procaspase-8 molecules are recruited to FADD via DED-mediated interactions. (B) Modeled structure of procaspase-8 DEDs showing residues in the DED1 pocket that potentially interact with the FL motif of FADD or DED2 of another procaspase-8 molecule. (C) r-DISC reconstituted using the indicated variants of ^35^S r-Casp-8b (100 μl) and beads assessed for FADD and r-Casp-8b binding (R5E/Y8D; DM). See also Figure S3. Scheme shows effect of mutating either procaspase-8 DED2 FL motif or key residues in the DED1 pocket.

To investigate the co-operative DISC recruitment of c-FLIP and procaspase-8, we first needed to characterize how procaspase-8 is recruited to FADD. A critical phenylalanine/leucine (F122/L123) hydrophobic motif in procaspase-8 DED2 has previously been shown to occupy a hydrophobic pocket in DED1 of an adjacent procaspase-8 molecule (Dickens et al., 2012a) and results in the formation of a DED chain-based procaspase-8 activation platform at the DISC (Figure 4A). However, mutation of procaspase-8 DED2 “FL motif” (F122G/L123G; double or single mutant) (Figure 4B) abrogates DED-mediated recruitment of additional procaspase-8 molecules to the DISC, but does not totally prevent procaspase-8 recruitment to FADD (Figure 4C, lanes 2 and 3; Figure S3), suggesting that recruitment to FADD is mediated via the procaspase-8 DED1 pocket. To explore this, we identified and mutated key residues in procaspase-8 DED1 pocket (Y8, R5, L42) that could be involved in interacting with an exposed FL motif on either FADD DED or procaspase-8 DED2 (Figure 4B). Mutation of the Y8 residue significantly reduced both DISC recruitment and activation of procaspase-8 when compared with WT or unprocessed active site mutant (C360A) (Figure 4C, compare lanes 5 and 7 with lanes 1 and 2). Strikingly, recruitment of procaspase-8 DED1 Y8D mutant was substantially less than that of DED2 FL motif mutant (Figure 4C, compare lanes 3 and 5). R5E pocket mutant alone had little effect on procaspase-8 recruitment or activation (Figure 4C, lane 4), while the R5E mutant in combination with Y8D (R5E/Y8D; DM) attenuated procaspase-8 recruitment similar to that observed with Y8D alone. An alternative pocket mutant, L42R, also exhibited reduced procaspase-8 recruitment, though not as great as that observed with the Y8 mutant (Figure 4C, lane 8). Thus, procaspase-8 recruitment to FADD occurs preferentially via procaspase-8 DED1 hydrophobic pocket, and Y8 is a key residue involved in this interaction (Figure 4C, scheme). Additional procaspase-8 molecules are then recruited via the exposed DED2 FL motif of FADD-bound procaspase-8 interacting with the hydrophobic pocket in DED1 of the next procaspase-8 molecule.

### The Mechanism of c-FLIP Recruitment to FADD Is Distinct from that of Procaspase-8

**Figure 5:**
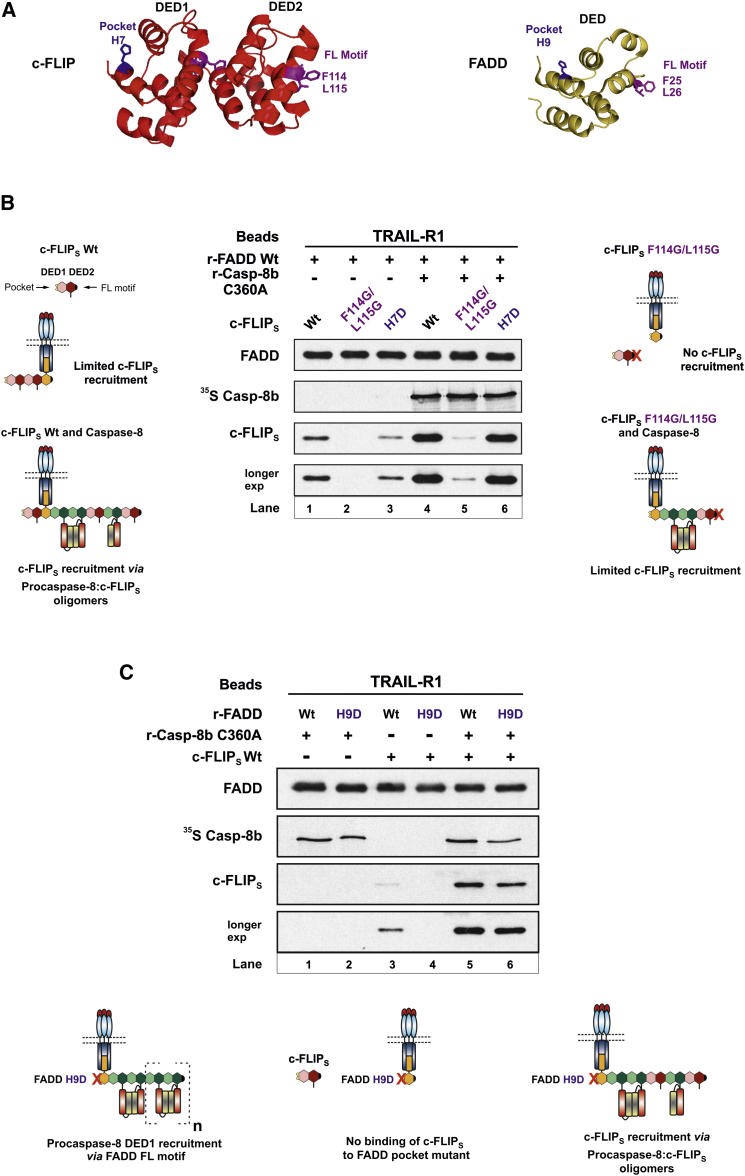
c-FLIP and Procaspase-8 Are Recruited to the DISC via Molecularly Distinct Mechanisms (A) Modeled structure of c-FLIP and FADD showing DED pocket residues or FL motifs that could potentially interact with another DED molecule. (B) r-DISC assembled using wild-type (WT), DED2 FL (F114G/L115G), or DED1 pocket (H7D) mutants of c-FLIP_S_ (50 μl) minus/plus ^35^S r-Casp-8b C360A (100 μl). Beads were assessed for FADD, r-Casp-8b, and c-FLIP_S_ binding. Schemes depict c-FLIP recruitment to the DISC in the absence or presence of procaspase-8. (C) r-DISC reconstituted using WT or DED pocket mutant (H9D) of r-FADD (5 μg) with ^35^S r-Casp-8b C360A (100 μl) and WT c-FLIP_S_ (50 μl), singly or in combination, and analyzed as in (B). Scheme showing FADD DED pocket mutant can only bind c-FLIP_S_ via procaspase-8:c-FLIP_S_ DED interactions. See also Figure S4.

Having demonstrated that FADD recruits procaspase-8 via the procaspase-8 DED1 pocket, we next investigated whether c-FLIP is recruited to FADD by the same mechanism. Therefore, we mutated equivalent residues to procaspase-8 in c-FLIP_S_, namely DED1 pocket residue (H7) and DED2 FL motif (F114/L115) (Figures 5A and S4A), and determined their impact on c-FLIP_S_ recruitment to the r-DISC. Without procaspase-8, only low levels of WT c-FLIP_S_ were recruited to FADD (Figure 5B, lane 1). However, mutation of DED2 FL motif (F114G/L115G) abrogated this c-FLIP_S_ recruitment to the r-DISC, indicating that without procaspase-8, limited binding of c-FLIP to FADD occurs predominantly via c-FLIP DED2 FL motif (Figure 5B, lane 2). Mutation of the c-FLIP_S_ DED1 pocket (H7D) slightly reduced, but did not abolish, c-FLIP_S_ binding to FADD (Figure 5B, compare lanes 1 and 3). Thus, in the absence of procaspase-8, the limited c-FLIP recruitment to FADD is primarily mediated through c-FLIP DED2 FL motif. Consistent with our earlier observation of co-operative DISC recruitment of c-FLIP and procaspase-8 (Figure 3), procaspase-8 greatly enhanced WT c-FLIP_S_ binding to the r-DISC (Figure 5B, compare lanes 1 and 4). Notably, in the presence of procaspase-8, c-FLIP_S_ DED2 F114G/L115G mutant was poorly recruited to the r-DISC with markedly reduced levels compared to WT c-FLIP_S_ (Figure 5B, lanes 4 and 5). In the presence of procaspase-8, recruitment of low levels of c-FLIP_S_ F114G/L115G mutant most likely occurs via DED-mediated caspase-8:c-FLIP_S_ interaction, with mutation of c-FLIP_S_ DED2 FL motif preventing recruitment of additional DED-containing molecules (Figure 5B). Intriguingly, recruitment of c-FLIP_S_ DED1 pocket mutant (H7D) was not impaired in the presence of procaspase-8, with binding of c-FLIP_S_ DED1 pocket mutant comparable to that of WT c-FLIP_S_ (Figure 5B, compare lanes 4 and 6). Hence, c-FLIP_S_ DED1 pocket mutant slightly reduced c-FLIP_S_ recruitment in the absence of procaspase-8, but mutation of c-FLIP_S_ DED1 pocket did not prevent co-operative recruitment of c-FLIP_S_ and procaspase-8 to the DISC (Figure S4B). These data provide evidence for a model of c-FLIP recruitment to FADD, which is clearly distinct from that of procaspase-8. Thus, in the absence of caspase-8, limited c-FLIP binding occurs via the c-FLIP DED2 FL motif, whereas procaspase-8 binding to FADD occurs via the procaspase-8 DED1 hydrophobic pocket.

To confirm our model of c-FLIP and procaspase-8 recruitment to the DISC, we tested the effect of a FADD DED hydrophobic pocket mutant (Figure 5A) on r-DISC recruitment of procaspase-8 or c-FLIP_S_ (minus/plus procaspase-8). When compared with WT FADD, mutation of FADD hydrophobic pocket (H9D) had minimal impact on r-DISC recruitment of procaspase-8 (Figure 5C, lanes 1 and 2). In marked contrast, this FADD DED pocket mutant completely abolished r-DISC recruitment of c-FLIP_S_ (Figure 5C, lanes 3 and 4), confirming that without procaspase-8, limited c-FLIP_S_ binding to FADD is mediated via c-FLIP_S_ DED2 FL motif and the hydrophobic pocket of FADD. Strikingly, when c-FLIP_S_ recruitment to FADD H9D was assessed in the presence of procaspase-8, r-DISC recruitment of c-FLIP_S_ was restored (Figure 5C, compare lanes 4 and 6). Moreover, although c-FLIP_S_ binding to FADD H9D was slightly reduced in the presence of procaspase-8, this correlated with a small reduction in FADD H9D procaspase-8 recruitment (Figure 5C, lanes 5 and 6; Figure S4C). Importantly, similar findings were also obtained with c-FLIP_L_ (data not shown). Thus, mutation of the H9 residue in the FADD DED pocket blocks c-FLIP_L/S_ recruitment, but addition of procaspase-8 restores c-FLIP_L/S_ recruitment via procaspase-8:c-FLIP DED-mediated interactions (Figure 5C, scheme). Mutation of the FADD pocket only slightly reduced procaspase-8 recruitment, confirming that procaspase-8 binding is primarily mediated through the FL motif of FADD and the DED1 pocket of procaspase-8, whereas c-FLIP is recruited indirectly to the DISC via co-operative binding with procaspase-8 by way of DED-mediated procaspase-8:c-FLIP heterodimerization (Figure 5C, scheme; Figure S4B). Taken together, these data firmly establish a co-operative and hierarchical mechanism for c-FLIP recruitment to the DISC.

### c-FLIP_S_ Inhibits DED-Mediated Oligomerization of Procaspase-8

**Figure 6:**
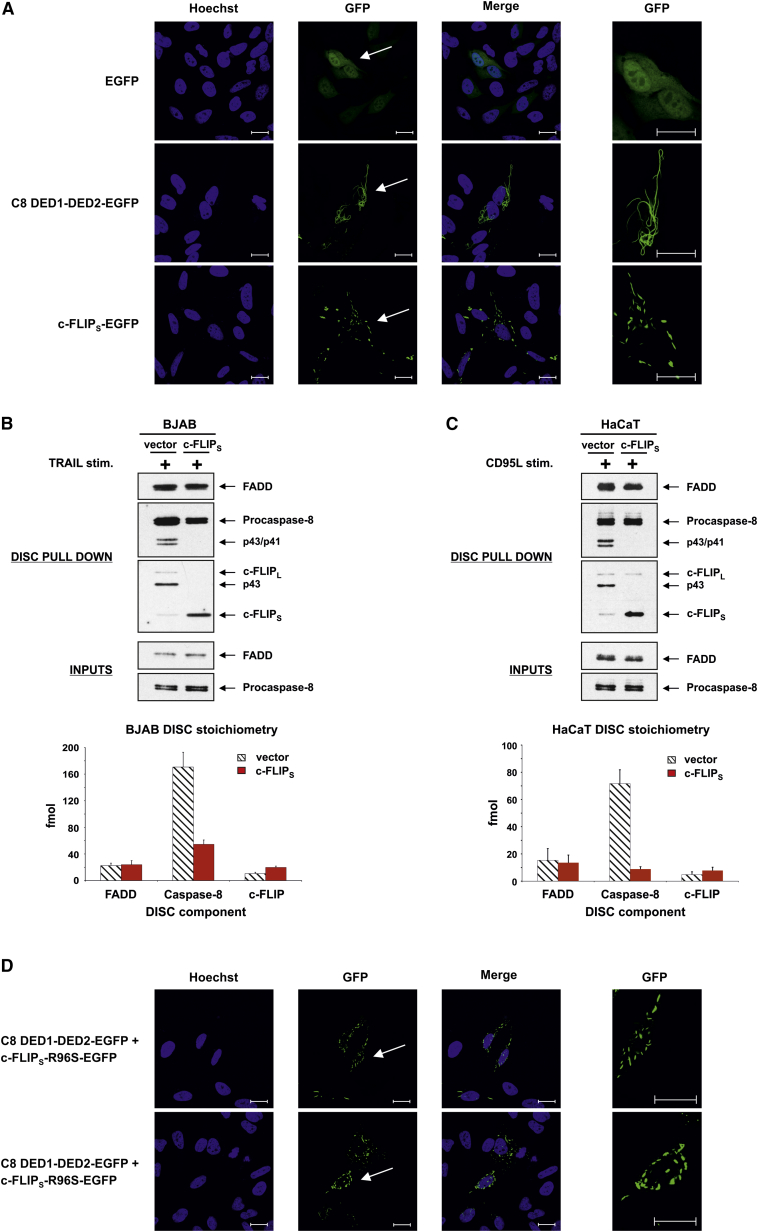
c-FLIP_S_ Blocks Cell Death by Inhibiting DED-Mediated Procaspase-8 Oligomerization (A) HeLa cells were transfected with empty vector (EGFP), GFP-tagged caspase-8 DEDs (C8 DED1-DED2-EGFP), or GFP-tagged c-FLIP_S_ (c-FLIP_S_-EGFP) for 24 hr before fixing and staining with Hoechst. Cells were imaged and a representative field for each transfection is shown. Far right panels show enlargement of areas arrowed in GFP panels. Scale bar, 20 μm. (B) Native TRAIL DISC isolated from 5 × 10^8^ control (vector) and c-FLIP_S_ expressing BJAB cells stimulated with bTRAIL. Affinity-purified TRAIL DISCs and cleared lysate supernatants (Inputs) were analyzed by western blotting. DISCs were analyzed by label-free quantitative LC-MS/MS to determine the amount of FADD, caspase-8, and c-FLIP (lower panel; mean ± SEM; n = 3). (C) Native CD95 DISC isolated from 2 × 10^8^ control (vector) and c-FLIP_S_ expressing HaCaT cells stimulated with Fc-CD95L. DISCs were analyzed and quantified as in (B) (lower panel; mean ± SEM; n = 3). (D) HeLa cells transfected with GFP-tagged caspase-8 DEDs in combination with c-FLIP_S_ tagged with a non-fluorescent EGFP mutant (C8 DED1-DED2-EGFP + c-FLIP_S_-R96S-EGFP) and visualized as in (A). See also Figures S5–S7.

Previous studies have shown that upon transient overexpression, the DEDs of caspase-8, caspase-10, or FADD can interact to form fibers known as death effector filaments (Shen et al., 2015, Siegel et al., 1998, Vajjhala et al., 2015). To explore the propensity of c-FLIP to form death effector filaments and thus mediate DED:DED interactions, we transiently overexpressed the DEDs of procaspase-8 or c-FLIP_S_ in HeLa, MCF-7, and HEK293 cells. Intriguingly, death effector filament formation was clearly observed in cells transfected with procaspase-8 DED1-DED2-EGFP, whereas only short filaments/spot-like structures were detected in cells expressing c-FLIP_S_-EGFP (Figure 6A; Figures S5 and S6). Thus, in contrast to caspase-8 DEDs, c-FLIP_S_ DEDs do not form death effector filaments. This suggested that, unlike procaspase-8, multiple c-FLIP_S_ molecules do not preferentially self-associate and consequently may prevent DED-mediated procaspase-8 oligomerization.

To explore this possibility, we examined the effect of c-FLIP_S_ on procaspase-8 recruitment and native DISC stoichiometry in cells stably expressing c-FLIP_S_. As reported previously, exposure of the Burkitt’s lymphoma cell line, BJAB, to TRAIL (Dickens et al., 2012a), or HaCaT keratinocytes to CD95L (Kavuri et al., 2011) induced receptor-mediated activation of caspase-8, caspase-3, and apoptotic cell death. Treatment of BJAB cells or HaCaT cells stably overexpressing c-FLIP_S_ with TRAIL or CD95L, respectively, completely blocked caspase cleavage and DR-mediated apoptosis (Figure S7A). Analysis of the native TRAIL or CD95 DISC using ligand affinity purification revealed that FADD recruitment to the DISC did not differ between vector control cells and c-FLIP_S_ overexpressing cells (Figures 6B and 6C; Figure S7B). In vector control cells, endogenous c-FLIP_L_ was predominantly detected as a DISC-bound p43 cleavage product, whereas c-FLIP_S_ was only weakly detected (Figures 6B and 6C). In contrast, c-FLIP_S_-overexpressing cells exhibited enhanced levels of c-FLIP_S_ recruitment to the DISC, whereas c-FLIP_L_ was barely detected. Importantly, in line with our previous report (Kavuri et al., 2011), while both procaspase-8 and p43/41 cleavage fragments were detected in control cells, in c-FLIP_S_ overexpressing cells DISC recruitment of procaspase-8 was not affected, but caspase-8 cleavage was abolished (Figures 6B and 6C).

The relative stoichiometry of native DISC core components was analyzed in both cell lines by mass spectrometry and label-free absolute quantification, using the “TOP 3” algorithm (Silva et al., 2006). FADD, caspase-8, and c-FLIP were detected by LC-MS/MS analysis of the TRAIL or CD95 DISC from control and FLIP_S_ overexpressing BJAB (Figure 6B) or HaCaT (Figure 6C) cells, respectively. Label-free absolute quantification revealed that in control cells, both FADD and c-FLIP were sub-stoichiometric compared to caspase-8, with approximately 6-fold more caspase-8 than FADD present within the DISC (Figures 6B and 6C). Importantly, the stoichiometry of FADD:caspase-8 in these cell lines thus agrees with the caspase-8 DED chain model we proposed previously whereby multiple procaspase-8 molecules are recruited to the DISC via a single FADD molecule (Dickens et al., 2012a, Schleich et al., 2012). Strikingly, in the DISC isolated from FLIP_S_ overexpressing BJAB or HaCaT cells, the stoichiometry of FADD:caspase-8:c-FLIP was markedly reduced to ∼1:1:1 (Figures 6B and 6C), with FADD levels remaining similar to those detected in the control DISC. These data show that c-FLIP_S_ inhibits DISC signaling by limiting the number of procaspase-8 molecules within the DISC, suggesting that c-FLIP_S_ functions as a terminator of DED-mediated interactions.

To formally test this hypothesis, we transiently overexpressed the DEDs of procaspase-8 and c-FLIP_S_ in combination, but in the case of c-FLIP_S_ a non-fluorescent mutated form of EGFP was employed. Consistent with our earlier findings, death effector filament formation was consistently observed in cells transfected with procaspase-8 DED1-DED2-EGFP (Figure 6A; Figures S5 and S6). However, transfection of procaspase-8 DEDs in combination with c-FLIP_S_ impeded formation of procaspase-8 death effector filaments, demonstrating that c-FLIP_S_ acts to prevent death effector filament formation (Figure 6D; Figures S5 and S6). Thus, quantitative mass spectrometry as well as co-expression of procaspase-8 DEDs with c-FLIP_S_ reveal that c-FLIP_S_ does not readily support DED-mediated interactions. Our data provide direct evidence for an alternative model whereby c-FLIP_S_ blocks DISC-mediated caspase-8 activation by inhibiting DED-mediated oligomerization of multiple procaspase-8 molecules.

## Discussion

c-FLIP is a major regulator of caspase-8, controlling not only DR-mediated apoptosis but also non-apoptotic caspase-8 signaling. The ability of caspase-8 to effect opposing cellular outcomes within key signaling platforms including the DISC, the necrosome, and the ripoptosome depends on the unique ability of c-FLIP isoforms to regulate caspase-8 function. Thus, while c-FLIP_S_ is widely reported as an inhibitor of caspase-8, c-FLIP_L_ is more complex as it functions as either an activator or an inhibitor of caspase-8 activation.

So far, the mechanisms underlying the ability of c-FLIP isoforms to differentially regulate caspase-8 have not been elucidated, and current models propose that c-FLIP competes with procaspase-8 for binding to FADD. However, this mechanism has been largely determined from overexpression studies with the assumption that c-FLIP and procaspase-8 bind to FADD in a similar manner (Fricker et al., 2010, Kavuri et al., 2011, Krueger et al., 2001b, Scaffidi et al., 1999). Moreover, the majority of biochemical studies have used either truncated proteins lacking the N-terminal DED domains of c-FLIP_L_ or caspase-8 (Boatright et al., 2004, Keller et al., 2009) or fusion proteins where the pro-domains are replaced by FKBP/FRB heterodimerization domains (Chang et al., 2002, Pop et al., 2011). Importantly, these studies have not addressed how c-FLIP can regulate caspase-8 in the presence of the critical adaptor molecule FADD or within the DISC or other signaling complexes.

To address this, we reconstituted the DISC using full-length recombinant proteins and structure-guided DED mutants. This approach revealed how c-FLIP is recruited to FADD, how different c-FLIP isoforms regulate procaspase-8 activation within its native activation complex, and importantly how this controls cell fate. Our data reveal a fundamental mechanism involving co-operative but hierarchical recruitment of procaspase-8:c-FLIP_L/S_ to FADD where c-FLIP_S_ inhibits DED-mediated caspase-8 oligomerization. Significantly, our data provide firm evidence that procaspase-8:c-FLIP_L_ heterodimers initiate procaspase-8 activation within the native DISC and crucially explain the unique ability of c-FLIP_L_ to promote or inhibit cell death.

Our unique approach using reciprocal binding of individual components in the reconstituted DISC revealed that, contrary to current dogma, c-FLIP does not compete directly with procaspase-8 for binding to FADD (Figure 3). Instead, without caspase-8, c-FLIP binding to FADD is limited, but recruitment to the complex is significantly enhanced when procaspase-8 is present (Figure 3). Crucially, this demonstrates that procaspase-8 binding to FADD is the key initiating event that enables c-FLIP recruitment via c-FLIP heterodimerization with procaspase-8. We validated this procaspase-8:c-FLIP_L/S_ co-operative binding model in a cellular context, using lysates from caspase-8-deficient Jurkat cells, and showed that, without caspase-8, endogenous c-FLIP_L/S_ was not recruited to the DISC (Figure 3). However, addition of exogenous procaspase-8 restored recruitment of both endogenous c-FLIP_L_ and c-FLIP_S_ to the complex.

Mutating key residues in procaspase-8 showed that procaspase-8 recruitment to FADD occurs predominantly via procaspase-8 DED1 pocket (Figure 4). In contrast, in the absence of procaspase-8, the limited c-FLIP binding to FADD is mediated via c-FLIP DED2 (Figure 5). Interestingly, mutation of the pocket residue H7 in c-FLIP DED1 did not affect co-operative binding of c-FLIP via procaspase-8. Hence, c-FLIP pocket residue H7, which apparently plays a role in c-FLIP homodimerization, does not participate in procaspase-8-mediated recruitment of c-FLIP to the DISC (Figure S4). This suggests that the procaspase-8 DED2:c-FLIP DED1 interaction is somewhat different from the canonical procaspase-8 DED2:DED1 oligomer interface. Further evidence that c-FLIP recruitment to the complex occurs predominantly via heterodimerization with procaspase-8 is provided by our studies with the FADD DED pocket mutant, which does not recruit c-FLIP unless procaspase-8 is present (Figure 5). This co-operative and hierarchical binding mechanism for FADD recruitment of caspase-8 and c-FLIP clearly challenges existing paradigms (Irmler et al., 1997, Majkut et al., 2014), thus raising the important question of how c-FLIP_L/S_ modulate procaspase-8 activation/activity to produce diverse signaling outcomes.

**Figure 7:**
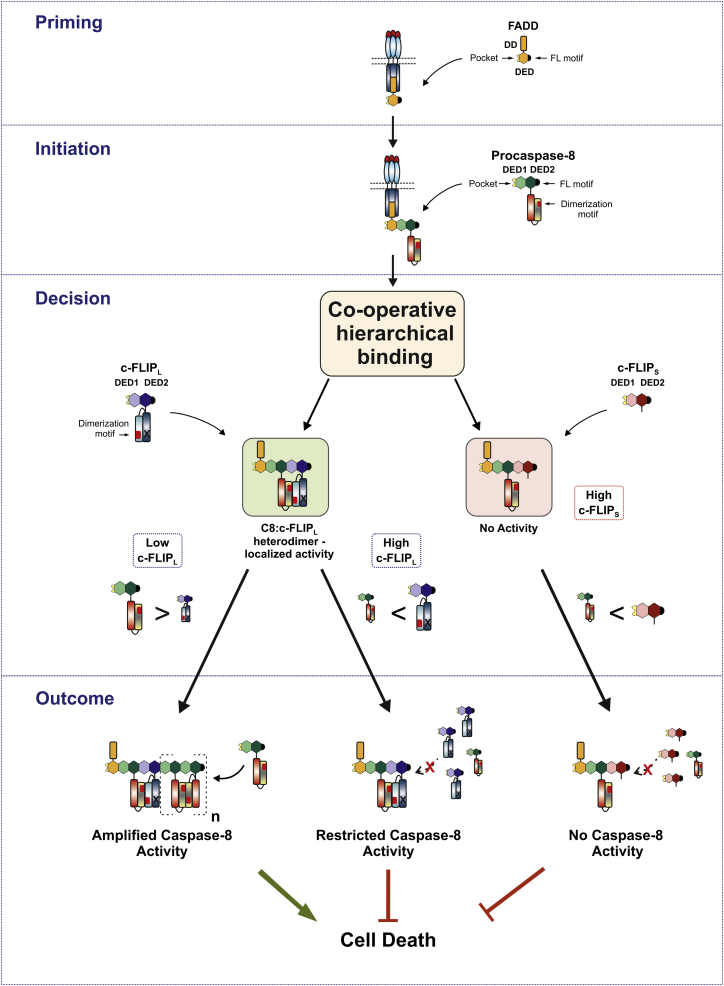
A Unified Model Defines How c-FLIP Isoforms Differentially Regulate Procaspase-8 Activation to Direct Cell Fate In this model, the priming event is FADD recruitment to ligated DR. Initiation proceeds via procaspase-8 recruitment to FADD, with procaspase-8 DED1 pocket binding to FADD FL motif. Procaspase-8, in turn, recruits and heterodimerizes with c-FLIP_L/S_ via a co-operative and hierarchical binding mechanism. The composition of this procaspase-8:c-FLIP heterodimer then constitutes a key decision step, which determines procaspase-8 activation and subsequent cell fate. Heterodimer composition is critically regulated by the ratio of unbound c-FLIP_L/S_ to procaspase-8; thus, at physiological levels, procaspase-8:c-FLIP_L_ heterodimer forms the first active protease at the DISC, exhibits localized activity and is an activator, promoting procaspase-8 oligomer assembly and cell death. In contrast, high levels of c-FLIP_L_ preclude procaspase-8 oligomer assembly, restricting caspase-8:c-FLIP_L_ heterodimer activity and inhibiting cell death. c-FLIP_S_ does not readily form DED oligomers; thus, high levels of c-FLIP_S_ disrupt procaspase-8 oligomer assembly, resulting in a catalytically inactive procaspase-8:c-FLIP_S_ heterodimer and inhibition of cell death.

Our reconstituted DISC experiments fully recapitulated the suggested biphasic effects of c-FLIP_L_ on procaspase-8 function (Chang et al., 2002, Fricker et al., 2010, Micheau et al., 2002). Thus, c-FLIP_L_ can activate or inhibit procaspase-8, highlighting that the ratio of c-FLIP_L_ to procaspase-8 critically determines procaspase-8 signaling. Intriguingly, c-FLIP_L_-mediated inhibition of caspase-8 was only observed at high concentrations of c-FLIP_L_ relative to caspase-8, demonstrating that at physiological concentrations (c-FLIP levels are reportedly only 1% of that of caspase-8; Scaffidi et al., 1999) c-FLIP_L_ preferentially activates procaspase-8 (Figure 2). c-FLIP_L_-mediated activation of caspase-8 depends on procaspase-8:c-FLIP_L_ heterodimerization, resulting in localized enzymatic activity in the absence of proteolytic cleavage of either caspase-8 or c-FLIP_L_ (Figures 1 and 2). These findings are supported by structural studies of truncated c-FLIP_L_-caspase-8 heterodimer, which show that c-FLIP_L_ binding to non-cleavable caspase-8 allows rearrangement of the catalytic loops, forming an active site without processing (Boatright et al., 2004, Yu et al., 2009). Thus, loss of c-FLIP_L_ heterodimerization activity renders c-FLIP_L_ solely an inhibitor of procaspase-8 (Figure 2; data not shown). Intriguingly, our discovery that c-FLIP recruitment to FADD is indirect and requires procaspase-8 supports a model where FADD recruitment of procaspase-8 initiates preferential recruitment of c-FLIP_L_ rather than a second procaspase-8 molecule. Thus, in our model, this initiation step enables procaspase-8:c-FLIP_L_ heterodimers to form the first active protease at the DISC (Figure 7). As levels of c-FLIP_L_ become depleted, DED-mediated procaspase-8 recruitment then proceeds, facilitating proximity-induced activation and proteolytic cleavage of procaspase-8. Evidence for this mechanism of hierarchical imprinting of DED:DED oligomers, with procaspase-8 activation primed by procaspase-8:c-FLIP_L_ heterodimers, comes from quantitative mass spectrometry of the native CD95/TRAIL DISC where c-FLIP_L_ is consistently substoichiometric relative to caspase-8, with approximately 15-fold less c-FLIP_L_ than caspase-8 detected within the active DISC (Figure 6). Thus, procaspase-8:c-FLIP_L_ heterodimers comprise only a minor component of the native DISC, and even high cellular levels of c-FLIP_L_ that inhibit cell death do not shift the stoichiometry in favor of c-FLIP_L_ (data not shown).

In the reconstituted DISC, c-FLIP_S_ is a potent inhibitor of caspase-8 as it results in concentration-dependent inhibition of DISC-mediated procaspase-8 activation (Figure 2). Furthermore, native TRAIL and CD95 DISCs isolated from c-FLIP_S_-overexpressing cells (Figure 6) show that c-FLIP_S_ completely blocks procaspase-8 processing at the complex, downstream caspase-3 activation, and apoptotic cell death (Figure S7). Our experiments with c-FLIP_S_ and the reconstituted DISC led us to investigate how c-FLIP_S_ so potently antagonizes caspase-8 activation (Irmler et al., 1997, Kavuri et al., 2011, Krueger et al., 2001a, Rasper et al., 1998, Scaffidi et al., 1999) yet requires procaspase-8 for initial recruitment to the DISC. Using quantitative mass spectrometry we determined the stoichiometry of c-FLIP_S_ and caspase-8 in DISCs isolated from c-FLIP_S_-overexpressing cells, showing that DR-mediated apoptosis was inhibited. Significantly, the ratio of FADD:caspase-8:c-FLIP in the DISC was now ∼1:1:1. This unexpected finding challenges current models (which would predict the stoichiometry to shift in favor of c-FLIP_S_) and led us to hypothesize that procaspase-8:c-FLIP_S_ heterodimers are catalytically inactive and moreover inhibit DISC signaling by modulating DED-mediated caspase-8 oligomerization. Our observation that only a finite amount of c-FLIP_S_ is recruited to a reconstituted DISC (Figure 3) suggested that c-FLIP_S_ precludes DED oligomer assembly. In support of this, c-FLIP_S_, like its viral homolog MC159 (Siegel et al., 1998), does not preferentially self-associate to form DED filaments and furthermore blocks caspase-8 DED filament formation (Figure 6; Figures S5 and S6). Taken together, our studies show that c-FLIP_S_ inhibits caspase-8 activation by disrupting DED-mediated procaspase-8 oligomer assembly, thereby preventing functional alignment of the catalytic dimers (p18_2_/p10_2_) (Figure 7). This alternative mechanism reveals how c-FLIP_S_ prevents both proximity-induced activation and proteolytic cleavage of caspase-8, thereby abrogating apoptotic cell death. Crucially, procaspase-8:c-FLIP co-operative binding enables c-FLIP_S_ to block both DISC-mediated oligomerization and activation of procaspase-8, thus explaining why c-FLIP_S_ is such a potent inhibitor of caspase-8.

Significantly, our procaspase-8:c-FLIP co-operative binding model explains the differential potency of c-FLIP_L_ and c-FLIP_S_ to inhibit procaspase-8 activation. Thus, c-FLIP_S_ potently blocks procaspase-8 processing, but very high levels of c-FLIP_L_ are required to inhibit caspase-8 (Figure 2). In this case, procaspase-8:c-FLIP_L_ heterodimers would trigger initial activation and cleavage of procaspase-8 within the complex (Kavuri et al., 2011, Krueger et al., 2001a, Micheau et al., 2002), and as a result high concentrations of c-FLIP_L_ would be required in order to block subsequent DED-mediated procaspase-8 oligomer assembly. Thus, the balance of c-FLIP_L/S_ to procaspase-8 is critical in determining signaling outcome, and a shift in the stoichiometry of these molecules determines signaling for death or survival (Figure 7).

The co-operative and hierarchical binding model we now propose for c-FLIP regulation of caspase-8 in the context of DR signaling may also be applicable to other FADD:caspase-8:c-FLIP-containing signaling complexes where the ratio of c-FLIP isoforms to procaspase-8 determines cell survival or death. Thus, c-FLIP regulation of caspase-8 is required in T cells, where levels of c-FLIP isoforms modulate cell fate during development of the immune response (Hinshaw-Makepeace et al., 2008, Koenig et al., 2014). Moreover, a shift in the stoichiometry of c-FLIP isoforms and caspase-8 in signaling platforms such as the RIPK1-RIPK3 necrosome, where caspase-8:c-FLIP_L_ heterodimers suppresses RIPK3-dependent necrosis, would be predicted to have major consequences in vivo. Importantly, our procaspase-8:c-FLIP co-operative binding model provides a long sought after mechanism that uniquely enables c-FLIP_S_ inhibition of DED-mediated procaspase-8 oligomerization/activation as well as accommodating and explaining the dual functionality of c-FLIP_L_. This single regulatory mechanism can directly modulate downstream caspase activation and cell fate, thus providing a unified model for c-FLIP regulation of procaspase-8 with signaling outcome critically determined by the ratio of c-FLIP isoforms to procaspase-8.

## Experimental Procedures

The Supplemental Experimental Procedures detail expression constructs, antibodies, western blot analysis, apoptosis assays, in vitro GST pull downs, and LC-MS/MS analysis.

### DISC Reconstitution

Complete reconstitutions were carried out as described (Hughes et al., 2009), with the following modifications. Beads coated with purified GST-TRAIL-R1/R2-IcD or GST-CD95-IcD (10 μg) (Harper et al., 2003) were incubated with r-FADD (5 μg), and the indicated amounts of IVT-produced procaspase-8b (^35^S-labeled) plus/minus c-FLIP_L/S_, for 16 hr at 20°C (unless indicated otherwise). Control reconstitutions contained beads coated with GST alone. Bead-associated complexes were analyzed by SDS-PAGE/western blotting. r-DISC-associated caspase-8 activity was measured by cleavage of the fluorogenic substrate Ac-IETD.AFC or by bioassay using recombinant procaspase-3 active-site mutant (C163A) or Bid (Hughes et al., 2013).

### TRAIL and CD95 DISC Analysis

Biotin-labeled TRAIL (bTRAIL) was generated and TRAIL DISC affinity purified from control or c-FLIP_S_-expressing BJAB cells as described (Dickens et al., 2012a, Hughes et al., 2013). CD95 DISC was affinity purified from control or c-FLIP_S_-expressing keratinocytes (HaCaT cells) as described (Kavuri et al., 2011), with the following modifications. 5 × 10^6^ HaCaT cells were incubated with 250 U/ml Fc-CD95L for 30 min at 37°C, washed with PBS, and lysed with 2 ml lysis buffer (30 mM Tris-HCl [pH 7.5], 150 mM NaCl, 10% [v/v] glycerol, 1% Triton X-100 [v/v], containing Complete protease inhibitors [Roche Applied Science]) for 60 min at 4°C. DISC complexes were analyzed by western blotting or mass spectrometry.

### Quantitative Mass Spectrometry

LC-MS/MS was used to identify and quantify DISC proteins (see Supplemental Experimental Procedures). Briefly, TRAIL and CD95 DISC proteins were separated by SDS-PAGE and serial gel slices digested in situ with trypsin (Dickens et al., 2012a). Extracted tryptic peptides were analyzed using data-independent acquisition (DIA) on a nanoAcquity UPLC system coupled to a Waters Synapt G2-S HDMS mass spectrometer. The PLGS “TOP 3” method was used for absolute quantification of proteins (Silva et al., 2006).

### Transfection and Visualization of Death Effector Filaments

HeLa, MCF-7, or HEK293 cells were seeded onto coverslips (coated with gelatin or poly-D-Lysine) 24 hr prior to transfection. Cells were transfected in the presence of the pan-caspase inhibitor, zVAD.FMK (50 μM), and fixed as described (MacFarlane et al., 2000, Dickens et al., 2012a); EGFP alone served as control. Image analysis was performed with Zen 2009 (Zeiss).

### Structural Modeling

The modeled structure of the DEDs of caspase-8 (amino acids 1–183; UniProtKB: Q14790) was generated as described (Dickens et al., 2012a). Amino acids 1–177 of c-FLIP (UniProtKB: O15519) were submitted to the Phyre^2^ server, and the structure resulting from threading of this sequence through the published structure of MC159 (PDB: 2BBR) was selected as our modeled structure of c-FLIP DED1/2. The DED domains of caspase-8, c-FLIP, and FADD (PDB: 2GF5) were aligned using LSQman. Pymol (www.pymol.org) was used to generate structural models.

## Author Contributions

M.M., M.A.H., and K.C. conceived the study, designed experiments, and analyzed results. M.A.H. carried out the majority of experiments; I.R.P., confocal microscopy; R.J.-J., LC-MS/MS; S.H. and M.F., c-FLIP overexpression; L.F. and J.W.R.S., structural modeling. M.M., M.A.H., M.L., and K.C. wrote the manuscript with input from all co-authors.

## Supplemental Information

Document S1. Supplemental Experimental Procedures and Figures S1–S7

Document S2. Article plus Supplemental Information
