# Supplementary material for: Co-operative and Hierarchical Binding of c-FLIP and Caspase-8: A Unified Model Defines How c-FLIP Isoforms Differentially Control Cell Fate
Source: Mol Cell. 2016 Mar 17;61(6):834–49. doi: 10.1016/j.molcel.2016.02.023 (PMC4819448; doi:10.1016/j.molcel.2016.02.023)
Supplement: Document S1. Supplemental Experimental Procedures and Figures S1–S7 [file mmc1.pdf]

Molecular Cell, Volume *61*

Supplemental Information

Co-operative and Hierarchical Binding of c-FLIP and Caspase-8: A Unified Model Defines How c-FLIP Isoforms Differentially Control Cell Fate

Michelle A. Hughes, Ian R. Powley, Rebekah Jukes-Jones, Sebastian Horn, Maria Feoktistova, Louise Fairall, John W.R. Schwabe, Martin Leverkus, Kelvin Cain, and Marion MacFarlane

Supplemental Figures

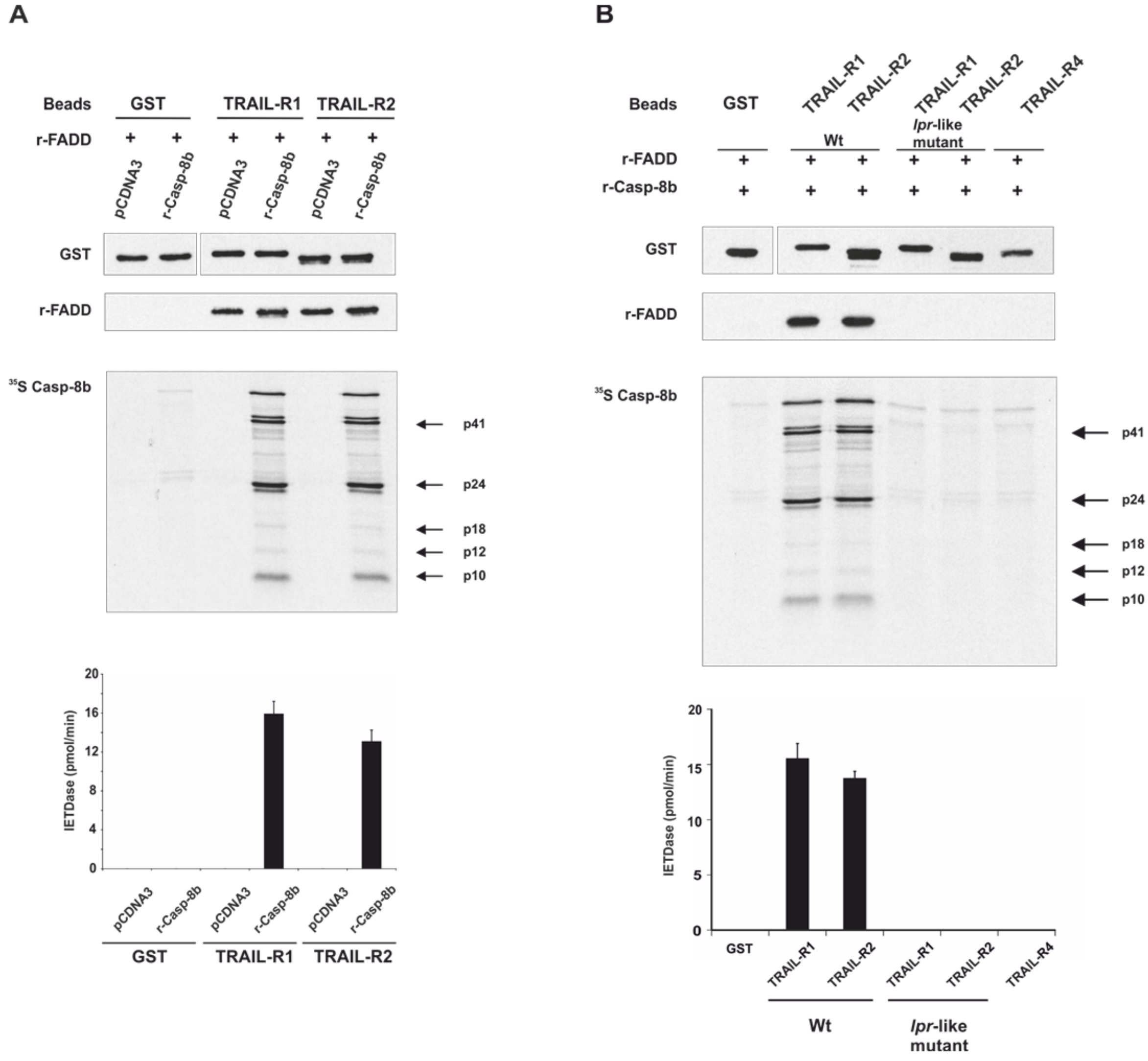

Figure S1, related to Figure 1. Reconstitution of a functional TRAIL-R1/R2 DISC is achieved only in the presence of Wt TRAIL-R1/R2-IcDs

(**A**) A functional DISC (r-DISC) was reconstituted using GST-TRAIL-R1/R2 intracellular domains (TRAIL-R1/R2-IcD) (10 µg), recombinant FADD (r-FADD) (5 µg) and ^{35}S-labeled recombinant procaspase-8b (^{35}S r-Casp-8b; 100 µl) at 20°C for 16 h. ^{35}S-labeled empty vector (pcDNA3) and GST beads were used as controls. Beads were analyzed by SDS-PAGE and autoradiography for ^{35}S r-Casp-8b and immunoblotted for GST and FADD. Beads were also assayed for caspase-8 (IETDase) activity (Mean ± SEM; n=3). (**B**) r-DISCs were assembled using wild-type (Wt) or DD mutant (*lpr*-like) TRAIL-R1/R2-IcDs or TRAIL-R4 (10 µg), r-FADD (5 µg) and ^{35}S r-Casp-8b (100 µl). GST beads were used as control. Beads were analyzed as described in (A). Equal input of GST and GST-TRAIL-R-IcD was confirmed by immunoblotting for GST. *lpr*-like mutations (mature receptor numbering): L366A (TRAIL-R1) and L283A/L312A (TRAIL-R2 short/long isoform).

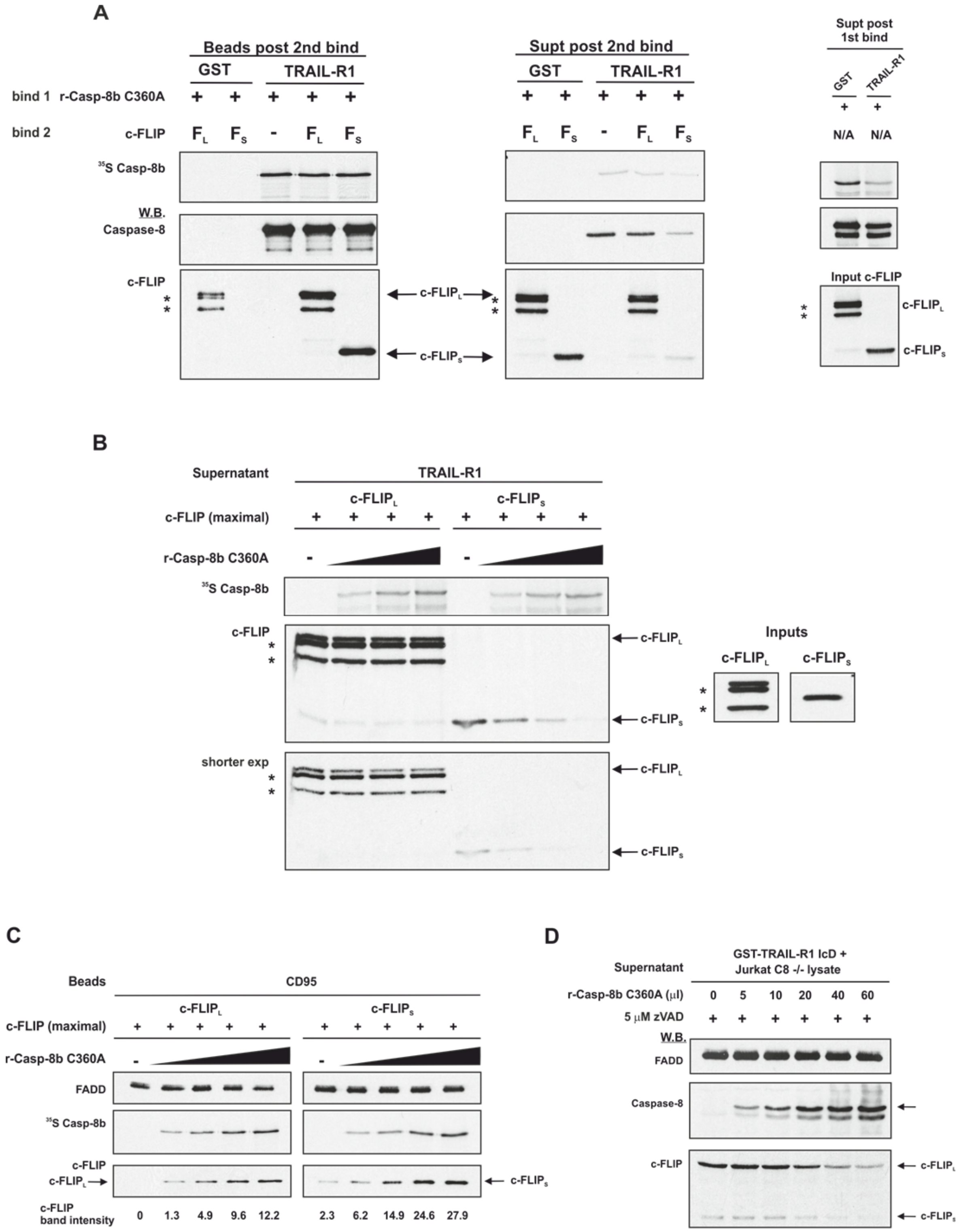

Figure S2, related to Figure 3. DISC reconstitution reveals co-operative and hierarchical recruitment of c-FLIP$_{L/S}$ and procaspase-8

(**A**) c-FLIP$_L$ or c-FLIP$_S$ do not displace procaspase-8 from FADD. The r-DISC was assembled at 16°C for 16 h using TRAIL-R1-IcD, r-FADD and ^{35}S r-Casp-8b C360A (160μl). r-DISC beads were then washed with PBS before incubating with c-FLIP$_{L/S}$ (F_L, F_S; 150 μl) for 16 h at

16°C. Following the second bind, beads and supernatants were assessed for ^{35}S r-Casp-8b and immunoblotted for caspase-8 and c-FLIP. Analysis of the supernatants following r-DISC assembly (supt post 1st bind) is shown in the right hand panel. Equal c-FLIP$_{L/S}$ inputs were confirmed by immunoblotting and GST beads served as control. (**B**) Procaspase-8 enhances c-FLIP$_{L/S}$ recruitment to the r-DISC. The r-DISC was assembled with c-FLIP$_{L/S}$ alone (-) or with increasing amounts of ^{35}S r-Casp-8b C360A. The supernatants (free/unbound proteins) were analyzed for caspase-8 (upper panel) and c-FLIP$_{L/S}$ (lower panels). The corresponding bead-bound r-DISC is shown in Figure 3B. (**C**) The CD95 r-DISC was assembled using GST-CD95-IcD (10 µg), r-FADD (5 µg) and c-FLIP$_{L/S}$ (85 µl) alone (-) or in combination with increasing amounts of ^{35}S r-Casp-8b C360A (0 – 75 µl). Beads were analyzed for FADD (upper panel), caspase-8 (middle panel) and c-FLIP$_{L/S}$ binding (lower panel). *non-specific band. (**D**) TRAIL-R1-IcD pull downs were performed with caspase-8 null Jurkat lysates (10 mg) alone, or in combination with increasing amounts of r-procaspase-8b (r-Casp-8b C360A; 0 – 60 µl) in the presence of zVAD.fmk (5 µM). Supernatants (free/unbound proteins) were analyzed for FADD, procaspase-8 and c-FLIP$_{L/S}$. The corresponding bead-bound complexes are shown in Figure 3D.

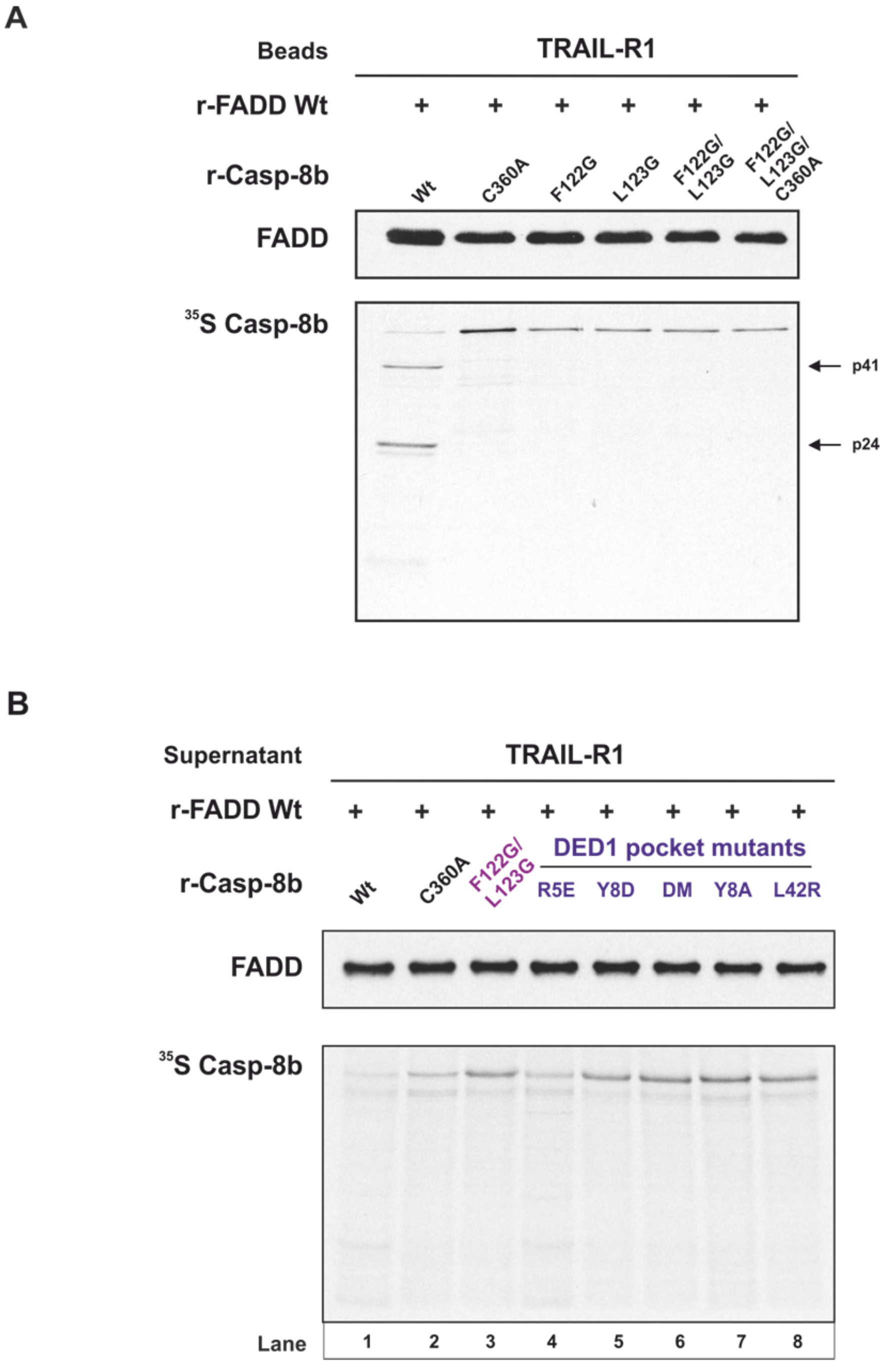

Figure S3, related to Figure 4. Mutation of caspase-8 DED2 F122 or L123 is sufficient to reduce procaspase-8 binding to the r-DISC

(**A**) The r-DISC was assembled using TRAIL-R1-IcD, r-FADD and ^{35}S r-Casp-8b wild-type (Wt), active site mutant (C360A) or DED2 FL motif mutants (F122G; L123G; F122G/L123G; F122G/L123G/C360A) (100 µl). Beads were analyzed for FADD and procaspase-8 binding. (**B**) The r-DISC was reconstituted using the indicated variants of ^{35}S r-Casp-8b (100 µl) and supernatants (free/unbound proteins) assessed for FADD (upper panel) and r-Casp-8b (lower panel). The corresponding bead-bound complexes are shown in Figure 4C.

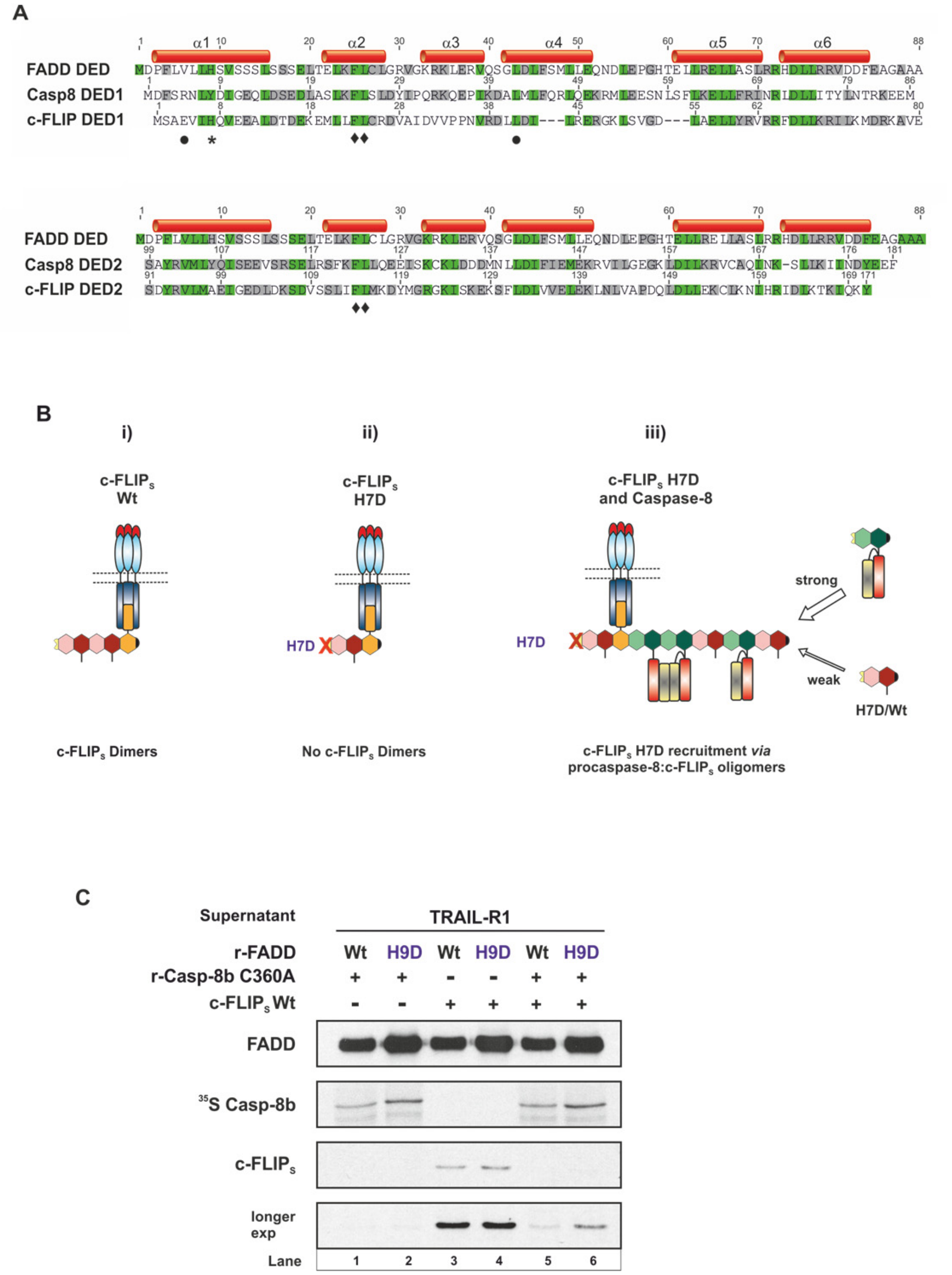

Figure S4, related to Figure 5. c-FLIP and procaspase-8 are recruited to the DISC *via* molecularly distinct mechanisms

(**A**) Sequence alignment of FADD DED with DED1 (upper panel) and DED2 (lower panel) of c-FLIP and caspase-8 performed using CLUSTALW2 (EMBL-EBI). Residues with 100%

similarity are shaded green and 80 - 99% shaded grey. Residues at the conserved hydrophobic patch (FL motif) are marked by ♦. Residues mutated in the DED1 pocket motif of caspase-8, c-FLIP and FADD are indicated by ∗; Pocket residues mutated only in caspase-8 are marked by •. Secondary structures (α-helices) illustrated above the sequences are based on the structure of FADD DED. (**B**) Schematic depicting DISC recruitment of c-FLIP DED1 pocket mutant, in the absence or presence of procaspase-8. In the absence of caspase-8, limited c-FLIP (Wt) recruitment to FADD is mediated *via* the pocket motif of FADD DED and the DED2 FL motif of c-FLIP (i). c-FLIP DED1 pocket mutant (H7D) is still recruited to FADD but is unable to recruit additional c-FLIP molecules (ii). In the presence of procaspase-8, c-FLIP recruitment to the DISC is significantly enhanced and recruitment of c-FLIP DED1 H7D mutant *via* caspase-8:c-FLIP DED interactions is not impaired. c-FLIP does not readily self-associate to form DED oligomers (iii). (**C**) c-$FLIP_S$ is recruited to an r-DISC containing a pocket mutant of FADD, only in the presence of procaspase-8. The r-DISC was assembled using wild-type (Wt) or DED pocket mutant (H9D) of r-FADD (5 µg) with ^{35}S r-Casp-8b C360A (100 µl) and wild-type (Wt) c-$FLIP_S$ (50 µl) either singly or in combination. Supernatants (free/unbound proteins) were assessed for FADD, procaspase-8 and c-$FLIP_S$. The corresponding bead-bound complexes are shown in Figure 5C.

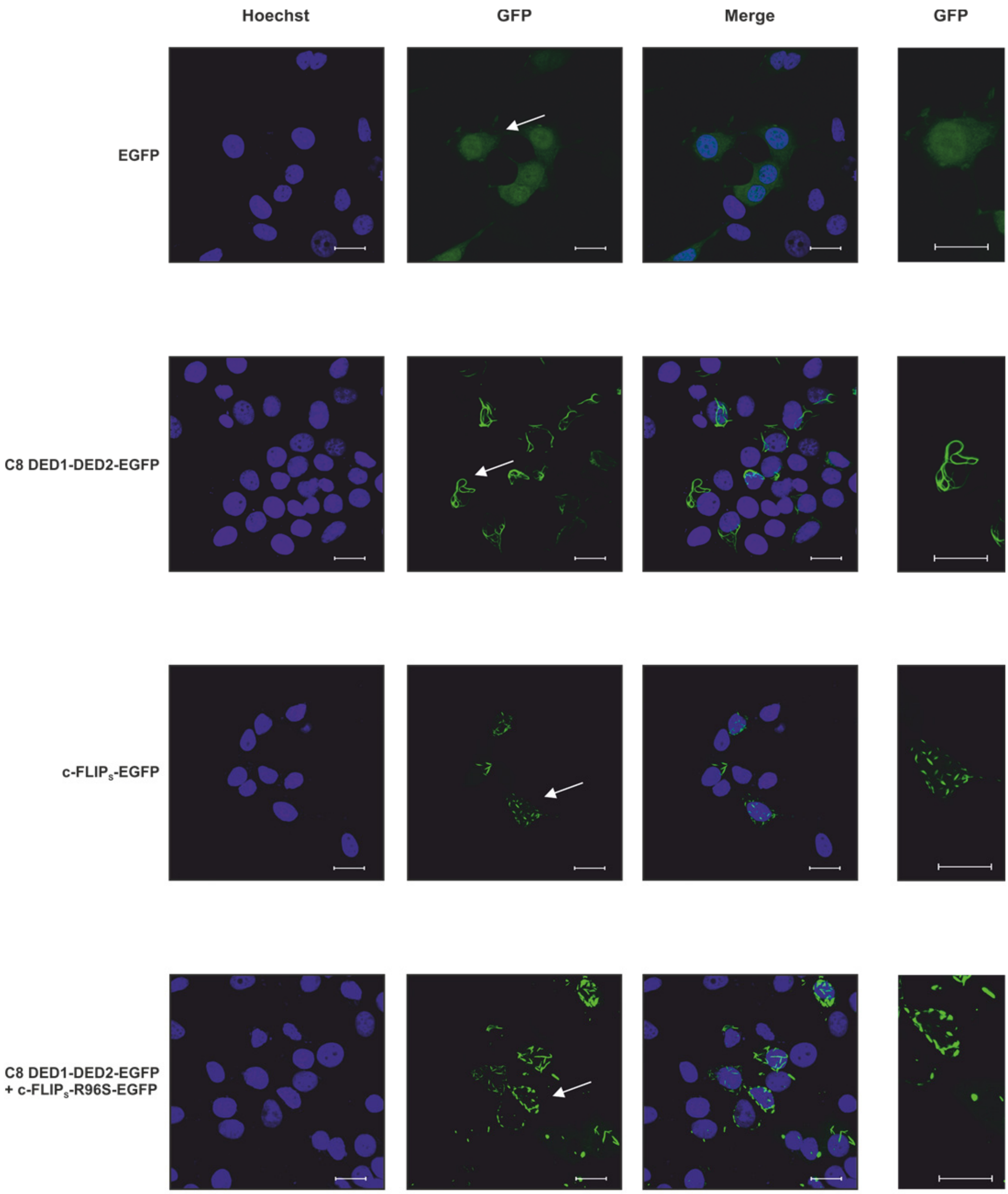

Figure S5, related to Figure 6. c-FLIP$_S$ blocks caspase-8 DED filament formation in MCF-7 cells

MCF-7 cells were transfected with empty vector (EGFP), GFP-tagged caspase-8 DEDs (C8 DED1-DED2-EGFP), GFP tagged c-FLIP$_S$ (c-FLIP$_S$-EGFP) or GFP-tagged caspase-8 DEDs in combination with c-FLIP$_S$ tagged with a non-fluorescent mutant of EGFP (C8 DED1-DED2-EGFP + c-FLIP$_S$-R96S-EGFP) for 24 h before fixing and staining with Hoechst. Cells were imaged using a Zeiss LSM510 confocal microscope, and a representative field for each transfection is shown. Far right panels show enlargement of areas arrowed in GFP panels. Scale bar, 20 μm.

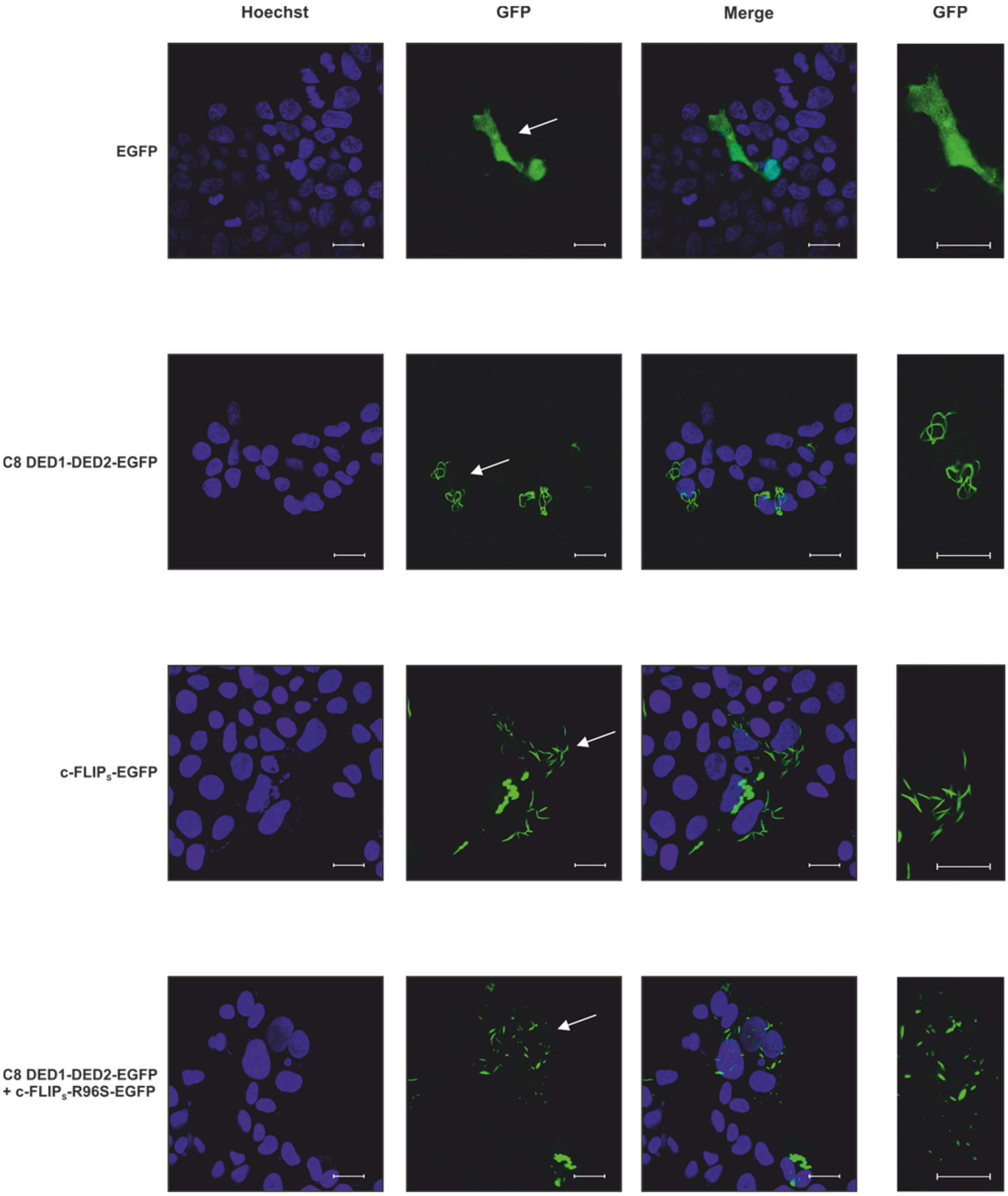

Figure S6, related to Figure 6. c-FLIP$_S$ blocks caspase-8 DED filament formation in HEK293 cells

HEK293 cells were transfected with empty vector (EGFP), GFP-tagged caspase-8 DEDs (C8 DED1-DED2-EGFP), GFP tagged c-FLIP$_S$ (c-FLIP$_S$-EGFP) or GFP-tagged caspase-8 DEDs in combination with c-FLIP$_S$ tagged with a non-fluorescent mutant of EGFP (C8 DED1-DED2-EGFP + c-FLIP$_S$-R96S-EGFP) for 24 h before fixing and staining with Hoechst. Cells were imaged using a Zeiss LSM510 confocal microscope, and a representative field for each transfection is shown. Far right panels show enlargement of areas arrowed in GFP panels. Scale bar, 20 µm.

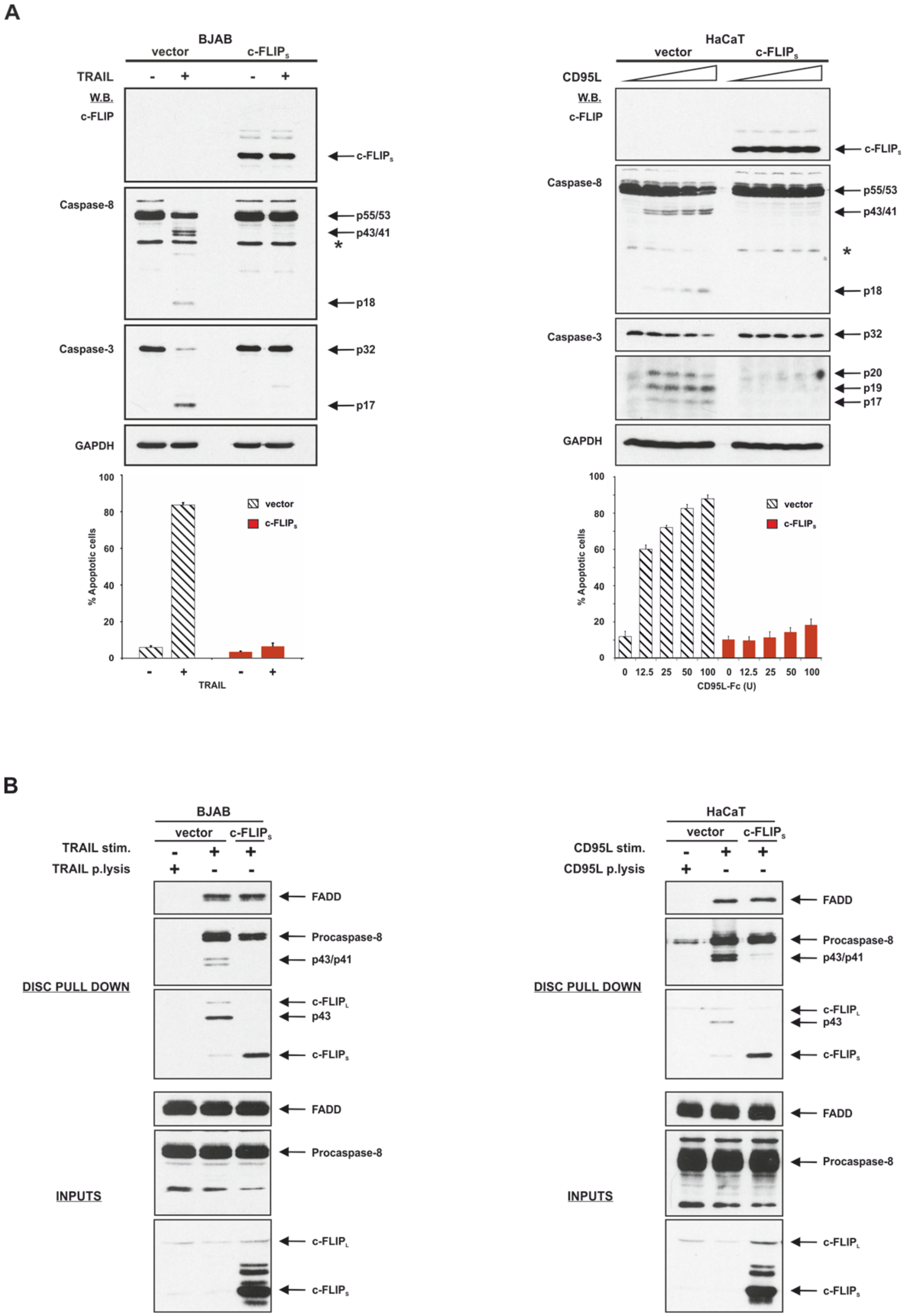
A
BJAB
vector
c-FLIPs
TRAIL
W.B.
c-FLIP
c-FLIPs
Caspase-8
p55/53
p43/41
*
p18
Caspase-3
p32
p17
GAPDH
% Apoptotic cells
vector
c-FLIPs
TRAIL
HaCaT
CD95L
Caspase-8
p55/53
p43/41
p18
Caspase-3
p32
p20
p19
p17
GAPDH
0 12.5 25 50 100 0 12.5 25 50 100
CD95L-Fc (U)
B
BJAB
TRAIL stim.
TRAIL p.lysis
FADD
Procaspase-8
p43/p41
c-FLIPL
p43
c-FLIPs
DISC PULL DOWN
INPUTS
HaCaT
CD95L stim.
CD95L p.lysis
FADD
Procaspase-8
p43/p41
c-FLIPL
p43
c-FLIPs
DISC PULL DOWN
INPUTS

Figure S7, related to Figure 6. c-FLIP$_S$ blocks CD95 or TRAIL-induced apoptosis by inhibiting DED-mediated procaspase-8 oligomerization.

(**A**) Control (vector) and c-FLIP$_S$ expressing BJAB cells were stimulated for 4 h with 1μg/ml TRAIL (left panel). Control (vector) and c-FLIP$_S$ expressing HaCaT cells were stimulated for 24 h with increasing amounts of CD95L (0 – 100 U/ml) (right panel). Cells were analyzed by immunoblotting for c-FLIP, caspase-8 and caspase-3; GAPDH was included as loading control. Following treatment, apoptotic cell death was assessed by PS externalization (Mean ± SEM; n=3); control cells (hatched bars), c-FLIP$_S$ expressing cells (red bars). *non-specific band. (**B**) Native TRAIL or CD95 DISCs were isolated from control and c-FLIP$_S$ expressing BJAB or HaCaT cells, respectively. DISCs and cleared lysate supernatants (Inputs) were analyzed for FADD, caspase-8, and c-FLIP. Precipitates of non-stimulated cells served as controls (TRAIL p.lysis; CD95L p.lysis).

Supplemental Experimental Procedures

Materials - Media and serum were purchased from Invitrogen (Paisley, UK). Antibodies were sourced as follows: FADD mouse monoclonal antibody (mAb) was from BD Transduction Laboratories; GST tag mouse mAb was from Novagen; Myc mouse mAb antibody, Bid rabbit polyclonal antibody, and cleaved caspase-3 (Asp175) rabbit polyclonal antibody were from Cell Signaling; caspase-8 mouse mAb (C15) and c-FLIP mouse mAb (NF-6) were from Enzo® Life Sciences. c-FLIP$_L$ p43 cleavage fragment was detected with NF-6 mAb and c-FLIP$_L$ p12 cleavage fragment with Myc mAb. Horseradish peroxidase-conjugated secondary antibodies were obtained from Sigma (goat anti-mouse) and DAKO (goat anti-rabbit). HRP-conjugated goat anti-mouse IgG1 and IgG2b were from Southern Biotechnology. The generation of Fc-CD95L was described previously (Geserick et al., 2008). Annexin V-APC was purchased from Life Technologies Ltd. The caspase-8 substrate, Ac-Ile-Glu-Thr-Asp-amino-4-trifluoromethyl coumarin (Ac-IETD.AFC) and the caspase inhibitor z-Val-Ala-Asp(OME)-FMK (zVAD.FMK) were from MP Biomedicals. Recombinant human Bid was from R&D Systems. All other chemicals were of analytical grade and obtained from Sigma-Aldrich or Fisher.

Expression constructs - GST-TRAIL-R1/R2-IcD and CD95-IcD fusion proteins were generated as described previously (Harper et al., 2003). Full length FADD (r-FADD) was generated as described previously (Hughes et al., 2009). Full length procaspase-8b (MACHα2/Mch5b) was cloned in pcDNA3.1 (Invitrogen) and untagged proteins produced by *in vitro* transcription/translation (IVT) (Insect System (Qiagen)), incorporating ^{35}S-methionine (Amersham) (Hughes et al., 2009). Mutations were made using the Stratagene QuikChange Site-Directed Mutagenesis kit and confirmed by DNA sequencing. Proteins from c-FLIP$_{L/S}$-Myc in pcDNA6.1 were produced by IVT (TNT T7-coupled reticulocyte lysate system (Promega)).

Cell culture – Caspase-8-deficient Jurkat T-cells (I9.2) (kind gift from J. Blenis), MCF-7-Fas (MCF-7) cells (kind gift from M. Jattella) or BJAB cell lines stably expressing pCFG5 vector or c-FLIP$_S$ were maintained in RPMI medium, supplemented with 10% fetal bovine serum and 2 mM Glutamax™. HeLa cells, HEK293 cells (both ECACC), or HaCaT cell lines stably expressing pCFG5 vector or c-FLIP$_S$ (Geserick et al., 2009), were maintained in DMEM medium, supplemented with 10% fetal bovine serum. Cells were grown in an atmosphere of 5% CO2 in air at 37°C and maintained in logarithmic growth phase by routine passage every 3-4 days.

Determination of Apoptosis by Annexin V Staining - Apoptotic cells were quantified by measuring externalized phosphatidylserine (PS) assessed by Annexin-V-APC labelling and propidium iodide uptake as described previously (Sun et al., 1999) and were analyzed on a FACSCalibur flow cytometer using CellQuest software (BD Biosciences, San Jose, CA).

Western Blot Analysis - SDS-PAGE and Western immunoblotting were carried out as described previously (MacFarlane et al., 1997).

In vitro GST pull-downs - Jurkat cell lysates were made essentially as described (Harper et al., 2003; Hughes et al., 2013). For receptor pull-downs, 10 mg of cell lysate (20 mg/ml) was incubated with 10 μg of purified GST-TRAIL-R1-IcD fusions bound to glutathione-Sepharose beads for 16 h at 23°C. Where indicated, caspase-8-deficient lysates were supplemented with the specified amounts of IVT generated r-procaspase-8b in the presence of zVAD.fmk (5 μM). Bead-associated complexes were washed four times in PBS containing protease inhibitors, released from beads by boiling for 5 min in SDS sample buffer, and analysed by SDS-PAGE/Western blotting.

Pre-assembly of r-DISC complexes - The r-DISC was initially reconstituted in caspase assay buffer using GST-TRAIL-R1-IcD (10 μg), r-FADD (5 μg) and ^{35}S-labeled r-Casp-8b (160 μl) for 16 h at either 16°C or 20°C. Control reconstitutions contained beads coated with GST alone. The resulting bead complexes were washed four times in PBS (the corresponding supernatant post 1st bind was retained). A second bind was performed by incubating c-FLIP$_{L/S}$ (0 - 150 μl) with the pre-assembled r-DISC in caspase assay buffer for 16 h at either 16°C or 20°C. Bead-associated complexes were washed four times in PBS (the corresponding supernatant post 2nd bind was retained), released from beads by boiling for 5 min in SDS sample buffer, and analysed by SDS-PAGE/Western blotting.

LC-MS/MS identification and quantification of DISC proteins - TRAIL and CD95 DISC proteins were analysed by LC-MS/MS. Briefly, purified protein complexes were separated on SDS-PAGE gels, stained with colloidal coomassie, serially sectioned and the gel slices destained before reduction/alkylation of the proteins and digestion with trypsin (Boyd et al., 2009; Dickens et al., 2012). Extracted tryptic peptides were concentrated to dryness and resuspended in 5% formic acid (FA) and acetonitrile (9:1), spiked with 20-40 fmol/μl ADH1 and BSA MassPREP standards (Waters Corporation, Manchester, UK). Aliquots (2-4 μl) were applied to a reverse phase BEH130 C18 column (25 cm X 75 μm X 1.7 μm I.D.) using a Waters nanoAcquity UPLC system interfaced to a Synapt G2-S HDMS mass

spectrometer. Peptides were eluted (0.3 µl/min) with 50 min, 3-40 % (0.1% FA/acetonitrile) gradients and analysed in data-independent acquisition (DIA) and ion mobility ($HDMS^E$) modes using a T-wave velocity of 650 m/sec (Craxton et al., 2015). Stepped 4 eV and 20-50 eV voltage switching generated collision induced (CID) peptide fragmentation. Low energy and CID LC-MS/MS data were acquired (1 sec cycle scan time and 50-2000 *m/z* mass range) and processed using Waters ProteinLynx Global SERVER (PLGS 3.0) using the UniProt Human database (UniProtKB release 2014_11, 20,265 entries). Peptide mass and fragment mass tolerances were set to auto, with one missed cleavage and variable modifications for methionine oxidation and carbamidomethylation of cysteines. The PLGS "TOP 3" method with a false discovery rate of 1% was used for absolute protein quantification as described previously (Silva et al., 2006a; Silva et al., 2006b).

Supplemental References

Boyd, R.S., Jukes-Jones, R., Walewska, R., Brown, D., Dyer, M.J., and Cain, K. (2009). Protein profiling of plasma membranes defines aberrant signaling pathways in mantle cell lymphoma. Mol Cell Proteomics *8*, 1501-1515.

Craxton, A., Somers, J., Munnur, D., Jukes-Jones, R., Cain, K., and Malewicz, M. (2015). XLS (c9orf142) is a new component of mammalian DNA double-stranded break repair. Cell Death Differ *22*, 890-897.

Dickens, L.S., Boyd, R.S., Jukes-Jones, R., Hughes, M.A., Robinson, G.L., Fairall, L., Schwabe, J.W.R., Cain, K., and MacFarlane, M. (2012). A Death Effector Domain Chain DISC Model Reveals a Crucial Role for Caspase-8 Chain Assembly in Mediating Apoptotic Cell Death. Mol Cell *47*, 291-305.

Geserick, P., Drewniok, C., Hupe, M., Haas, T.L., Diessenbacher, P., Sprick, M.R., Schon, M.P., Henkler, F., Gollnick, H., Walczak, H.*, et al.* (2008). Suppression of cFLIP is sufficient to sensitize human melanoma cells to TRAIL- and CD95L-mediated apoptosis. Oncogene *27*, 3211-3220.

Geserick, P., Hupe, M., Moulin, M., Wong, W.W., Feoktistova, M., Kellert, B., Gollnick, H., Silke, J., and Leverkus, M. (2009). Cellular IAPs inhibit a cryptic CD95-induced cell death by limiting RIP1 kinase recruitment. J Cell Biol *187*, 1037-1054.

Harper, N., Hughes, M., MacFarlane, M., and Cohen, G.M. (2003). Fas-associated death domain protein and caspase-8 are not recruited to the tumor necrosis factor receptor 1 signaling complex during tumor necrosis factor-induced apoptosis. J Biol Chem *278*, 25534-25541.

Hughes, M.A., Harper, N., Butterworth, M., Cain, K., Cohen, G.M., and MacFarlane, M. (2009). Reconstitution of the death-inducing signaling complex reveals a substrate switch that determines CD95-mediated death or survival. Mol Cell *35*, 265-279.

Hughes, M.A., Langlais, C., Cain, K., and MacFarlane, M. (2013). Isolation, characterisation and reconstitution of cell death signalling complexes. Methods *61*, 98-104.

MacFarlane, M., Cain, K., Sun, X.M., Alnemri, E.S., and Cohen, G.M. (1997). Processing/activation of at least four interleukin-1beta converting enzyme-like proteases occurs during the execution phase of apoptosis in human monocytic tumor cells. J Cell Biol *137*, 469-479.

Silva, J.C., Denny, R., Dorschel, C., Gorenstein, M.V., Li, G.Z., Richardson, K., Wall, D., and Geromanos, S.J. (2006a). Simultaneous qualitative and quantitative analysis of the Escherichia coli proteome: a sweet tale. Mol Cell Proteomics *5*, 589-607.

Silva, J.C., Gorenstein, M.V., Li, G.Z., Vissers, J.P.C., and Geromanos, S.J. (2006b). Absolute quantification of proteins by LCMSE - A virtue of parallel MS acquisition. Mol Cell Proteomics *5*, 144-156.

Sun, X.M., MacFarlane, M., Zhuang, J., Wolf, B.B., Green, D.R., and Cohen, G.M. (1999). Distinct caspase cascades are initiated in receptor-mediated and chemical-induced apoptosis. J Biol Chem *274*, 5053-5060.
