# Supplementary material for: Co-operative and Hierarchical Binding of c-FLIP and Caspase-8: A Unified Model Defines How c-FLIP Isoforms Differentially Control Cell Fate
Source: Mol Cell. 2016 Mar 17;61(6):834–49. doi: 10.1016/j.molcel.2016.02.023 (PMC4819448; doi:10.1016/j.molcel.2016.02.023)
Supplement: Document S2. Article plus Supplemental Information [file mmc2.pdf]

Article

Molecular Cell

Co-operative and Hierarchical Binding of c-FLIP and Caspase-8: A Unified Model Defines How c-FLIP Isoforms Differentially Control Cell Fate

Graphical Abstract

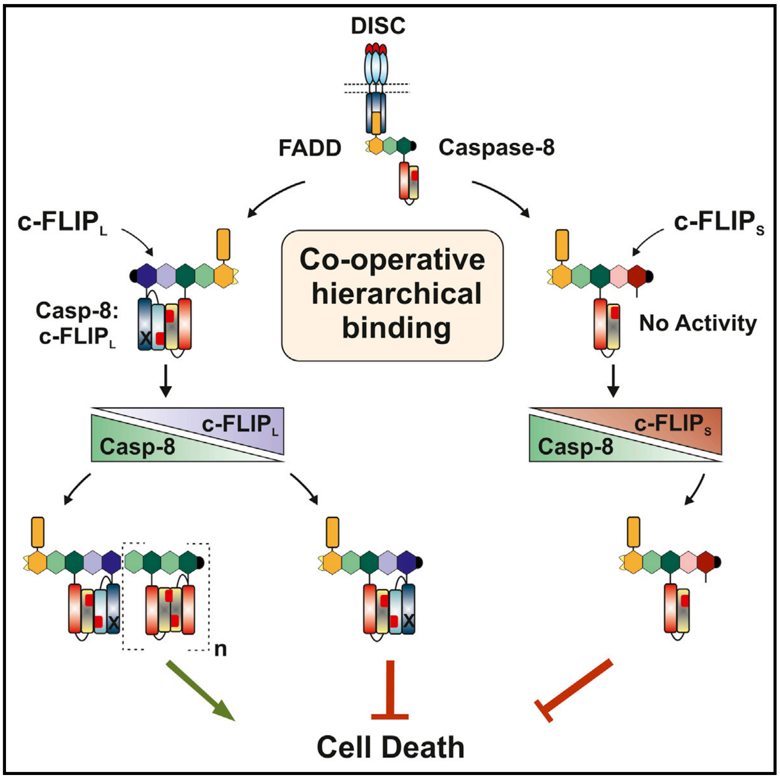

Highlights

- c-FLIP isoforms (L/S) do not directly compete with caspase-8 for binding to FADD
- c-FLIP binds to the DISC via a co-operative hierarchical caspase-8-dependent process
- Co-operative and hierarchical binding crucially explains the dual function of c-FLIP$_L$
- Our unified model defines how c-FLIP isoforms differentially direct cell fate

Authors

Michelle A. Hughes, Ian R. Powley, Rebekah Jukes-Jones, ..., Martin Leverkus, Kelvin Cain, Marion MacFarlane

Correspondence

kc5@le.ac.uk (K.C.), mm21@le.ac.uk (M.M.)

In Brief

It is unclear how c-FLIP isoforms can differentially regulate caspase-8 activation to direct cell fate. Hughes et al. show that c-FLIP$_{L/S}$ recruitment to FADD is indirect and requires caspase-8. This co-operative hierarchical binding process explains the conundrum of the dual role of c-FLIP$_L$ and defines how c-FLIP$_{L/S}$ differentially control cell fate.

Hughes et al., 2016, Molecular Cell *61*, 834–849
March 17, 2016 ©2016 The Authors
http://dx.doi.org/10.1016/j.molcel.2016.02.023

Cell Press

Molecular Cell
Article

Co-operative and Hierarchical Binding of c-FLIP and Caspase-8: A Unified Model Defines How c-FLIP Isoforms Differentially Control Cell Fate

Michelle A. Hughes,[1] Ian R. Powley,[1] Rebekah Jukes-Jones,[1] Sebastian Horn,[3] Maria Feoktistova,[4] Louise Fairall,[2] John W.R. Schwabe,[2] Martin Leverkus,[4] Kelvin Cain,[1,*] and Marion MacFarlane[1,*]

[1]MRC Toxicology Unit, Hodgkin Building, P.O. Box 138, Lancaster Road, Leicester LE1 9HN, UK

[2]Henry Wellcome Laboratories of Structural Biology, Department of Molecular and Cell Biology, University of Leicester, Lancaster Road, Leicester LE1 9HN, UK

[3]Department of Dermatology, Venereology and Allergology, Medical Faculty Mannheim, University of Heidelberg, Theodor-Kutzer-Ufer 1-3, 68167 Mannheim, Germany

[4]Department of Dermatology and Allergology, Medical Faculty of the RWTH Aachen, Pauwelsstraße 30, 52074 Aachen, Germany

*Correspondence: kc5@le.ac.uk (K.C.), mm21@le.ac.uk (M.M.)

http://dx.doi.org/10.1016/j.molcel.2016.02.023

This is an open access article under the CC BY license (http://creativecommons.org/licenses/by/4.0/).

SUMMARY

The death-inducing signaling complex (DISC) initiates death receptor-induced apoptosis. DISC assembly and activation are controlled by c-FLIP isoforms, which function as pro-apoptotic (c-FLIP$_L$ only) or anti-apoptotic (c-FLIP$_L$/c-FLIP$_S$) regulators of procaspase-8 activation. Current models assume that c-FLIP directly competes with procaspase-8 for recruitment to FADD. Using a functional reconstituted DISC, structure-guided mutagenesis, and quantitative LC-MS/MS, we show that c-FLIP$_{L/S}$ binding to the DISC is instead a co-operative procaspase-8-dependent process. FADD initially recruits procaspase-8, which in turn recruits and heterodimerizes with c-FLIP$_{L/S}$ via a hierarchical binding mechanism. Procaspase-8 activation is regulated by the ratio of unbound c-FLIP$_{L/S}$ to procaspase-8, which determines composition of the procaspase-8:c-FLIP$_{L/S}$ heterodimer. Thus, procaspase-8:c-FLIP$_L$ exhibits localized enzymatic activity and is preferentially an activator, promoting DED-mediated procaspase-8 oligomer assembly, whereas procaspase-8:c-FLIP$_S$ lacks activity and potently blocks procaspase-8 activation. This co-operative hierarchical binding model explains the dual role of c-FLIP$_L$ and crucially defines how c-FLIP isoforms differentially control cell fate.

INTRODUCTION

Apoptotic cell death, which plays a fundamental role during development and homeostasis of multicellular organisms, is orchestrated by the caspase family of cysteine proteases. Deregulated apoptosis is a hallmark of several diseases, including autoimmunity, neurodegeneration, and cancer. The extrinsic apoptotic pathway is initiated by "death ligand"-induced ligation of death receptors (DR), such as CD95 (Fas/Apo1), TRAIL (TNF-related apoptosis-inducing ligand) receptors-1/-2, (TRAIL-R1/R2) and tumor necrosis factor (TNF) receptor-1 (TNF-R1), which form part of the TNFR superfamily (Dickens et al., 2012b). Stimulation of CD95 or TRAIL-R1/R2 by their cognate ligands or agonistic antibodies triggers formation of a multiprotein death-inducing signaling complex (DISC), comprising receptors, the bipartite adaptor molecule FADD (Fas-associated death domain protein), the initiator caspases-8 and -10, and the catalytically inactive caspase-8 homolog, c-FLIP (Kischkel et al., 1995). FADD is recruited to DR through direct interactions of the death domains (DD) present on both proteins; this exposes the FADD death effector domain (DED) (Esposito et al., 2010; Scott et al., 2009; Wang et al., 2010), promoting recruitment of DED-only proteins including procaspase-8. Once recruited to FADD, multiple procaspase-8 molecules interact via their tandem DEDs forming a DED chain-based procaspase-8 activation platform (Dickens et al., 2012a; Schleich et al., 2012), thereby facilitating both proximity-induced dimerization and proteolytic cleavage of procaspase-8, which are required for initiation of apoptotic cell death (Hughes et al., 2009; Oberst et al., 2010).

In addition to its key role in apoptosis, caspase-8 has a survival role as it is required for embryonic development (Dillon et al., 2012; Varfolomeev et al., 1998), immune cell proliferation (Salmena et al., 2003), and resistance to RIPK1-RIPK3-mediated programmed necrosis (Kaiser et al., 2011; Oberst et al., 2011). In all of these roles, c-FLIP is a key regulator that determines the activity of caspase-8 (Dillon et al., 2012; Hinshaw-Makepeace et al., 2008; Koenig et al., 2014; Oberst et al., 2011). Although c-FLIP has multiple splice forms at the mRNA level, two major protein isoforms predominate, namely c-FLIP long (c-FLIP$_L$) and c-FLIP short (c-FLIP$_S$) (Irmler et al., 1997; Scaffidi et al., 1999). c-FLIP$_S$ is a truncated version of procaspase-8 containing tandem DEDs only, whereas c-FLIP$_L$ closely resembles full-length procaspase-8 but critically lacks the active site catalytic cysteine residue and proteolytic activity. c-FLIP$_S$ inhibits DR-mediated apoptosis by blocking caspase-8 activation at the DISC (Krueger et al., 2001b; Scaffidi et al., 1999). While c-FLIP$_S$ appears to act purely as an antagonist of caspase-8

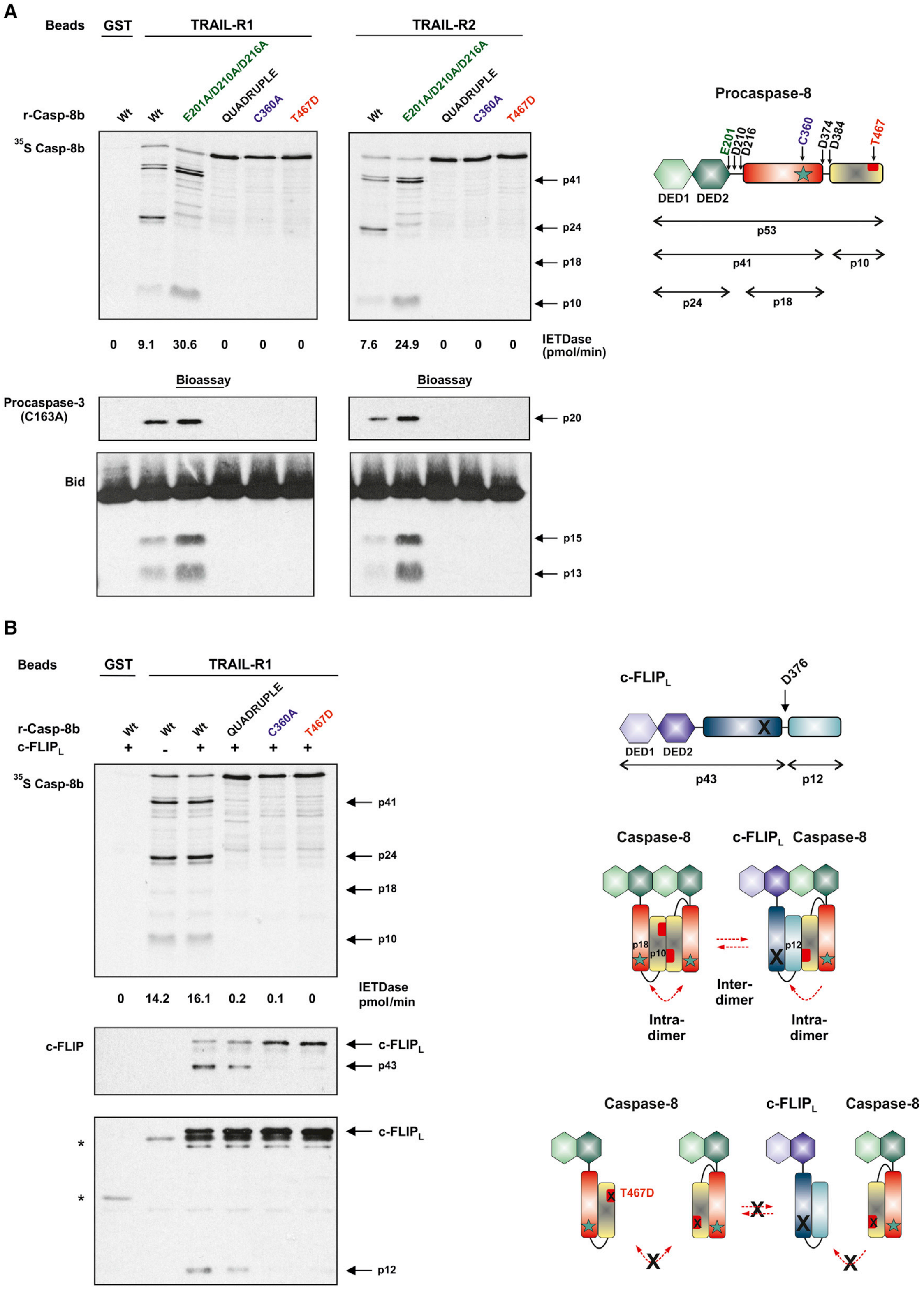

Figure 1. Procaspase-8 Dimerization Is Required for Procaspase-8 Cleavage of c-FLIP$_L$ at the DISC

(A) Domain structure of procaspase-8b showing cleavage sites, active site (C360), and dimerization residue (T467) (Machα1 numbering). Reconstituted TRAIL DISC was assembled using GST-TRAIL-R1/R2 intracellular domain (TRAIL-R1/R2-IcD), recombinant FADD (r-FADD), and ^{35}S-labeled recombinant

(legend continued on next page)

activity, c-FLIP$_L$ has a more controversial role, being variously reported as either an activator or inhibitor of procaspase-8 (reviewed in Thome and Tschopp, 2001; Oztürk et al., 2012). Hence, during both development and immune cell proliferation, c-FLIP$_L$:procaspase-8 heterodimers function to inhibit RIPK1-RIPK3-mediated programmed necrosis (Oberst et al., 2011). Likewise, the Ripoptosome, which is formed upon genotoxic stress or loss of inhibitor-of-apoptosis proteins (IAPs), is regulated by c-FLIP$_{L/S}$:procaspase-8 heterodimers (Feoktistova et al., 2011, 2012; Tenev et al., 2011). Thus, in a variety of signaling complexes, regulation of caspase-8 by c-FLIP isoforms is a critical step in determining signaling outcome resulting in cell survival or diverse modes of cell death.

The key question is how does c-FLIP modulate procaspase-8 activation/activity to produce diverse signaling outcomes? Current models propose that c-FLIP competes directly with procaspase-8 for binding to FADD through homotypic DED interactions, thus inhibiting procaspase-8 recruitment and activation at the DISC (Irmler et al., 1997; Rasper et al., 1998; Yang et al., 2005). Thus, both c-FLIP$_S$ and overexpressed c-FLIP$_L$ can block DISC-dependent procaspase-8 activation (Fricker et al., 2010; Kavuri et al., 2011; Krueger et al., 2001b; Scaffidi et al., 1999). However, lower physiological levels of c-FLIP$_L$ enhance procaspase-8 activity as c-FLIP$_L$ forms a highly active heterodimeric complex with procaspase-8 (Boatright et al., 2004), resulting in increased apoptosis (Chang et al., 2002; Fricker et al., 2010; Micheau et al., 2002; Scaffidi et al., 1999). Although difficult to explore biochemically, studies using truncated c-FLIP$_L$ and caspase-8 proteins lacking their DED-containing prodomains have revealed that heterodimerization of c-FLIP$_L$ with procaspase-8 leads to rearrangement of the catalytic site of procaspase-8, producing an active conformation (Chang et al., 2002; Micheau et al., 2002; Yu et al., 2009). Similarly, in vitro and structural studies show that caspase-8 has a higher affinity for c-FLIP$_L$ than for itself, suggesting that procaspase-8:c-FLIP$_L$ heterodimers may be preferred over procaspase-8 homodimers. Furthermore, c-FLIP$_L$ can heterodimerize with and activate non-cleavable procaspase-8 without autoprocessing, resulting in a limited/selective substrate specificity (Boatright et al., 2004; Pop et al., 2011; Yu et al., 2009). Thus, it is possible that procaspase-8:c-FLIP$_L$ heterodimers can potentially initiate different signaling outcomes. However, the mechanism of c-FLIP$_{L/S}$ recruitment into these FADD-containing signaling complexes and how this controls procaspase-8 recruitment/activation to effect and direct alternate signaling outcomes has not been delineated.

We now reveal a hitherto unknown mechanism that explains how c-FLIP$_{L/S}$ are recruited to the DISC and differentially regulate caspase-8 activation to control cell fate. An in vitro DISC reconstitution model utilizing structure-guided DED mutants of full-length FADD, procaspase-8, and c-FLIP$_{L/S}$ has revealed that, contrary to current thinking, c-FLIP isoforms do not directly compete with procaspase-8 for binding to FADD. Surprisingly, optimal c-FLIP recruitment to the DISC is a co-operative and hierarchical process in which procaspase-8 binding to FADD is the primary initiating event, which in turn promotes recruitment of c-FLIP via heterodimerization with procaspase-8. Thus, we have identified a regulatory mechanism involving procaspase-8:c-FLIP$_{L/S}$ heterodimers where procaspase-8 activation is determined by composition of the heterodimer. Consequently, procaspase-8:c-FLIP$_L$, which exhibits local heterodimer-dependent enzymatic activity, functions primarily as a DISC activator by promoting DED-mediated recruitment of multiple procaspase-8 molecules. In contrast, procaspase-8:c-FLIP$_S$ lacks enzymatic activity and potently blocks procaspase-8 activation. Moreover, using quantitative mass spectrometry and confocal imaging, we show that high levels of c-FLIP$_S$ (or c-FLIP$_L$) inhibit DISC activation by blocking formation of DED-mediated procaspase-8 oligomers. Significantly, this alternative mechanism for c-FLIP$_{L/S}$ recruitment to the DISC and regulation of DED:DED interactions critically determines procaspase-8 function. We now propose a co-operative and hierarchical binding model that explains the conundrum of the dual functionality of c-FLIP$_L$ and crucially defines how c-FLIP isoforms differentially control cell fate. This unified model represents a paradigm shift in terms of our understanding of how c-FLIP regulates the DISC as well as other caspase-8-dependent signaling platforms involved in development, physiology, and disease.

RESULTS

Procaspase-8 Dimerization, but Not Cleavage, Is Essential for DISC-Associated c-FLIP$_L$ Processing

In this study, we have reconstituted a fully functional TRAIL DISC using only GST-tagged TRAIL-R1/R2 intracellular domain (TRAIL-R1/R2-IcD) bound to glutathione beads, full-length recombinant FADD (r-FADD), and procaspase-8b (procaspase-8). In this model system, procaspase-8 cleavage to its signature fragments and IETDase activity were only detected in the presence of TRAIL-R1/R2-IcD, r-FADD, and ^{35}S-labeled procaspase-8 (Figures 1A and S1A). Significantly, introducing an *lpr*-like mutation into TRAIL-R1/R2-IcD, which inhibits CD95 DD-mediated FADD recruitment and receptor signaling, or DISC reconstitution with TRAIL-R4-IcD, which has a truncated intracellular domain and cannot signal for apoptosis, abolished FADD recruitment, procaspase-8 binding, and IETDase activity (Figure S1B). These results demonstrate that the reconstituted TRAIL-R1/R2 DISC fully recapitulates the functional assembly and proteolytic activity of a native DISC.

We then investigated the mechanism of procaspase-8 activation in the TRAIL-R1/R2 DISC using a cleavage bioassay for exogenous DISC substrates essential for receptor-mediated

procaspase-8b (^{35}S r-Casp-8b, 100 μl) wild-type (WT), highly active (E201A/D210A/D216A), non-cleavable Quadruple (D210A/D216A/D374A/D384A), active site (C360A), or dimerization (T467D) mutants. TRAIL-R1/R2 DISCs were analyzed for r-Casp-8b cleavage fragments, IETDase activity, and proteolytic cleavage of procaspase-3 (C163A) or Bid. See also Figure S1.

(B) TRAIL-R1 DISC (r-DISC) assembled using indicated ^{35}S r-Casp-8b variants (100 μl) alone (WT only) or in combination with c-FLIP$_L$ (25 μl). Beads were assessed for r-Casp-8b cleavage, c-FLIP$_L$ cleavage, and IETDase activity. Domain structure of c-FLIP$_L$ showing caspase-8 cleavage site (D376). Scheme shows intra-dimer/inter-dimer cleavage of c-FLIP$_L$ when combined with r-Casp-8b WT or dimerization mutant (T467D). *non-specific band.

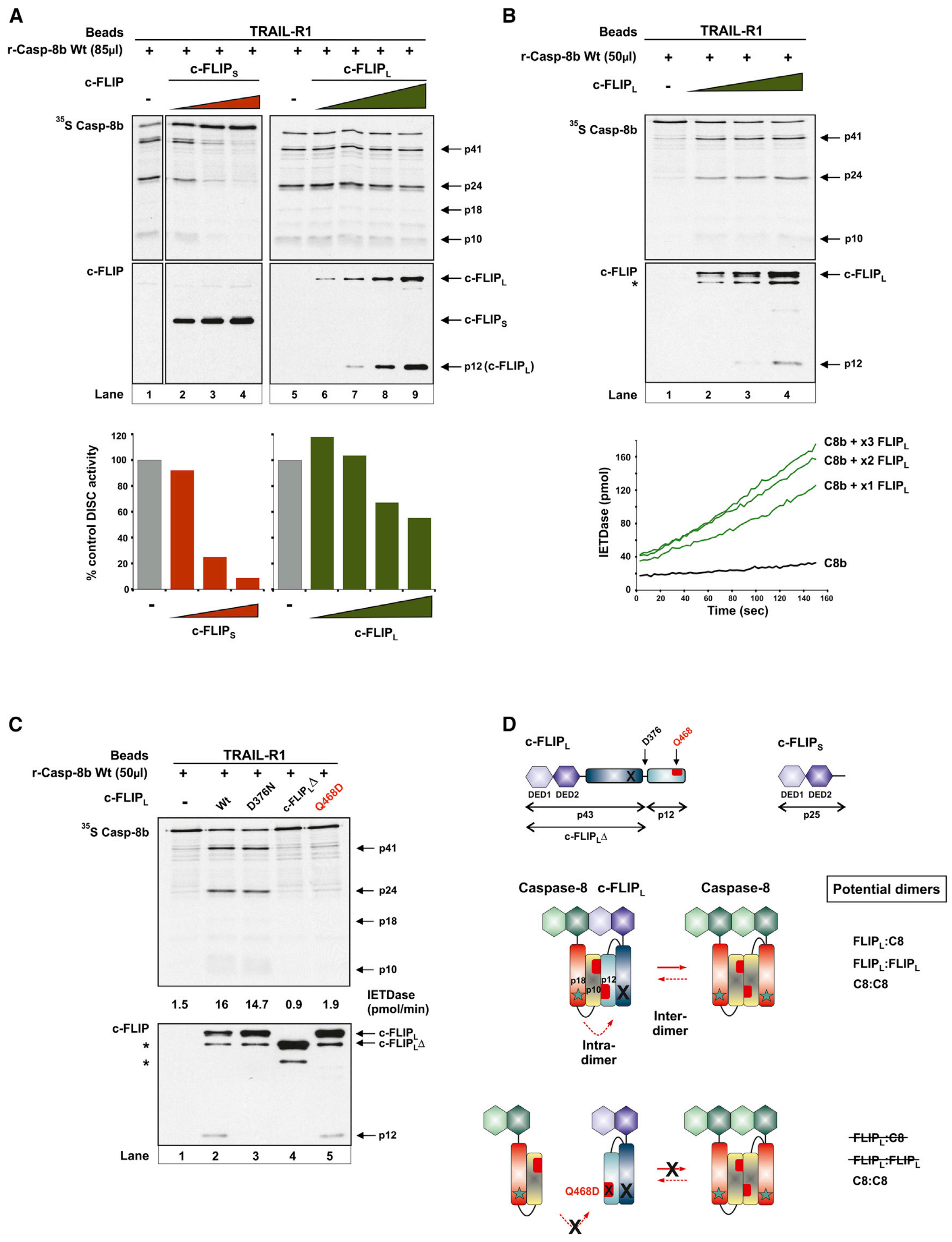

Figure 2. c-FLIP$_L$/Procaspase-8 Heterodimerization Is Critical for c-FLIP-Mediated Procaspase-8 Activation

(A) r-DISCs assembled with ^{35}S r-Casp-8b (85 μl) alone (–) or with increasing amounts of c-FLIP$_S$ (0–75 μl) or c-FLIP$_L$ (0–225 μl). Beads were analyzed for r-Casp-8b cleavage, c-FLIP$_{L/S}$, and IETDase activity; control (–) r-DISC activity expressed as 100%.

(B) r-DISC reconstituted (16 hr at 16°C) using sub-optimal levels of ^{35}S r-Casp-8b (50 μl) with increasing amounts of c-FLIP$_L$ (0–75 μl). Beads were analyzed as in (A); graph shows time-dependent IETD.AFC hydrolysis.

(legend continued on next page)

apoptosis, namely procaspase-3 and Bid. Both procaspase-3 (C163A) and Bid were efficiently cleaved by a DISC reconstituted with wild-type (WT) procaspase-8 (Figure 1A). Moreover, the E201A/D210A/D216A procaspase-8 mutant, which only undergoes initial autocatalytic cleavage at D374/D384 generating a highly active p41/p10 form of caspase-8, further enhanced DISC-associated IETDase activity and procaspase-3/Bid cleavage. In marked contrast, although non-cleavable (Quadruple), active-site (C360A), or dimerization (T467D) mutants of procaspase-8 were efficiently recruited to the TRAIL-R1/R2 DISC, they did not support IETDase activity or cleavage of procaspase-3/Bid (Figure 1A). Thus, both dimerization and cleavage of procaspase-8 are absolutely required to achieve a fully functional TRAIL-R1/R2 DISC, further supporting the two-step procaspase-8 activation mechanism initially proposed for the CD95 DISC (Hughes et al., 2009).

As well as being a regulator of DISC function, c-FLIP$_L$ is also a substrate for active caspase-8, which cleaves c-FLIP$_L$ at D376, generating a DED containing p43 fragment and a C-terminal p12 fragment (Figure 1B) (Krueger et al., 2001b; Micheau et al., 2002; Scaffidi et al., 1999; Shu et al., 1997). To elucidate the mechanism of procaspase-8-mediated cleavage of c-FLIP$_L$, we assessed whether various caspase-8 mutants could cleave c-FLIP$_L$ within a reconstituted TRAIL-R1 DISC (r-DISC). c-FLIP$_L$ was cleaved to its signature p43/p12 fragments by both activated WT and non-cleavable (Quadruple) procaspase-8, but not by an active site (C360A) or dimerization (T467D) mutant (Figure 1B), demonstrating that procaspase-8 dimerization is essential for processing adjacent c-FLIP$_L$ molecules. Procaspase-8 can also form heterodimers with c-FLIP$_L$, facilitating intra-dimer/inter-dimer -mediated c-FLIP$_L$ cleavage (Chang et al., 2002). Thus, procaspase-8/c-FLIP$_L$ heterodimerization and/or procaspase-8 homodimerization (Figure 1B, scheme), but not procaspase-8 cleavage, is crucial for c-FLIP$_L$ processing within the DISC.

c-FLIP$_L$/Procaspase-8 Heterodimerization Is Essential for Concentration-Dependent c-FLIP$_L$ Activation of Procaspase-8

c-FLIP is a key regulator of procaspase-8 in several cell death signaling platforms including the DISC (Fricker et al., 2010; Krueger et al., 2001a; Öztürk et al., 2012), the necrosome (Dillon et al., 2012; Oberst et al., 2011; Vanlangenakker et al., 2012), and ripoptosome (Feoktistova et al., 2011, 2012; Tenev et al., 2011). At high levels of ectopic expression, c-FLIP$_L$ and c-FLIP$_S$ function as anti-apoptotic regulators (Irmler et al., 1997; Kavuri et al., 2011; Krueger et al., 2001a; Scaffidi et al., 1999). However, c-FLIP$_L$ can also function as a critical activator of procaspase-8, amplifying DISC-mediated apoptosis (Chang et al., 2002; Micheau et al., 2002; Shu et al., 1997). To solve this apparent conundrum and to investigate how c-FLIP isoforms can differentially regulate procaspase-8, we investigated how c-FLIP$_L$ or c-FLIP$_S$ modulate procaspase-8 activation in the r-DISC (Figure 2A).

First, we showed that r-DISC assembly with increasing amounts of c-FLIP$_S$ (Figure 2A, lanes 1–4) resulted in concentration-dependent inhibition of procaspase-8 cleavage/IETDase activity; thus, c-FLIP$_S$ functions exclusively as an inhibitor of procaspase-8 activation. In contrast, c-FLIP$_L$ exhibited a biphasic effect on r-DISC-mediated activation of procaspase-8 (Figure 2A, lanes 5–9), with low concentrations of c-FLIP$_L$ promoting procaspase-8 activation, whereas higher levels of c-FLIP$_L$ resulted in concentration-dependent inhibition of procaspase-8 activity. Thus, the c-FLIP$_L$:procaspase-8 ratio within the DISC critically determines procaspase-8 activation and signaling outcome. We further characterized the ability of low levels of c-FLIP$_L$ to enhance DISC-mediated procaspase-8 activation and apoptosis by reconstituting the r-DISC using sub-optimal levels of procaspase-8. Sub-optimal amounts of procaspase-8 displayed negligible proteolytic cleavage or activity against IETD.AFC (Figure 2B, lane 1). However, co-incubation with increasing amounts of c-FLIP$_L$ produced a concentration-dependent activation of procaspase-8, as shown by autocatalytic cleavage of procaspase-8, its substrate c-FLIP$_L$, and increased r-DISC-associated IETDase activity (Figure 2B, lanes 2–4; lower graph). Thus, high levels of c-FLIP$_L$ inhibit DISC-mediated procaspase-8 activation, whereas at physiological concentrations (Chang et al., 2002), c-FLIP$_L$ activates DISC-bound procaspase-8.

To characterize how c-FLIP$_L$ activates procaspase-8, we assessed the impact of various c-FLIP$_L$ mutants on r-DISC-mediated procaspase-8 activation (Figure 2C). The non-cleavable c-FLIP$_L$ mutant, D376N, fully activated procaspase-8, resulting in IETDase activity comparable to WT c-FLIP$_L$ (Figure 2C, lanes 1–3). In contrast, a c-FLIP$_L$ mutant lacking the C-terminal p12 domain (c-FLIP$_L\Delta$) did not activate procaspase-8 (Figure 2C, lane 4). In agreement with a key role for c-FLIP$_L$ p12 domain, a mutant defective in the predicted heterodimer interface of c-FLIP$_L$ (Q468D) (Micheau et al., 2002; Yu et al., 2009) completely abrogated c-FLIP$_L$-mediated activation of procaspase-8 (Figure 2C, lane 5). Thus, c-FLIP$_L$/procaspase-8 heterodimerization, without c-FLIP$_L$ cleavage, is essential for c-FLIP$_L$-mediated procaspase-8 activation within the DISC (Figure 2D, scheme).

DISC Reconstitution Reveals Co-operative Recruitment of c-FLIP and Procaspase-8

Current models propose that c-FLIP inhibits apoptosis by competitively inhibiting procaspase-8 recruitment and activation within the DISC (Irmler et al., 1997; Rasper et al., 1998; Yang et al., 2005). We investigated this hypothesis further using the DISC reconstitution model to determine whether c-FLIP$_{L/S}$ competes with procaspase-8 for binding to FADD. To assess binding of procaspase-8 and c-FLIP$_{L/S}$, we reconstituted the r-DISC using excess levels of catalytically inactive (C360A) procaspase-8.

(C) r-DISCs assembled (16 hr at 16°C) with ^{35}S r-Casp-8b (50 μl) alone (–) or with c-FLIP$_L$ mutants (50 μl) shown in (D). Beads were analyzed for r-Casp-8b cleavage, c-FLIP$_L$, and IETDase activity.

(D) Domain organization of c-FLIP$_{L/S}$ showing c-FLIP$_L$ caspase-8 cleavage site (D376), dimerization residue (Q468), and p43 fragment (FLIP$_L$ Δ). Scheme shows intra-dimer/inter-dimer activation of r-Casp-8b WT when combined with c-FLIP$_L$ WT or dimerization mutant (Q468D). *non-specific band.

CellPress

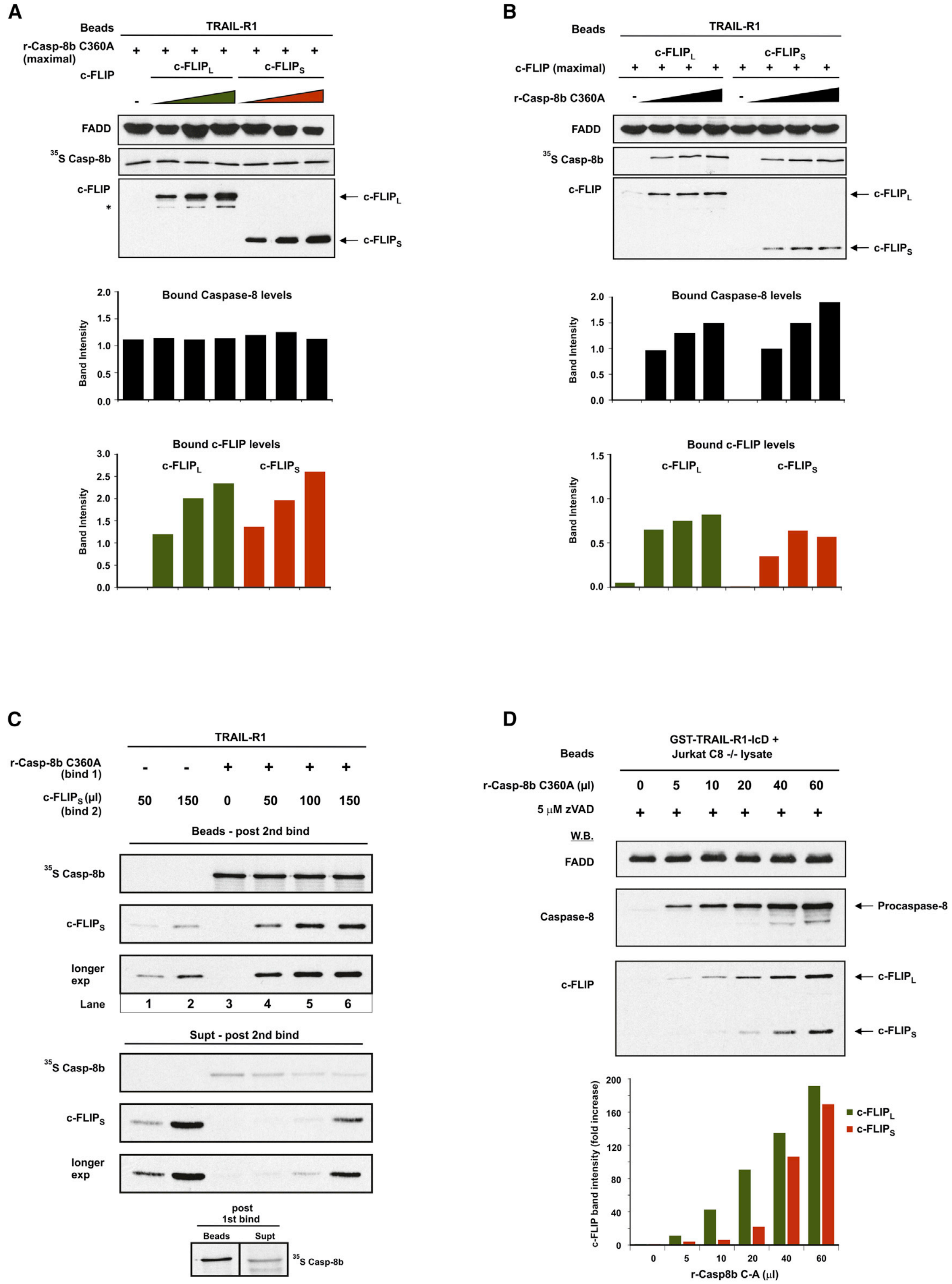

Figure 3. DISC Reconstitution Reveals Co-operative and Hierarchical Recruitment of c-FLIP$_{L/S}$ and Procaspase-8

(A) r-DISCs assembled using ^{35}S r-Casp-8b C360A (100 µl) with increasing amounts of c-FLIP$_{L/S}$ (0–75 µl). Beads were analyzed for FADD, r-Casp-8b, and c-FLIP$_{L/S}$ binding and quantified by densitometry (bar graphs).

(B) r-DISCs reconstituted with c-FLIP$_{L/S}$ (85 µl) and increasing amounts of ^{35}S r-Casp-8b C360A (0–75 µl) were analyzed as in (A).

(legend continued on next page)

Strikingly, co-incubation with increasing amounts of c-FLIP$_{L/S}$ did not affect procaspase-8 recruitment to FADD. Indeed, despite the presence of procaspase-8, c-FLIP$_{L/S}$ recruitment increased in a concentration-dependent manner (Figure 3A). Moreover, incubating excess amounts of c-FLIP$_{L/S}$ with a pre-assembled r-DISC, containing maximal levels of procaspase-8 (C360A), failed to displace procaspase-8 from FADD, and both c-FLIP$_L$ and c-FLIP$_S$ were recruited to the pre-assembled complex (Figure S2A). Significantly, these data indicate that, contrary to current dogma, c-FLIP$_{L/S}$ do not directly compete with procaspase-8 for binding to FADD. Instead, DISC recruitment of c-FLIP occurs via a mechanism that is different from procaspase-8.

To explore this further, the r-DISC was reconstituted with excess c-FLIP$_{L/S}$ and increasing amounts of procaspase-8 (C360A). Remarkably, without procaspase-8, there was negligible recruitment of c-FLIP$_{L/S}$ to FADD. However, increasing amounts of procaspase-8 resulted in concentration-dependent recruitment of both c-FLIP$_{L/S}$ and procaspase-8 (Figure 3B), concomitant with decreasing amounts of unbound c-FLIP remaining in the supernatant (Figure S2B). Thus, r-DISC (TRAIL-R1 or CD95) recruitment of c-FLIP$_{L/S}$ to FADD is significantly enhanced when procaspase-8 is present (Figures 3B and S2C). This conclusion was confirmed by adding increasing amounts of c-FLIP$_S$ to a pre-assembled r-DISC, which, without procaspase-8 (C360A), recruited only minimal amounts of c-FLIP$_S$ (Figure 3C). Conversely, when the r-DISC was pre-assembled with procaspase-8, c-FLIP$_S$ recruitment was greatly enhanced (Figure 3C, compare lanes 1 and 2 with lanes 4–6). Intriguingly, it appears that only a finite amount of c-FLIP$_S$ can be recruited to a fully pre-assembled r-DISC since addition of more c-FLIP$_S$ did not further increase c-FLIP$_S$ recruitment to the complex (Figure 3C, lanes 5 and 6). Crucially, our data reveal that c-FLIP does not displace procaspase-8 from FADD; instead, optimal c-FLIP recruitment to the DISC is critically dependent on procaspase-8.

To confirm that procaspase-8 is required for c-FLIP recruitment to the DISC, we extended our findings in a cellular context using caspase-8-deficient Jurkat T cells. TRAIL-R1 DISC formation was assessed in caspase-8-deficient cell lysates, supplemented with increasing amounts of recombinant procaspase-8 (C360A) (Figure 3D). Strikingly, despite efficient recruitment of FADD to TRAIL-R1-IcD, in the absence of procaspase-8 no endogenous c-FLIP$_{L/S}$ was recruited to the DISC. However, addition of increasing amounts of exogenous procaspase-8 resulted in a concentration-dependent increase in recruitment of both c-FLIP$_L$/c-FLIP$_S$ to the DISC, concomitant with a decrease in c-FLIP$_{L/S}$ in cell lysates (Figures 3D and S2D). Thus, c- FLIP$_{L/S}$ do not compete with procaspase-8 for binding to FADD; instead, c-FLIP recruitment to the DISC requires procaspase-8. This provides strong evidence for an alternative model of DISC assembly involving co-operative recruitment of c-FLIP and procaspase-8.

Mutation of Procaspase-8 DED1 Pocket Prevents Caspase-8 Recruitment to the DISC

To investigate the co-operative DISC recruitment of c-FLIP and procaspase-8, we first needed to characterize how procaspase-8 is recruited to FADD. A critical phenylalanine/leucine (F122/L123) hydrophobic motif in procaspase-8 DED2 has previously been shown to occupy a hydrophobic pocket in DED1 of an adjacent procaspase-8 molecule (Dickens et al., 2012a) and results in the formation of a DED chain-based procaspase-8 activation platform at the DISC (Figure 4A). However, mutation of procaspase-8 DED2 ‘‘FL motif’’ (F122G/L123G; double or single mutant) (Figure 4B) abrogates DED-mediated recruitment of additional procaspase-8 molecules to the DISC, but does not totally prevent procaspase-8 recruitment to FADD (Figure 4C, lanes 2 and 3; Figure S3), suggesting that recruitment to FADD is mediated via the procaspase-8 DED1 pocket. To explore this, we identified and mutated key residues in procaspase-8 DED1 pocket (Y8, R5, L42) that could be involved in interacting with an exposed FL motif on either FADD DED or procaspase-8 DED2 (Figure 4B). Mutation of the Y8 residue significantly reduced both DISC recruitment and activation of procaspase-8 when compared with WT or unprocessed active site mutant (C360A) (Figure 4C, compare lanes 5 and 7 with lanes 1 and 2). Strikingly, recruitment of procaspase-8 DED1 Y8D mutant was substantially less than that of DED2 FL motif mutant (Figure 4C, compare lanes 3 and 5). R5E pocket mutant alone had little effect on procaspase-8 recruitment or activation (Figure 4C, lane 4), while the R5E mutant in combination with Y8D (R5E/Y8D; DM) attenuated procaspase-8 recruitment similar to that observed with Y8D alone. An alternative pocket mutant, L42R, also exhibited reduced procaspase-8 recruitment, though not as great as that observed with the Y8 mutant (Figure 4C, lane 8). Thus, procaspase-8 recruitment to FADD occurs preferentially via procaspase-8 DED1 hydrophobic pocket, and Y8 is a key residue involved in this interaction (Figure 4C, scheme). Additional procaspase-8 molecules are then recruited via the exposed DED2 FL motif of FADD-bound procaspase-8 interacting with the hydrophobic pocket in DED1 of the next procaspase-8 molecule.

The Mechanism of c-FLIP Recruitment to FADD Is Distinct from that of Procaspase-8

Having demonstrated that FADD recruits procaspase-8 via the procaspase-8 DED1 pocket, we next investigated whether c-FLIP is recruited to FADD by the same mechanism. Therefore, we mutated equivalent residues to procaspase-8 in c-FLIP$_S$, namely DED1 pocket residue (H7) and DED2 FL motif (F114/L115) (Figures 5A and S4A), and determined their impact on c-FLIP$_S$ recruitment to the r-DISC. Without procaspase-8, only low levels of WT c-FLIP$_S$ were recruited to FADD (Figure 5B, lane 1). However, mutation of DED2 FL motif (F114G/L115G) abrogated this c-FLIP$_S$ recruitment to the r-DISC, indicating that without procaspase-8, limited binding of c-FLIP to FADD occurs predominantly via c-FLIP DED2 FL motif (Figure 5B, lane 2).

(C) r-DISC was pre-assembled using TRAIL-R1-IcD, r-FADD, and ^{35}S r-Casp-8b C360A (160 μl), and beads were washed to remove unbound protein before re-incubating with increasing amounts of c-FLIP$_S$ (0–150 μl). Beads and supernatants, post first and second bind, were analyzed for r-Casp-8b and c-FLIP$_S$.
(D) TRAIL-R1-IcD pull downs from caspase-8 null Jurkat lysates combined with exogenous r-Casp-8b C360A (0–60 μl) were analyzed for FADD, procaspase-8, and c-FLIP$_{L/S}$ binding and quantified by densitometry. *non-specific band. See also Figure S2.

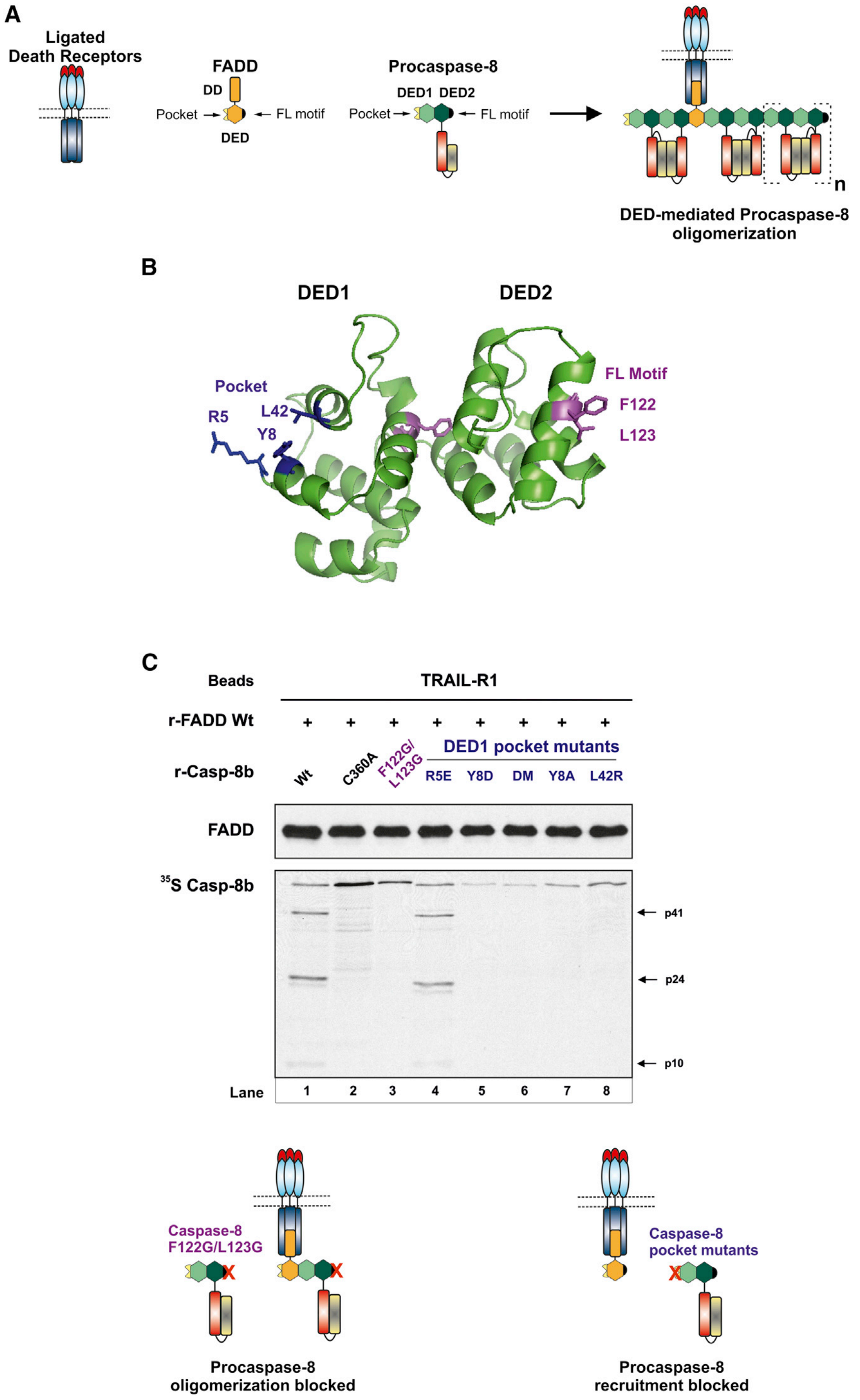

Figure 4. DISC-Bound FADD Recruits Procaspase-8 via Procaspase-8 DED1 Pocket

(A) DISC model in which multiple procaspase-8 molecules are recruited to FADD via DED-mediated interactions.

(B) Modeled structure of procaspase-8 DEDs showing residues in the DED1 pocket that potentially interact with the FL motif of FADD or DED2 of another procaspase-8 molecule.

(C) r-DISC reconstituted using the indicated variants of ^{35}S r-Casp-8b (100 μl) and beads assessed for FADD and r-Casp-8b binding (R5E/Y8D; DM). See also Figure S3. Scheme shows effect of mutating either procaspase-8 DED2 FL motif or key residues in the DED1 pocket.

Mutation of the c-FLIP$_S$ DED1 pocket (H7D) slightly reduced, but did not abolish, c-FLIP$_S$ binding to FADD (Figure 5B, compare lanes 1 and 3). Thus, in the absence of procaspase-8, the limited c-FLIP recruitment to FADD is primarily mediated through c-FLIP DED2 FL motif. Consistent with our earlier observation of co-operative DISC recruitment of c-FLIP and procaspase-8 (Figure 3), procaspase-8 greatly enhanced WT c-FLIP$_S$ binding to the r-DISC (Figure 5B, compare lanes 1 and 4). Notably, in the presence of procaspase-8, c-FLIP$_S$ DED2 F114G/L115G mutant was poorly recruited to the r-DISC with markedly reduced levels compared to WT c-FLIP$_S$ (Figure 5B, lanes 4 and 5). In the presence of procaspase-8, recruitment of low levels of c-FLIP$_S$ F114G/L115G mutant most likely occurs via DED-mediated caspase-8:c-FLIP$_S$ interaction, with mutation of c-FLIP$_S$ DED2 FL motif preventing recruitment of additional DED-containing molecules (Figure 5B). Intriguingly, recruitment of c-FLIP$_S$ DED1 pocket mutant (H7D) was not impaired in the presence of procaspase-8, with binding of c-FLIP$_S$ DED1 pocket mutant comparable to that of WT c-FLIP$_S$ (Figure 5B, compare lanes 4 and 6). Hence, c-FLIP$_S$ DED1 pocket mutant slightly reduced c-FLIP$_S$ recruitment in the absence of procaspase-8, but mutation of c-FLIP$_S$ DED1 pocket did not prevent co-operative recruitment of c-FLIP$_S$ and procaspase-8 to the DISC (Figure S4B). These data provide evidence for a model of c-FLIP recruitment to FADD, which is clearly distinct from that of procaspase-8. Thus, in the absence of caspase-8, limited c-FLIP binding occurs via the c-FLIP DED2 FL motif, whereas procaspase-8 binding to FADD occurs via the procaspase-8 DED1 hydrophobic pocket.

To confirm our model of c-FLIP and procaspase-8 recruitment to the DISC, we tested the effect of a FADD DED hydrophobic pocket mutant (Figure 5A) on r-DISC recruitment of procaspase-8 or c-FLIP$_S$ (minus/plus procaspase-8). When compared with WT FADD, mutation of FADD hydrophobic pocket (H9D) had minimal impact on r-DISC recruitment of procaspase-8 (Figure 5C, lanes 1 and 2). In marked contrast, this FADD DED

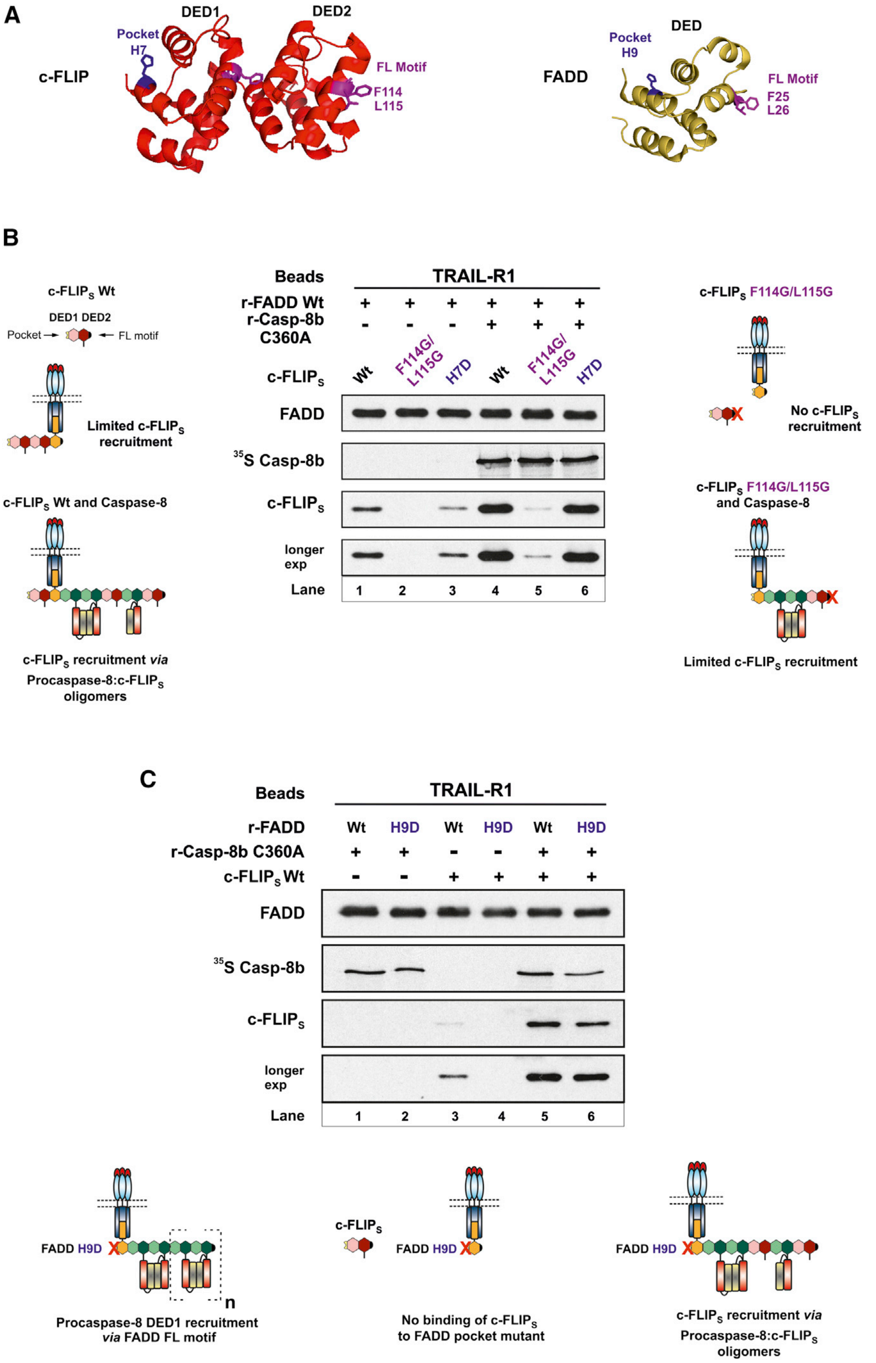

Figure 5. c-FLIP and Procaspase-8 Are Recruited to the DISC via Molecularly Distinct Mechanisms

(A) Modeled structure of c-FLIP and FADD showing DED pocket residues or FL motifs that could potentially interact with another DED molecule.

(B) r-DISC assembled using wild-type (WT), DED2 FL (F114G/L115G), or DED1 pocket (H7D) mutants of c-FLIP$_S$ (50 μl) minus/plus ^{35}S r-Casp-8b C360A (100 μl). Beads were assessed for FADD, r-Casp-8b, and c-FLIP$_S$ binding. Schemes depict c-FLIP recruitment to the DISC in the absence or presence of procaspase-8.

(C) r-DISC reconstituted using WT or DED pocket mutant (H9D) of r-FADD (5 μg) with ^{35}S r-Casp-8b C360A (100 μl) and WT c-FLIP$_S$ (50 μl), singly or in combination, and analyzed as in (B). Scheme showing FADD DED pocket mutant can only bind c-FLIP$_S$ via procaspase-8:c-FLIP$_S$ DED interactions. See also Figure S4.

pocket mutant completely abolished r-DISC recruitment of c-FLIP$_S$ (Figure 5C, lanes 3 and 4), confirming that without procaspase-8, limited c-FLIP$_S$ binding to FADD is mediated via c-FLIP$_S$ DED2 FL motif and the hydrophobic pocket of FADD. Strikingly, when c-FLIP$_S$ recruitment to FADD H9D was assessed in the presence of procaspase-8, r-DISC recruitment of c-FLIP$_S$ was restored (Figure 5C, compare lanes 4 and 6). Moreover, although c-FLIP$_S$ binding to FADD H9D was slightly reduced in the presence of procaspase-8, this correlated with a small reduction in FADD H9D procaspase-8 recruitment (Figure 5C, lanes 5 and 6; Figure S4C). Importantly, similar findings were also obtained with c-FLIP$_L$ (data not shown). Thus, mutation of the H9 residue in the FADD DED pocket blocks c-FLIP$_{L/S}$ recruitment, but addition of procaspase-8 restores c-FLIP$_{L/S}$ recruitment via procaspase-8:c-FLIP DED-mediated interactions (Figure 5C, scheme). Mutation of the FADD pocket only slightly reduced procaspase-8 recruitment, confirming that procaspase-8 binding is primarily mediated through the FL motif of FADD and the DED1 pocket of procaspase-8, whereas c-FLIP is recruited indirectly to the DISC via co-operative binding with procaspase-8 by way of DED-mediated procaspase-8:c-FLIP heterodimerization (Figure 5C, scheme; Figure S4B). Taken together, these data firmly establish a co-operative and hierarchical mechanism for c-FLIP recruitment to the DISC.

c-FLIP$_S$ Inhibits DED-Mediated Oligomerization of Procaspase-8

Previous studies have shown that upon transient overexpression, the DEDs of caspase-8, caspase-10, or FADD can interact to form fibers known as death effector filaments (Shen et al., 2015; Siegel et al., 1998; Vajjhala et al., 2015). To explore the propensity of c-FLIP to form death effector filaments and thus mediate DED:DED interactions, we transiently overexpressed the DEDs of procaspase-8 or c-FLIP$_S$ in HeLa, MCF-7, and HEK293 cells. Intriguingly, death effector filament formation was clearly observed in cells transfected with procaspase-8 DED1-DED2-EGFP, whereas only short filaments/spot-like structures were detected in cells expressing c-FLIP$_S$-EGFP (Figure 6A; Figures

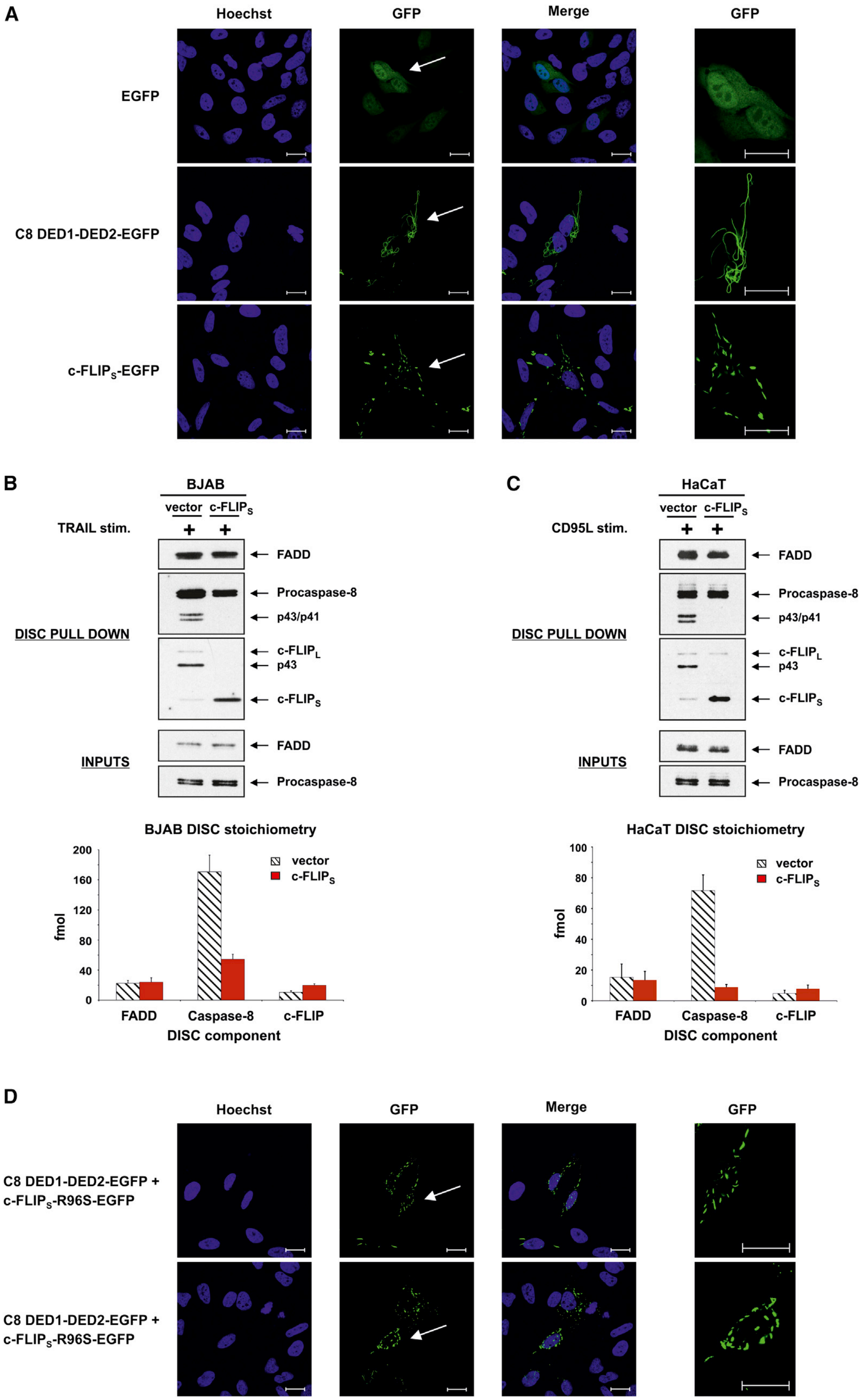

(legend on next page)

S5 and S6). Thus, in contrast to caspase-8 DEDs, c-FLIP$_S$ DEDs do not form death effector filaments. This suggested that, unlike procaspase-8, multiple c-FLIP$_S$ molecules do not preferentially self-associate and consequently may prevent DED-mediated procaspase-8 oligomerization.

To explore this possibility, we examined the effect of c-FLIP$_S$ on procaspase-8 recruitment and native DISC stoichiometry in cells stably expressing c-FLIP$_S$. As reported previously, exposure of the Burkitt's lymphoma cell line, BJAB, to TRAIL (Dickens et al., 2012a), or HaCaT keratinocytes to CD95L (Kavuri et al., 2011) induced receptor-mediated activation of caspase-8, caspase-3, and apoptotic cell death. Treatment of BJAB cells or HaCaT cells stably overexpressing c-FLIP$_S$ with TRAIL or CD95L, respectively, completely blocked caspase cleavage and DR-mediated apoptosis (Figure S7A). Analysis of the native TRAIL or CD95 DISC using ligand affinity purification revealed that FADD recruitment to the DISC did not differ between vector control cells and c-FLIP$_S$ overexpressing cells (Figures 6B and 6C; Figure S7B). In vector control cells, endogenous c-FLIP$_L$ was predominantly detected as a DISC-bound p43 cleavage product, whereas c-FLIP$_S$ was only weakly detected (Figures 6B and 6C). In contrast, c-FLIP$_S$-overexpressing cells exhibited enhanced levels of c-FLIP$_S$ recruitment to the DISC, whereas c-FLIP$_L$ was barely detected. Importantly, in line with our previous report (Kavuri et al., 2011), while both procaspase-8 and p43/41 cleavage fragments were detected in control cells, in c-FLIP$_S$ overexpressing cells DISC recruitment of procaspase-8 was not affected, but caspase-8 cleavage was abolished (Figures 6B and 6C).

The relative stoichiometry of native DISC core components was analyzed in both cell lines by mass spectrometry and label-free absolute quantification, using the "TOP 3" algorithm (Silva et al., 2006). FADD, caspase-8, and c-FLIP were detected by LC-MS/MS analysis of the TRAIL or CD95 DISC from control and FLIP$_S$ overexpressing BJAB (Figure 6B) or HaCaT (Figure 6C) cells, respectively. Label-free absolute quantification revealed that in control cells, both FADD and c-FLIP were sub-stoichiometric compared to caspase-8, with approximately 6-fold more caspase-8 than FADD present within the DISC (Figures 6B and 6C). Importantly, the stoichiometry of FADD:caspase-8 in these cell lines thus agrees with the caspase-8 DED chain model we proposed previously whereby multiple procaspase-8 molecules are recruited to the DISC via a single FADD molecule (Dickens et al., 2012a; Schleich et al., 2012). Strikingly, in the DISC isolated from FLIP$_S$ overexpressing BJAB or HaCaT cells, the stoichiometry of FADD:caspase-8:c-FLIP was markedly reduced to ~1:1:1 (Figures 6B and 6C), with FADD levels remaining similar to those detected in the control DISC. These data show that c-FLIP$_S$ inhibits DISC signaling by limiting the number of procaspase-8 molecules within the DISC, suggesting that c-FLIP$_S$ functions as a terminator of DED-mediated interactions.

To formally test this hypothesis, we transiently overexpressed the DEDs of procaspase-8 and c-FLIP$_S$ in combination, but in the case of c-FLIP$_S$ a non-fluorescent mutated form of EGFP was employed. Consistent with our earlier findings, death effector filament formation was consistently observed in cells transfected with procaspase-8 DED1-DED2-EGFP (Figure 6A; Figures S5 and S6). However, transfection of procaspase-8 DEDs in combination with c-FLIP$_S$ impeded formation of procaspase-8 death effector filaments, demonstrating that c-FLIP$_S$ acts to prevent death effector filament formation (Figure 6D; Figures S5 and S6). Thus, quantitative mass spectrometry as well as co-expression of procaspase-8 DEDs with c-FLIP$_S$ reveal that c-FLIP$_S$ does not readily support DED-mediated interactions. Our data provide direct evidence for an alternative model whereby c-FLIP$_S$ blocks DISC-mediated caspase-8 activation by inhibiting DED-mediated oligomerization of multiple procaspase-8 molecules.

DISCUSSION

c-FLIP is a major regulator of caspase-8, controlling not only DR-mediated apoptosis but also non-apoptotic caspase-8 signaling. The ability of caspase-8 to effect opposing cellular outcomes within key signaling platforms including the DISC, the necrosome, and the ripoptosome depends on the unique ability of c-FLIP isoforms to regulate caspase-8 function. Thus, while c-FLIP$_S$ is widely reported as an inhibitor of caspase-8, c-FLIP$_L$ is more complex as it functions as either an activator or an inhibitor of caspase-8 activation.

So far, the mechanisms underlying the ability of c-FLIP isoforms to differentially regulate caspase-8 have not been elucidated, and current models propose that c-FLIP competes with procaspase-8 for binding to FADD. However, this mechanism has been largely determined from overexpression studies with the assumption that c-FLIP and procaspase-8 bind to FADD in a similar manner (Fricker et al., 2010; Kavuri et al., 2011; Krueger et al., 2001b; Scaffidi et al., 1999). Moreover, the majority of biochemical studies have used either truncated proteins lacking the N-terminal DED domains of c-FLIP$_L$ or caspase-8 (Boatright et al., 2004; Keller et al., 2009) or fusion proteins where the pro-domains are replaced by FKBP/FRB heterodimerization

Figure 6. c-FLIP$_S$ Blocks Cell Death by Inhibiting DED-Mediated Procaspase-8 Oligomerization

(A) HeLa cells were transfected with empty vector (EGFP), GFP-tagged caspase-8 DEDs (C8 DED1-DED2-EGFP), or GFP-tagged c-FLIP$_S$ (c-FLIP$_S$-EGFP) for 24 hr before fixing and staining with Hoechst. Cells were imaged and a representative field for each transfection is shown. Far right panels show enlargement of areas arrowed in GFP panels. Scale bar, 20 μm.

(B) Native TRAIL DISC isolated from 5×10^8 control (vector) and c-FLIP$_S$ expressing BJAB cells stimulated with bTRAIL. Affinity-purified TRAIL DISCs and cleared lysate supernatants (Inputs) were analyzed by western blotting. DISCs were analyzed by label-free quantitative LC-MS/MS to determine the amount of FADD, caspase-8, and c-FLIP (lower panel; mean ± SEM; n = 3).

(C) Native CD95 DISC isolated from 2×10^8 control (vector) and c-FLIP$_S$ expressing HaCaT cells stimulated with Fc-CD95L. DISCs were analyzed and quantified as in (B) (lower panel; mean ± SEM; n = 3).

(D) HeLa cells transfected with GFP-tagged caspase-8 DEDs in combination with c-FLIP$_S$ tagged with a non-fluorescent EGFP mutant (C8 DED1-DED2-EGFP + c-FLIP$_S$-R96S-EGFP) and visualized as in (A). See also Figures S5–S7.

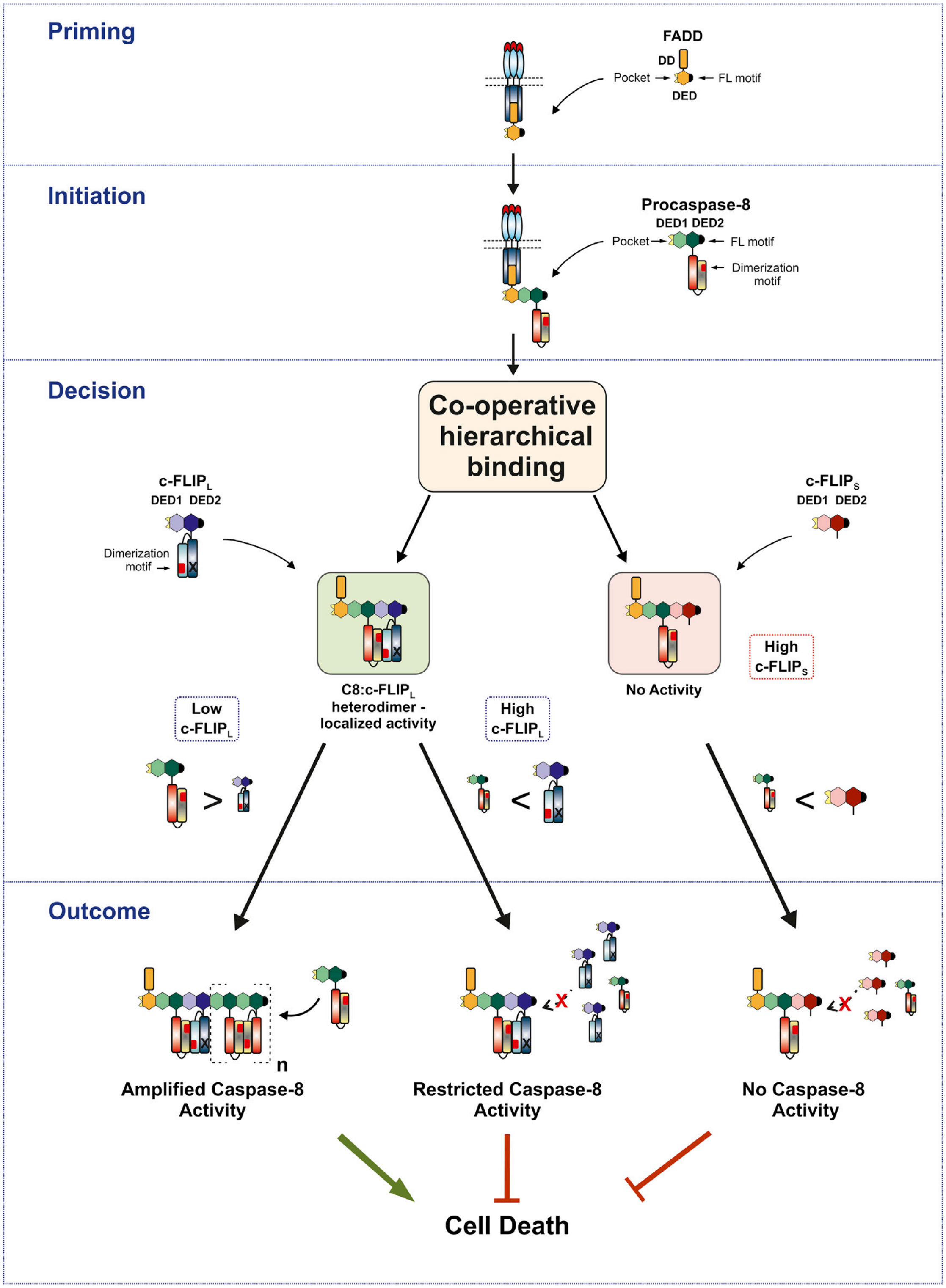

Figure 7. A Unified Model Defines How c-FLIP Isoforms Differentially Regulate Procaspase-8 Activation to Direct Cell Fate

In this model, the priming event is FADD recruitment to ligated DR. Initiation proceeds via procaspase-8 recruitment to FADD, with procaspase-8 DED1 pocket binding to FADD FL motif. Procaspase-8, in turn, recruits and heterodimerizes with c-FLIP$_{L/S}$ via a co-operative and hierarchical binding mechanism. The composition of this procaspase-8:c-FLIP heterodimer then constitutes a key decision step, which determines procaspase-8 activation and subsequent cell fate. Heterodimer composition is critically regulated by the ratio of unbound c-FLIP$_{L/S}$ to procaspase-8; thus, at physiological levels, procaspase-8:c-FLIP$_L$

(legend continued on next page)

domains (Chang et al., 2002; Pop et al., 2011). Importantly, these studies have not addressed how c-FLIP can regulate caspase-8 in the presence of the critical adaptor molecule FADD or within the DISC or other signaling complexes.

To address this, we reconstituted the DISC using full-length recombinant proteins and structure-guided DED mutants. This approach revealed how c-FLIP is recruited to FADD, how different c-FLIP isoforms regulate procaspase-8 activation within its native activation complex, and importantly how this controls cell fate. Our data reveal a fundamental mechanism involving co-operative but hierarchical recruitment of procaspase-8:c-FLIP$_{L/S}$ to FADD where c-FLIP$_S$ inhibits DED-mediated caspase-8 oligomerization. Significantly, our data provide firm evidence that procaspase-8:c-FLIP$_L$ heterodimers initiate procaspase-8 activation within the native DISC and crucially explain the unique ability of c-FLIP$_L$ to promote or inhibit cell death.

Our unique approach using reciprocal binding of individual components in the reconstituted DISC revealed that, contrary to current dogma, c-FLIP does not compete directly with procaspase-8 for binding to FADD (Figure 3). Instead, without caspase-8, c-FLIP binding to FADD is limited, but recruitment to the complex is significantly enhanced when procaspase-8 is present (Figure 3). Crucially, this demonstrates that procaspase-8 binding to FADD is the key initiating event that enables c-FLIP recruitment via c-FLIP heterodimerization with procaspase-8. We validated this procaspase-8:c-FLIP$_{L/S}$ co-operative binding model in a cellular context, using lysates from caspase-8-deficient Jurkat cells, and showed that, without caspase-8, endogenous c-FLIP$_{L/S}$ was not recruited to the DISC (Figure 3). However, addition of exogenous procaspase-8 restored recruitment of both endogenous c-FLIP$_L$ and c-FLIP$_S$ to the complex.

Mutating key residues in procaspase-8 showed that procaspase-8 recruitment to FADD occurs predominantly via procaspase-8 DED1 pocket (Figure 4). In contrast, in the absence of procaspase-8, the limited c-FLIP binding to FADD is mediated via c-FLIP DED2 (Figure 5). Interestingly, mutation of the pocket residue H7 in c-FLIP DED1 did not affect co-operative binding of c-FLIP via procaspase-8. Hence, c-FLIP pocket residue H7, which apparently plays a role in c-FLIP homodimerization, does not participate in procaspase-8-mediated recruitment of c-FLIP to the DISC (Figure S4). This suggests that the procaspase-8 DED2:c-FLIP DED1 interaction is somewhat different from the canonical procaspase-8 DED2:DED1 oligomer interface. Further evidence that c-FLIP recruitment to the complex occurs predominantly via heterodimerization with procaspase-8 is provided by our studies with the FADD DED pocket mutant, which does not recruit c-FLIP unless procaspase-8 is present (Figure 5). This co-operative and hierarchical binding mechanism for FADD recruitment of caspase-8 and c-FLIP clearly challenges existing paradigms (Irmler et al., 1997; Majkut et al., 2014), thus raising the important question of how c-FLIP$_{L/S}$ modulate procaspase-8 activation/activity to produce diverse signaling outcomes.

Our reconstituted DISC experiments fully recapitulated the suggested biphasic effects of c-FLIP$_L$ on procaspase-8 function (Chang et al., 2002; Fricker et al., 2010; Micheau et al., 2002). Thus, c-FLIP$_L$ can activate or inhibit procaspase-8, highlighting that the ratio of c-FLIP$_L$ to procaspase-8 critically determines procaspase-8 signaling. Intriguingly, c-FLIP$_L$-mediated inhibition of caspase-8 was only observed at high concentrations of c-FLIP$_L$ relative to caspase-8, demonstrating that at physiological concentrations (c-FLIP levels are reportedly only 1% of that of caspase-8; Scaffidi et al., 1999) c-FLIP$_L$ preferentially activates procaspase-8 (Figure 2). c-FLIP$_L$-mediated activation of caspase-8 depends on procaspase-8:c-FLIP$_L$ heterodimerization, resulting in localized enzymatic activity in the absence of proteolytic cleavage of either caspase-8 or c-FLIP$_L$ (Figures 1 and 2). These findings are supported by structural studies of truncated c-FLIP$_L$-caspase-8 heterodimer, which show that c-FLIP$_L$ binding to non-cleavable caspase-8 allows rearrangement of the catalytic loops, forming an active site without processing (Boatright et al., 2004; Yu et al., 2009). Thus, loss of c-FLIP$_L$ heterodimerization activity renders c-FLIP$_L$ solely an inhibitor of procaspase-8 (Figure 2; data not shown). Intriguingly, our discovery that c-FLIP recruitment to FADD is indirect and requires procaspase-8 supports a model where FADD recruitment of procaspase-8 initiates preferential recruitment of c-FLIP$_L$ rather than a second procaspase-8 molecule. Thus, in our model, this initiation step enables procaspase-8:c-FLIP$_L$ heterodimers to form the first active protease at the DISC (Figure 7). As levels of c-FLIP$_L$ become depleted, DED-mediated procaspase-8 recruitment then proceeds, facilitating proximity-induced activation and proteolytic cleavage of procaspase-8. Evidence for this mechanism of hierarchical imprinting of DED:DED oligomers, with procaspase-8 activation primed by procaspase-8:c-FLIP$_L$ heterodimers, comes from quantitative mass spectrometry of the native CD95/TRAIL DISC where c-FLIP$_L$ is consistently substoichiometric relative to caspase-8, with approximately 15-fold less c-FLIP$_L$ than caspase-8 detected within the active DISC (Figure 6). Thus, procaspase-8:c-FLIP$_L$ heterodimers comprise only a minor component of the native DISC, and even high cellular levels of c-FLIP$_L$ that inhibit cell death do not shift the stoichiometry in favor of c-FLIP$_L$ (data not shown).

In the reconstituted DISC, c-FLIP$_S$ is a potent inhibitor of caspase-8 as it results in concentration-dependent inhibition of DISC-mediated procaspase-8 activation (Figure 2). Furthermore, native TRAIL and CD95 DISCs isolated from c-FLIP$_S$-overexpressing cells (Figure 6) show that c-FLIP$_S$ completely blocks procaspase-8 processing at the complex, downstream caspase-3 activation, and apoptotic cell death (Figure S7). Our experiments with c-FLIP$_S$ and the reconstituted DISC led us to investigate how c-FLIP$_S$ so potently antagonizes caspase-8 activation (Irmler et al., 1997; Kavuri et al., 2011; Krueger et al., 2001a; Rasper et al., 1998; Scaffidi et al., 1999) yet requires procaspase-8 for initial recruitment to the DISC. Using quantitative mass spectrometry we determined the stoichiometry of c-FLIP$_S$

heterodimer forms the first active protease at the DISC, exhibits localized activity and is an activator, promoting procaspase-8 oligomer assembly and cell death. In contrast, high levels of c-FLIP$_L$ preclude procaspase-8 oligomer assembly, restricting caspase-8:c-FLIP$_L$ heterodimer activity and inhibiting cell death. c-FLIP$_S$ does not readily form DED oligomers; thus, high levels of c-FLIP$_S$ disrupt procaspase-8 oligomer assembly, resulting in a catalytically inactive procaspase-8:c-FLIP$_S$ heterodimer and inhibition of cell death.

and caspase-8 in DISCs isolated from c-FLIP$_S$-overexpressing cells, showing that DR-mediated apoptosis was inhibited. Significantly, the ratio of FADD:caspase-8:c-FLIP in the DISC was now ~1:1:1. This unexpected finding challenges current models (which would predict the stoichiometry to shift in favor of c-FLIP$_S$) and led us to hypothesize that procaspase-8:c-FLIP$_S$ heterodimers are catalytically inactive and moreover inhibit DISC signaling by modulating DED-mediated caspase-8 oligomerization. Our observation that only a finite amount of c-FLIP$_S$ is recruited to a reconstituted DISC (Figure 3) suggested that c-FLIP$_S$ precludes DED oligomer assembly. In support of this, c-FLIP$_S$, like its viral homolog MC159 (Siegel et al., 1998), does not preferentially self-associate to form DED filaments and furthermore blocks caspase-8 DED filament formation (Figure 6; Figures S5 and S6). Taken together, our studies show that c-FLIP$_S$ inhibits caspase-8 activation by disrupting DED-mediated procaspase-8 oligomer assembly, thereby preventing functional alignment of the catalytic dimers ($p18_2/p10_2$) (Figure 7). This alternative mechanism reveals how c-FLIP$_S$ prevents both proximity-induced activation and proteolytic cleavage of caspase-8, thereby abrogating apoptotic cell death. Crucially, procaspase-8:c-FLIP co-operative binding enables c-FLIP$_S$ to block both DISC-mediated oligomerization and activation of procaspase-8, thus explaining why c-FLIP$_S$ is such a potent inhibitor of caspase-8.

Significantly, our procaspase-8:c-FLIP co-operative binding model explains the differential potency of c-FLIP$_L$ and c-FLIP$_S$ to inhibit procaspase-8 activation. Thus, c-FLIP$_S$ potently blocks procaspase-8 processing, but very high levels of c-FLIP$_L$ are required to inhibit caspase-8 (Figure 2). In this case, procaspase-8:c-FLIP$_L$ heterodimers would trigger initial activation and cleavage of procaspase-8 within the complex (Kavuri et al., 2011; Krueger et al., 2001a; Micheau et al., 2002), and as a result high concentrations of c-FLIP$_L$ would be required in order to block subsequent DED-mediated procaspase-8 oligomer assembly. Thus, the balance of c-FLIP$_{L/S}$ to procaspase-8 is critical in determining signaling outcome, and a shift in the stoichiometry of these molecules determines signaling for death or survival (Figure 7).

The co-operative and hierarchical binding model we now propose for c-FLIP regulation of caspase-8 in the context of DR signaling may also be applicable to other FADD:caspase-8: c-FLIP-containing signaling complexes where the ratio of c-FLIP isoforms to procaspase-8 determines cell survival or death. Thus, c-FLIP regulation of caspase-8 is required in T cells, where levels of c-FLIP isoforms modulate cell fate during development of the immune response (Hinshaw-Makepeace et al., 2008; Koenig et al., 2014). Moreover, a shift in the stoichiometry of c-FLIP isoforms and caspase-8 in signaling platforms such as the RIPK1-RIPK3 necrosome, where caspase-8:c-FLIP$_L$ heterodimers suppresses RIPK3-dependent necrosis, would be predicted to have major consequences in vivo. Importantly, our procaspase-8:c-FLIP co-operative binding model provides a long sought after mechanism that uniquely enables c-FLIP$_S$ inhibition of DED-mediated procaspase-8 oligomerization/activation as well as accommodating and explaining the dual functionality of c-FLIP$_L$. This single regulatory mechanism can directly modulate downstream caspase activation and cell fate, thus providing a unified model for c-FLIP regulation of procaspase-8 with signaling outcome critically determined by the ratio of c-FLIP isoforms to procaspase-8.

EXPERIMENTAL PROCEDURES

The Supplemental Experimental Procedures detail expression constructs, antibodies, western blot analysis, apoptosis assays, in vitro GST pull downs, and LC-MS/MS analysis.

DISC Reconstitution

Complete reconstitutions were carried out as described (Hughes et al., 2009), with the following modifications. Beads coated with purified GST-TRAIL-R1/R2-IcD or GST-CD95-IcD (10 μg) (Harper et al., 2003) were incubated with r-FADD (5 μg), and the indicated amounts of IVT-produced procaspase-8b (^{35}S-labeled) plus/minus c-FLIP$_{L/S}$, for 16 hr at 20°C (unless indicated otherwise). Control reconstitutions contained beads coated with GST alone. Bead-associated complexes were analyzed by SDS-PAGE/ western blotting. r-DISC-associated caspase-8 activity was measured by cleavage of the fluorogenic substrate Ac-IETD.AFC or by bioassay using recombinant procaspase-3 active-site mutant (C163A) or Bid (Hughes et al., 2013).

TRAIL and CD95 DISC Analysis

Biotin-labeled TRAIL (bTRAIL) was generated and TRAIL DISC affinity purified from control or c-FLIP$_S$-expressing BJAB cells as described (Dickens et al., 2012a; Hughes et al., 2013). CD95 DISC was affinity purified from control or c-FLIP$_S$-expressing keratinocytes (HaCaT cells) as described (Kavuri et al., 2011), with the following modifications. 5×10^6 HaCaT cells were incubated with 250 U/ml Fc-CD95L for 30 min at 37°C, washed with PBS, and lysed with 2 ml lysis buffer (30 mM Tris-HCl [pH 7.5], 150 mM NaCl, 10% [v/v] glycerol, 1% Triton X-100 [v/v], containing Complete protease inhibitors [Roche Applied Science]) for 60 min at 4°C. DISC complexes were analyzed by western blotting or mass spectrometry.

Quantitative Mass Spectrometry

LC-MS/MS was used to identify and quantify DISC proteins (see Supplemental Experimental Procedures). Briefly, TRAIL and CD95 DISC proteins were separated by SDS-PAGE and serial gel slices digested in situ with trypsin (Dickens et al., 2012a). Extracted tryptic peptides were analyzed using data-independent acquisition (DIA) on a nanoAcquity UPLC system coupled to a Waters Synapt G2-S HDMS mass spectrometer. The PLGS "TOP 3" method was used for absolute quantification of proteins (Silva et al., 2006).

Transfection and Visualization of Death Effector Filaments

HeLa, MCF-7, or HEK293 cells were seeded onto coverslips (coated with gelatin or poly-D-Lysine) 24 hr prior to transfection. Cells were transfected in the presence of the pan-caspase inhibitor, zVAD.FMK (50 μM), and fixed as described (MacFarlane et al., 2000; Dickens et al., 2012a); EGFP alone served as control. Image analysis was performed with Zen 2009 (Zeiss).

Structural Modeling

The modeled structure of the DEDs of caspase-8 (amino acids 1–183; UniProtKB: Q14790) was generated as described (Dickens et al., 2012a). Amino acids 1–177 of c-FLIP (UniProtKB: O15519) were submitted to the Phyre^2 server, and the structure resulting from threading of this sequence through the published structure of MC159 (PDB: 2BBR) was selected as our modeled structure of c-FLIP DED1/2. The DED domains of caspase-8, c-FLIP, and FADD (PDB: 2GF5) were aligned using LSQman. Pymol (www.pymol.org) was used to generate structural models.

SUPPLEMENTAL INFORMATION

Supplemental Information includes Supplemental Experimental Procedures and seven figures and can be found with this article online at http://dx.doi.org/10.1016/j.molcel.2016.02.023.

AUTHOR CONTRIBUTIONS

M.M., M.A.H., and K.C. conceived the study, designed experiments, and analyzed results. M.A.H. carried out the majority of experiments; I.R.P., confocal microscopy; R.J.-J., LC-MS/MS; S.H. and M.F., c-FLIP overexpression; L.F. and J.W.R.S., structural modeling. M.M., M.A.H., M.L., and K.C. wrote the manuscript with input from all co-authors.

ACKNOWLEDGMENTS

This work was supported by the UK Medical Research Council (MRC). M.L. was supported by Deutsche Forschungsgemeinschaft RTG 2099 (Project 9 and 10); Project 10 provided funding for S.H. We dedicate this work to our dear friend, and fellow "Discologist" Prof. Dr. med. Martin Leverkus (1965–2016).

Received: November 2, 2015
Revised: January 22, 2016
Accepted: February 17, 2016
Published: March 17, 2016

REFERENCES

Boatright, K.M., Deis, C., Denault, J.B., Sutherlin, D.P., and Salvesen, G.S. (2004). Activation of caspases-8 and -10 by FLIP(L). Biochem. J. *382*, 651–657.

Chang, D.W., Xing, Z., Pan, Y., Algeciras-Schimnich, A., Barnhart, B.C., Yaish-Ohad, S., Peter, M.E., and Yang, X. (2002). c-FLIP(L) is a dual function regulator for caspase-8 activation and CD95-mediated apoptosis. EMBO J. *21*, 3704–3714.

Dickens, L.S., Boyd, R.S., Jukes-Jones, R., Hughes, M.A., Robinson, G.L., Fairall, L., Schwabe, J.W., Cain, K., and MacFarlane, M. (2012a). A death effector domain chain DISC model reveals a crucial role for caspase-8 chain assembly in mediating apoptotic cell death. Mol. Cell *47*, 291–305.

Dickens, L.S., Powley, I.R., Hughes, M.A., and MacFarlane, M. (2012b). The 'complexities' of life and death: death receptor signalling platforms. Exp. Cell Res. *318*, 1269–1277.

Dillon, C.P., Oberst, A., Weinlich, R., Janke, L.J., Kang, T.B., Ben-Moshe, T., Mak, T.W., Wallach, D., and Green, D.R. (2012). Survival function of the FADD-CASPASE-8-cFLIP(L) complex. Cell Rep. *1*, 401–407.

Esposito, D., Sankar, A., Morgner, N., Robinson, C.V., Rittinger, K., and Driscoll, P.C. (2010). Solution NMR investigation of the CD95/FADD homotypic death domain complex suggests lack of engagement of the CD95 C terminus. Structure *18*, 1378–1390.

Feoktistova, M., Geserick, P., Kellert, B., Dimitrova, D.P., Langlais, C., Hupe, M., Cain, K., MacFarlane, M., Häcker, G., and Leverkus, M. (2011). cIAPs block Ripoptosome formation, a RIP1/caspase-8 containing intracellular cell death complex differentially regulated by cFLIP isoforms. Mol. Cell *43*, 449–463.

Feoktistova, M., Geserick, P., Panayotova-Dimitrova, D., and Leverkus, M. (2012). Pick your poison: the Ripoptosome, a cell death platform regulating apoptosis and necroptosis. Cell Cycle *11*, 460–467.

Fricker, N., Beaudouin, J., Richter, P., Eils, R., Krammer, P.H., and Lavrik, I.N. (2010). Model-based dissection of CD95 signaling dynamics reveals both a pro- and antiapoptotic role of c-FLIPL. J. Cell Biol. *190*, 377–389.

Harper, N., Hughes, M., MacFarlane, M., and Cohen, G.M. (2003). Fas-associated death domain protein and caspase-8 are not recruited to the tumor necrosis factor receptor 1 signaling complex during tumor necrosis factor-induced apoptosis. J. Biol. Chem. *278*, 25534–25541.

Hinshaw-Makepeace, J., Huston, G., Fortner, K.A., Russell, J.Q., Holoch, D., Swain, S., and Budd, R.C. (2008). c-FLIP(S) reduces activation of caspase and NF-kappaB pathways and decreases T cell survival. Eur. J. Immunol. *38*, 54–63.

Hughes, M.A., Harper, N., Butterworth, M., Cain, K., Cohen, G.M., and MacFarlane, M. (2009). Reconstitution of the death-inducing signaling complex reveals a substrate switch that determines CD95-mediated death or survival. Mol. Cell *35*, 265–279.

Hughes, M.A., Langlais, C., Cain, K., and MacFarlane, M. (2013). Isolation, characterisation and reconstitution of cell death signalling complexes. Methods *61*, 98–104.

Irmler, M., Thome, M., Hahne, M., Schneider, P., Hofmann, K., Steiner, V., Bodmer, J.L., Schröter, M., Burns, K., Mattmann, C., et al. (1997). Inhibition of death receptor signals by cellular FLIP. Nature *388*, 190–195.

Kaiser, W.J., Upton, J.W., Long, A.B., Livingston-Rosanoff, D., Daley-Bauer, L.P., Hakem, R., Caspary, T., and Mocarski, E.S. (2011). RIP3 mediates the embryonic lethality of caspase-8-deficient mice. Nature *471*, 368–372.

Kavuri, S.M., Geserick, P., Berg, D., Dimitrova, D.P., Feoktistova, M., Siegmund, D., Gollnick, H., Neumann, M., Wajant, H., and Leverkus, M. (2011). Cellular FLICE-inhibitory protein (cFLIP) isoforms block CD95- and TRAIL death receptor-induced gene induction irrespective of processing of caspase-8 or cFLIP in the death-inducing signaling complex. J. Biol. Chem. *286*, 16631–16646.

Keller, N., Mares, J., Zerbe, O., and Grütter, M.G. (2009). Structural and biochemical studies on procaspase-8: new insights on initiator caspase activation. Structure *17*, 438–448.

Kischkel, F.C., Hellbardt, S., Behrmann, I., Germer, M., Pawlita, M., Krammer, P.H., and Peter, M.E. (1995). Cytotoxicity-dependent APO-1 (Fas/CD95)-associated proteins form a death-inducing signaling complex (DISC) with the receptor. EMBO J. *14*, 5579–5588.

Koenig, A., Buskiewicz, I.A., Fortner, K.A., Russell, J.Q., Asaoka, T., He, Y.W., Hakem, R., Eriksson, J.E., and Budd, R.C. (2014). The c-FLIPL cleavage product p43FLIP promotes activation of extracellular signal-regulated kinase (ERK), nuclear factor κB (NF-κB), and caspase-8 and T cell survival. J. Biol. Chem. *289*, 1183–1191.

Krueger, A., Baumann, S., Krammer, P.H., and Kirchhoff, S. (2001a). FLICE-inhibitory proteins: regulators of death receptor-mediated apoptosis. Mol. Cell. Biol. *21*, 8247–8254.

Krueger, A., Schmitz, I., Baumann, S., Krammer, P.H., and Kirchhoff, S. (2001b). Cellular FLICE-inhibitory protein splice variants inhibit different steps of caspase-8 activation at the CD95 death-inducing signaling complex. J. Biol. Chem. *276*, 20633–20640.

MacFarlane, M., Merrison, W., Dinsdale, D., and Cohen, G.M. (2000). Active caspases and cleaved cytokeratins are sequestered into cytoplasmic inclusions in TRAIL-induced apoptosis. J. Cell Biol. *148*, 1239–1254.

Majkut, J., Sgobba, M., Holohan, C., Crawford, N., Logan, A.E., Kerr, E., Higgins, C.A., Redmond, K.L., Riley, J.S., Stasik, I., et al. (2014). Differential affinity of FLIP and procaspase 8 for FADD's DED binding surfaces regulates DISC assembly. Nat. Commun. *5*, 3350.

Micheau, O., Thome, M., Schneider, P., Holler, N., Tschopp, J., Nicholson, D.W., Briand, C., and Grütter, M.G. (2002). The long form of FLIP is an activator of caspase-8 at the Fas death-inducing signaling complex. J. Biol. Chem. *277*, 45162–45171.

Oberst, A., Pop, C., Tremblay, A.G., Blais, V., Denault, J.B., Salvesen, G.S., and Green, D.R. (2010). Inducible dimerization and inducible cleavage reveal a requirement for both processes in caspase-8 activation. J. Biol. Chem. *285*, 16632–16642.

Oberst, A., Dillon, C.P., Weinlich, R., McCormick, L.L., Fitzgerald, P., Pop, C., Hakem, R., Salvesen, G.S., and Green, D.R. (2011). Catalytic activity of the caspase-8-FLIP(L) complex inhibits RIPK3-dependent necrosis. Nature *471*, 363–367.

Oztürk, S., Schleich, K., and Lavrik, I.N. (2012). Cellular FLICE-like inhibitory proteins (c-FLIPs): fine-tuners of life and death decisions. Exp. Cell Res. *318*, 1324–1331.

Pop, C., Oberst, A., Drag, M., Van Raam, B.J., Riedl, S.J., Green, D.R., and Salvesen, G.S. (2011). FLIP(L) induces caspase 8 activity in the absence of interdomain caspase 8 cleavage and alters substrate specificity. Biochem. J. *433*, 447–457.

Rasper, D.M., Vaillancourt, J.P., Hadano, S., Houtzager, V.M., Seiden, I., Keen, S.L., Tawa, P., Xanthoudakis, S., Nasir, J., Martindale, D., et al. (1998). Cell death attenuation by 'Usurpin', a mammalian DED-caspase homologue that precludes caspase-8 recruitment and activation by the CD-95 (Fas, APO-1) receptor complex. Cell Death Differ. *5*, 271–288.

Salmena, L., Lemmers, B., Hakem, A., Matysiak-Zablocki, E., Murakami, K., Au, P.Y., Berry, D.M., Tamblyn, L., Shehabeldin, A., Migon, E., et al. (2003). Essential role for caspase 8 in T-cell homeostasis and T-cell-mediated immunity. Genes Dev. *17*, 883–895.

Scaffidi, C., Schmitz, I., Krammer, P.H., and Peter, M.E. (1999). The role of c-FLIP in modulation of CD95-induced apoptosis. J. Biol. Chem. *274*, 1541–1548.

Schleich, K., Warnken, U., Fricker, N., Oztürk, S., Richter, P., Kammerer, K., Schnölzer, M., Krammer, P.H., and Lavrik, I.N. (2012). Stoichiometry of the CD95 death-inducing signaling complex: experimental and modeling evidence for a death effector domain chain model. Mol. Cell *47*, 306–319.

Scott, F.L., Stec, B., Pop, C., Dobaczewska, M.K., Lee, J.J., Monosov, E., Robinson, H., Salvesen, G.S., Schwarzenbacher, R., and Riedl, S.J. (2009). The Fas-FADD death domain complex structure unravels signalling by receptor clustering. Nature *457*, 1019–1022.

Shen, C., Yue, H., Pei, J., Guo, X., Wang, T., and Quan, J.M. (2015). Crystal structure of the death effector domains of caspase-8. Biochem. Biophys. Res. Commun. *463*, 297–302.

Shu, H.B., Halpin, D.R., and Goeddel, D.V. (1997). Casper is a FADD- and caspase-related inducer of apoptosis. Immunity *6*, 751–763.

Siegel, R.M., Martin, D.A., Zheng, L., Ng, S.Y., Bertin, J., Cohen, J., and Lenardo, M.J. (1998). Death-effector filaments: novel cytoplasmic structures that recruit caspases and trigger apoptosis. J. Cell Biol. *141*, 1243–1253.

Silva, J.C., Gorenstein, M.V., Li, G.Z., Vissers, J.P.C., and Geromanos, S.J. (2006). Absolute quantification of proteins by LCMSE: a virtue of parallel MS acquisition. Mol. Cell. Proteomics *5*, 144–156.

Tenev, T., Bianchi, K., Darding, M., Broemer, M., Langlais, C., Wallberg, F., Zachariou, A., Lopez, J., MacFarlane, M., Cain, K., and Meier, P. (2011). The Ripoptosome, a signaling platform that assembles in response to genotoxic stress and loss of IAPs. Mol. Cell *43*, 432–448.

Thome, M., and Tschopp, J. (2001). Regulation of lymphocyte proliferation and death by FLIP. Nat. Rev. Immunol. *1*, 50–58.

Vajjhala, P.R., Lu, A., Brown, D.L., Pang, S.W., Sagulenko, V., Sester, D.P., Cridland, S.O., Hill, J.M., Schroder, K., Stow, J.L., et al. (2015). The inflammasome adaptor ASC induces procaspase-8 death effector domain filaments. J. Biol. Chem. *290*, 29217–29230.

Vanlangenakker, N., Vanden Berghe, T., and Vandenabeele, P. (2012). Many stimuli pull the necrotic trigger, an overview. Cell Death Differ. *19*, 75–86.

Varfolomeev, E.E., Schuchmann, M., Luria, V., Chiannilkulchai, N., Beckmann, J.S., Mett, I.L., Rebrikov, D., Brodianski, V.M., Kemper, O.C., Kollet, O., et al. (1998). Targeted disruption of the mouse Caspase 8 gene ablates cell death induction by the TNF receptors, Fas/Apo1, and DR3 and is lethal prenatally. Immunity *9*, 267–276.

Wang, L., Yang, J.K., Kabaleeswaran, V., Rice, A.J., Cruz, A.C., Park, A.Y., Yin, Q., Damko, E., Jang, S.B., Raunser, S., et al. (2010). The Fas-FADD death domain complex structure reveals the basis of DISC assembly and disease mutations. Nat. Struct. Mol. Biol. *17*, 1324–1329.

Yang, J.K., Wang, L., Zheng, L., Wan, F., Ahmed, M., Lenardo, M.J., and Wu, H. (2005). Crystal structure of MC159 reveals molecular mechanism of DISC assembly and FLIP inhibition. Mol. Cell *20*, 939–949.

Yu, J.W., Jeffrey, P.D., and Shi, Y. (2009). Mechanism of procaspase-8 activation by c-FLIPL. Proc. Natl. Acad. Sci. USA *106*, 8169–8174.

Molecular Cell, Volume *61*

Supplemental Information

Co-operative and Hierarchical Binding of c-FLIP and Caspase-8: A Unified Model Defines How c-FLIP Isoforms Differentially Control Cell Fate

Michelle A. Hughes, Ian R. Powley, Rebekah Jukes-Jones, Sebastian Horn, Maria Feoktistova, Louise Fairall, John W.R. Schwabe, Martin Leverkus, Kelvin Cain, and Marion MacFarlane

Supplemental Figures

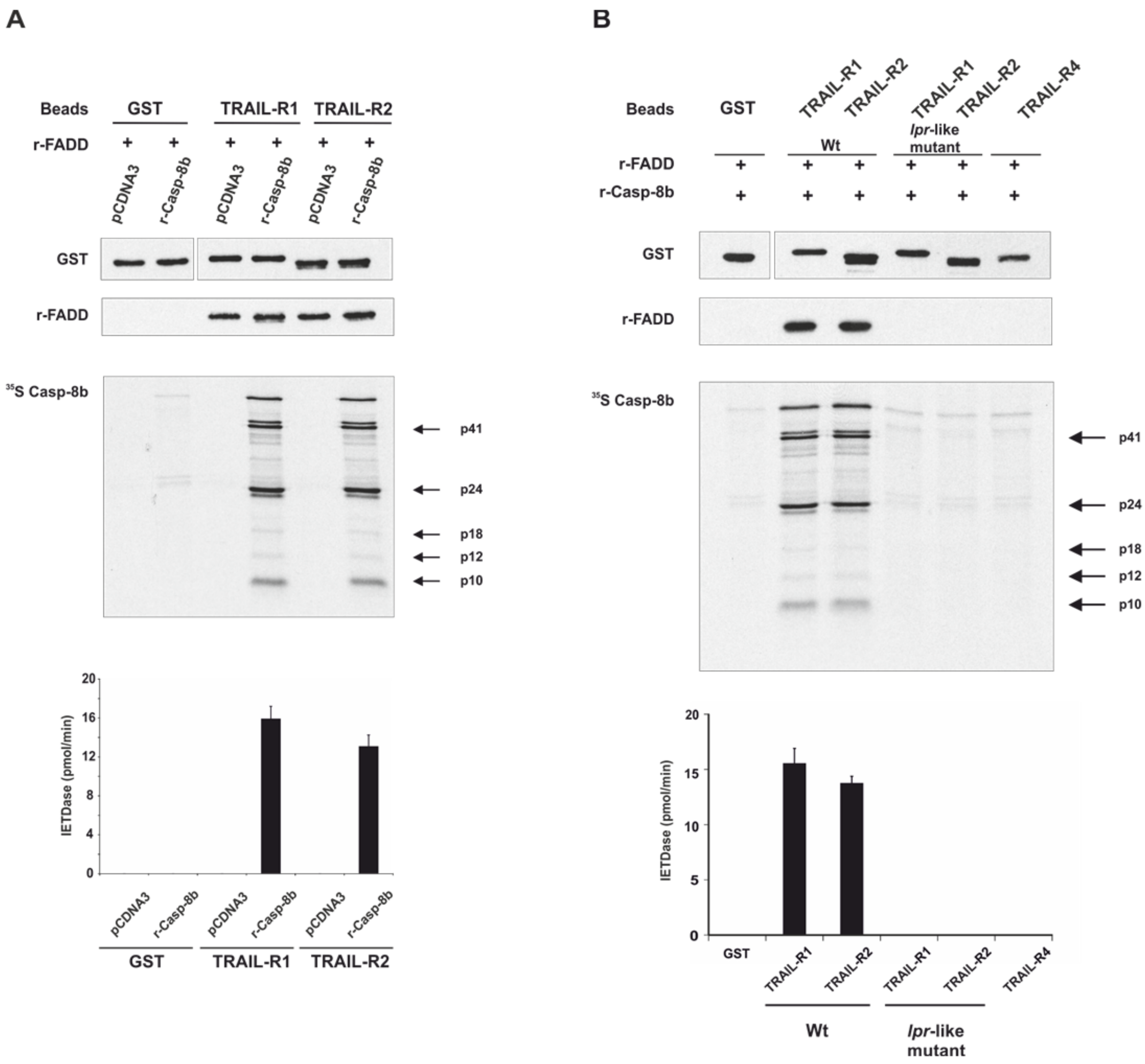

Figure S1, related to Figure 1. Reconstitution of a functional TRAIL-R1/R2 DISC is achieved only in the presence of Wt TRAIL-R1/R2-IcDs

(**A**) A functional DISC (r-DISC) was reconstituted using GST-TRAIL-R1/R2 intracellular domains (TRAIL-R1/R2-IcD) (10 µg), recombinant FADD (r-FADD) (5 µg) and ^{35}S-labeled recombinant procaspase-8b (^{35}S r-Casp-8b; 100 µl) at 20°C for 16 h. ^{35}S-labeled empty vector (pcDNA3) and GST beads were used as controls. Beads were analyzed by SDS-PAGE and autoradiography for ^{35}S r-Casp-8b and immunoblotted for GST and FADD. Beads were also assayed for caspase-8 (IETDase) activity (Mean ± SEM; n=3). (**B**) r-DISCs were assembled using wild-type (Wt) or DD mutant (*lpr*-like) TRAIL-R1/R2-IcDs or TRAIL-R4 (10 µg), r-FADD (5 µg) and ^{35}S r-Casp-8b (100 µl). GST beads were used as control. Beads were analyzed as described in (A). Equal input of GST and GST-TRAIL-R-IcD was confirmed by immunoblotting for GST. *lpr*-like mutations (mature receptor numbering): L366A (TRAIL-R1) and L283A/L312A (TRAIL-R2 short/long isoform).

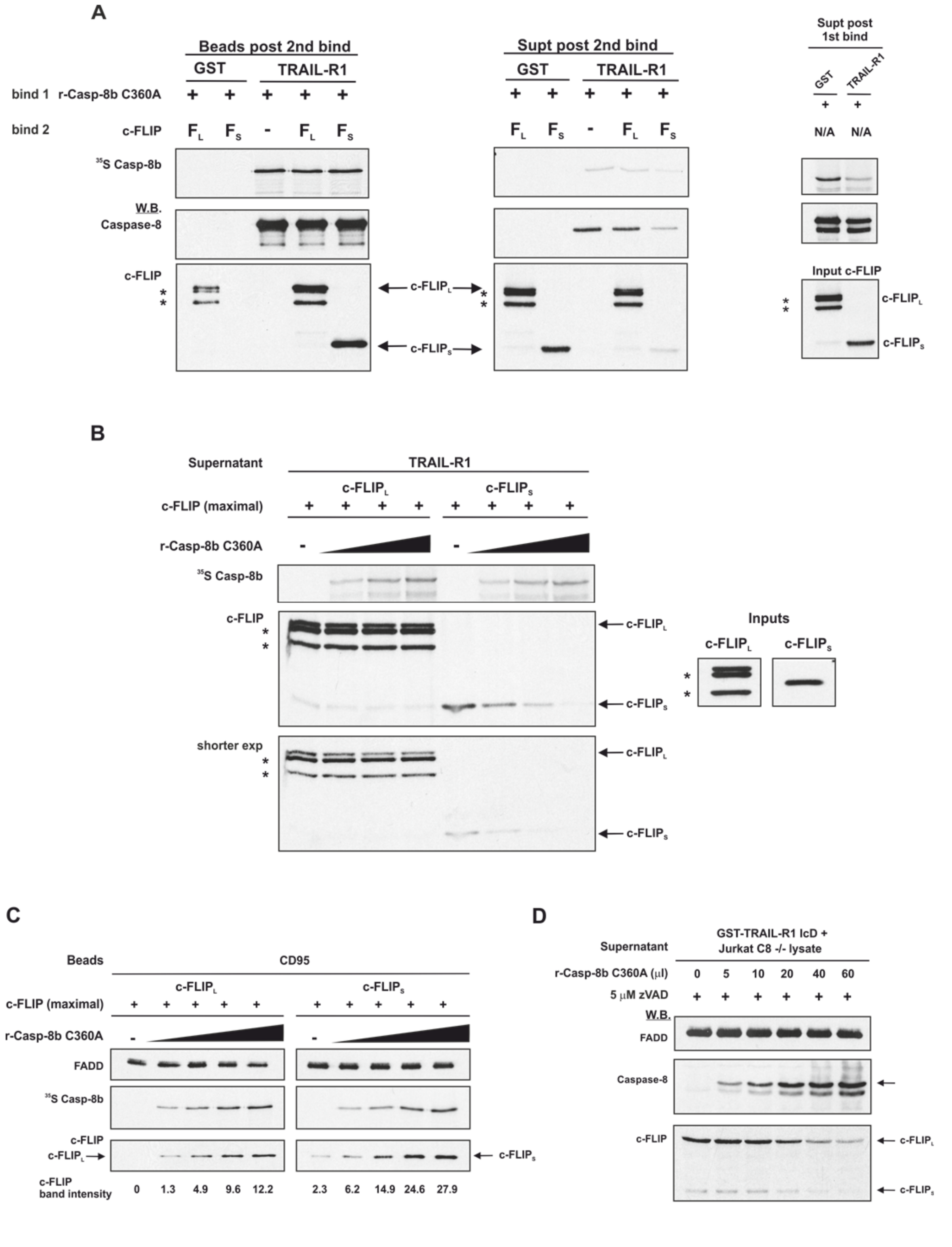

Figure S2, related to Figure 3. DISC reconstitution reveals co-operative and hierarchical recruitment of c-FLIP$_{L/S}$ and procaspase-8

(**A**) c-FLIP$_L$ or c-FLIP$_S$ do not displace procaspase-8 from FADD. The r-DISC was assembled at 16°C for 16 h using TRAIL-R1-IcD, r-FADD and ^{35}S r-Casp-8b C360A (160μl). r-DISC beads were then washed with PBS before incubating with c-FLIP$_{L/S}$ (F_L, F_S; 150 μl) for 16 h at

16°C. Following the second bind, beads and supernatants were assessed for ^{35}S r-Casp-8b and immunoblotted for caspase-8 and c-FLIP. Analysis of the supernatants following r-DISC assembly (supt post 1st bind) is shown in the right hand panel. Equal c-FLIP$_{L/S}$ inputs were confirmed by immunoblotting and GST beads served as control. (**B**) Procaspase-8 enhances c-FLIP$_{L/S}$ recruitment to the r-DISC. The r-DISC was assembled with c-FLIP$_{L/S}$ alone (-) or with increasing amounts of ^{35}S r-Casp-8b C360A. The supernatants (free/unbound proteins) were analyzed for caspase-8 (upper panel) and c-FLIP$_{L/S}$ (lower panels). The corresponding bead-bound r-DISC is shown in Figure 3B. (**C**) The CD95 r-DISC was assembled using GST-CD95-IcD (10 µg), r-FADD (5 µg) and c-FLIP$_{L/S}$ (85 µl) alone (-) or in combination with increasing amounts of ^{35}S r-Casp-8b C360A (0 – 75 µl). Beads were analyzed for FADD (upper panel), caspase-8 (middle panel) and c-FLIP$_{L/S}$ binding (lower panel). *non-specific band. (**D**) TRAIL-R1-IcD pull downs were performed with caspase-8 null Jurkat lysates (10 mg) alone, or in combination with increasing amounts of r-procaspase-8b (r-Casp-8b C360A; 0 – 60 µl) in the presence of zVAD.fmk (5 µM). Supernatants (free/unbound proteins) were analyzed for FADD, procaspase-8 and c-FLIP$_{L/S}$. The corresponding bead-bound complexes are shown in Figure 3D.

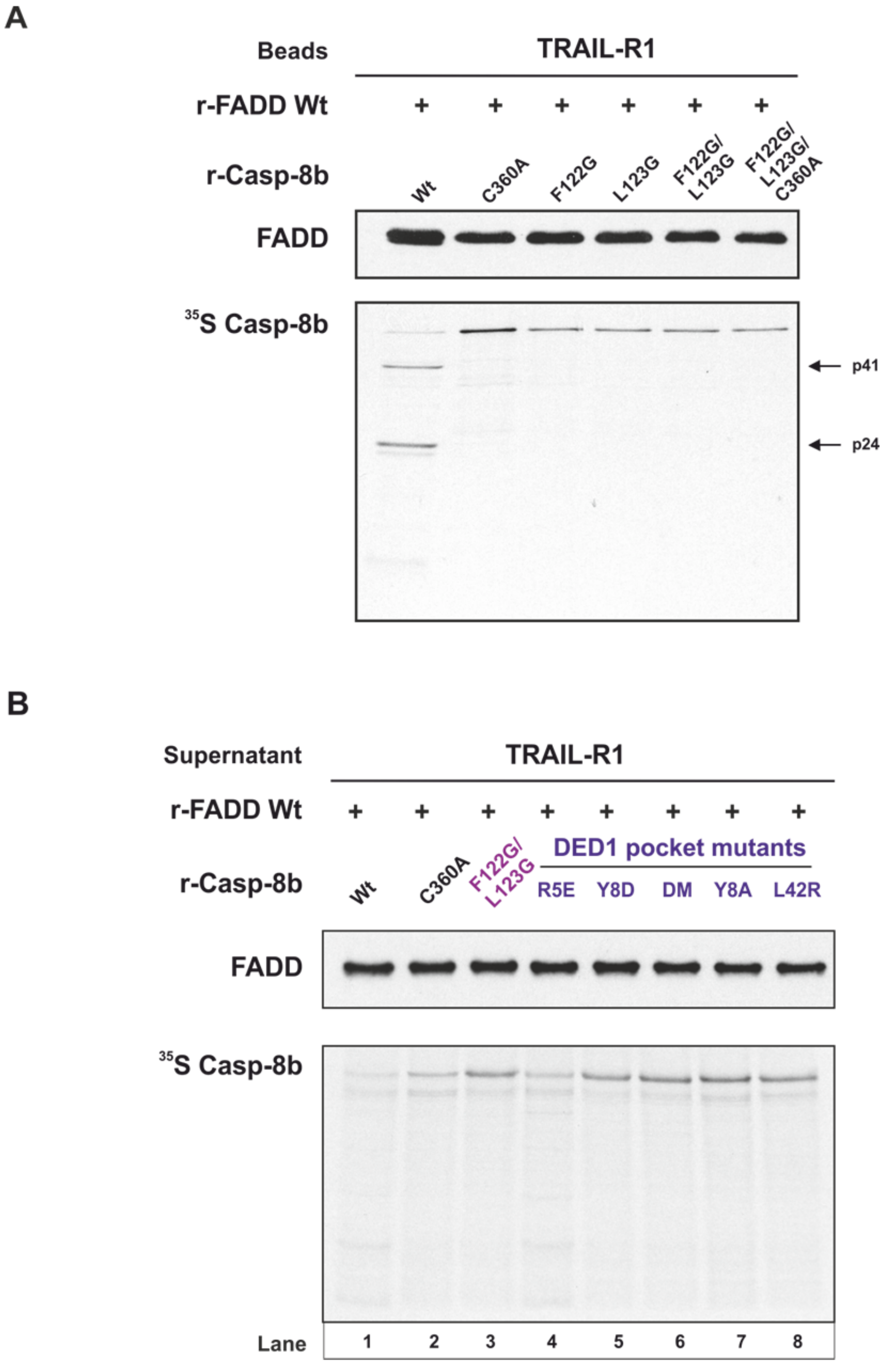

Figure S3, related to Figure 4. Mutation of caspase-8 DED2 F122 or L123 is sufficient to reduce procaspase-8 binding to the r-DISC

(**A**) The r-DISC was assembled using TRAIL-R1-IcD, r-FADD and ^{35}S r-Casp-8b wild-type (Wt), active site mutant (C360A) or DED2 FL motif mutants (F122G; L123G; F122G/L123G; F122G/L123G/C360A) (100 µl). Beads were analyzed for FADD and procaspase-8 binding. (**B**) The r-DISC was reconstituted using the indicated variants of ^{35}S r-Casp-8b (100 µl) and supernatants (free/unbound proteins) assessed for FADD (upper panel) and r-Casp-8b (lower panel). The corresponding bead-bound complexes are shown in Figure 4C.

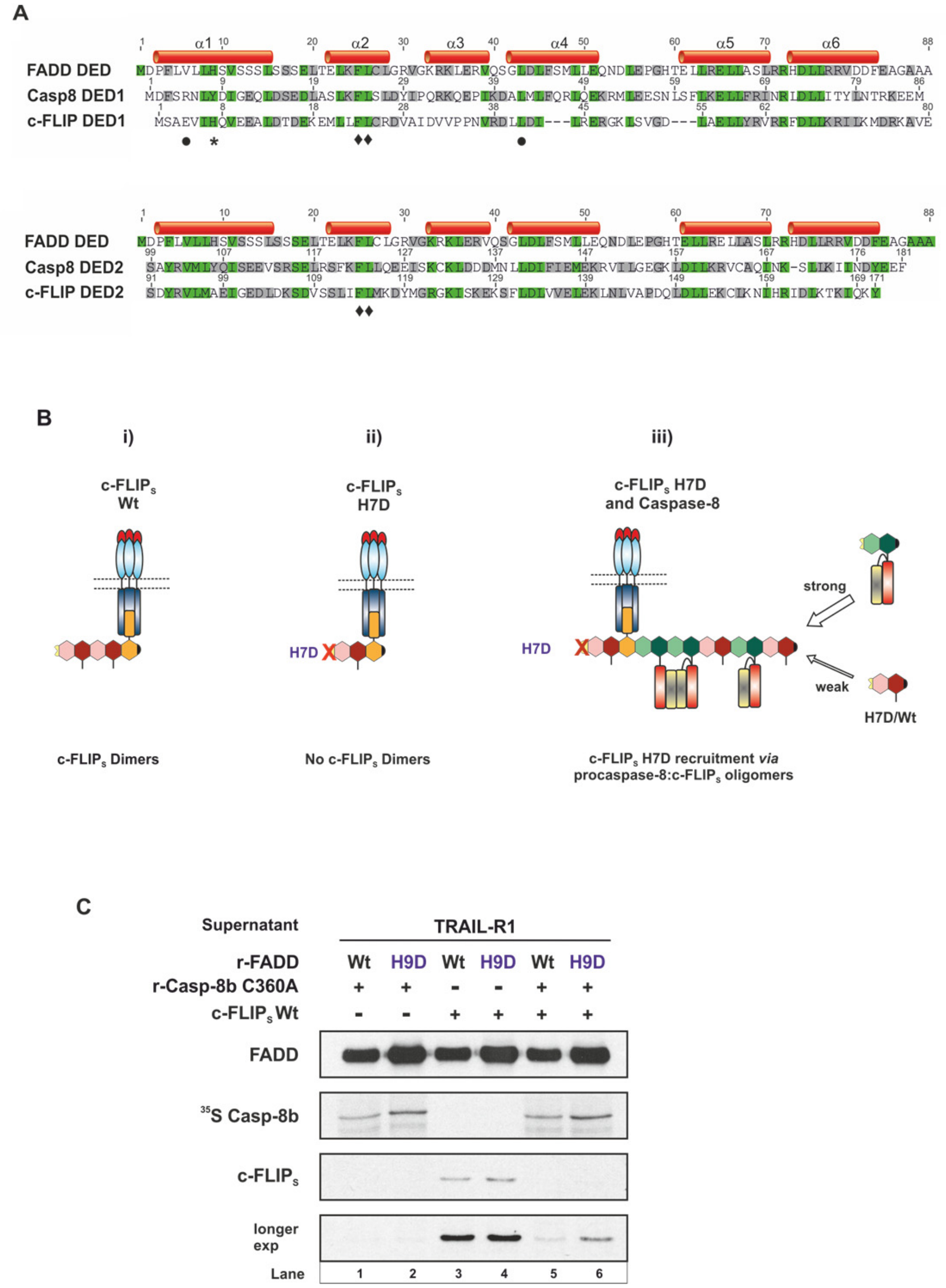

Figure S4, related to Figure 5. c-FLIP and procaspase-8 are recruited to the DISC *via* molecularly distinct mechanisms

(**A**) Sequence alignment of FADD DED with DED1 (upper panel) and DED2 (lower panel) of c-FLIP and caspase-8 performed using CLUSTALW2 (EMBL-EBI). Residues with 100%

similarity are shaded green and 80 - 99% shaded grey. Residues at the conserved hydrophobic patch (FL motif) are marked by ♦. Residues mutated in the DED1 pocket motif of caspase-8, c-FLIP and FADD are indicated by ∗; Pocket residues mutated only in caspase-8 are marked by •. Secondary structures (α-helices) illustrated above the sequences are based on the structure of FADD DED. (**B**) Schematic depicting DISC recruitment of c-FLIP DED1 pocket mutant, in the absence or presence of procaspase-8. In the absence of caspase-8, limited c-FLIP (Wt) recruitment to FADD is mediated *via* the pocket motif of FADD DED and the DED2 FL motif of c-FLIP (i). c-FLIP DED1 pocket mutant (H7D) is still recruited to FADD but is unable to recruit additional c-FLIP molecules (ii). In the presence of procaspase-8, c-FLIP recruitment to the DISC is significantly enhanced and recruitment of c-FLIP DED1 H7D mutant *via* caspase-8:c-FLIP DED interactions is not impaired. c-FLIP does not readily self-associate to form DED oligomers (iii). (**C**) c-FLIP$_S$ is recruited to an r-DISC containing a pocket mutant of FADD, only in the presence of procaspase-8. The r-DISC was assembled using wild-type (Wt) or DED pocket mutant (H9D) of r-FADD (5 µg) with ^{35}S r-Casp-8b C360A (100 µl) and wild-type (Wt) c-FLIP$_S$ (50 µl) either singly or in combination. Supernatants (free/unbound proteins) were assessed for FADD, procaspase-8 and c-FLIP$_S$. The corresponding bead-bound complexes are shown in Figure 5C.

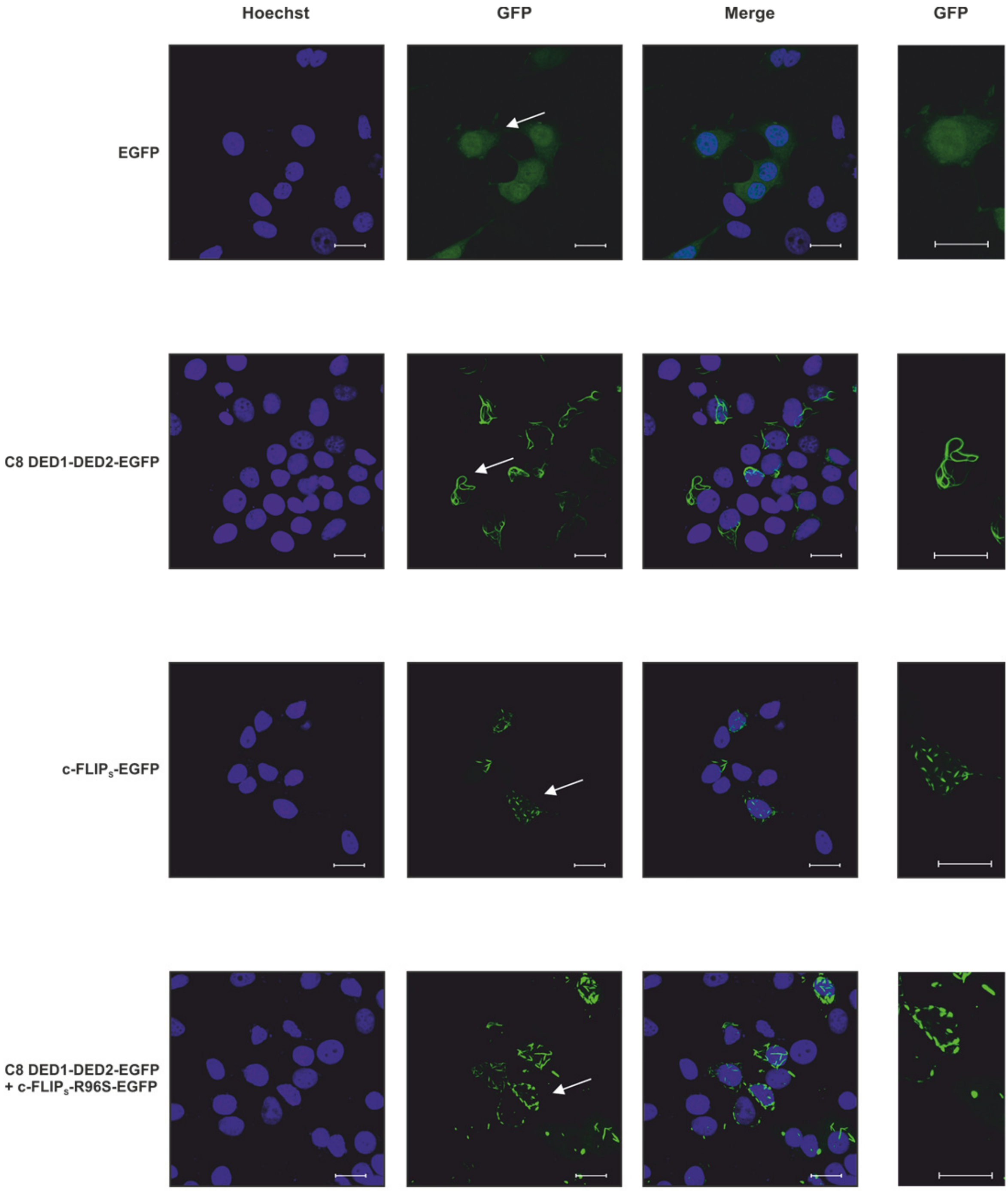

Figure S5, related to Figure 6. c-FLIP$_S$ blocks caspase-8 DED filament formation in MCF-7 cells

MCF-7 cells were transfected with empty vector (EGFP), GFP-tagged caspase-8 DEDs (C8 DED1-DED2-EGFP), GFP tagged c-FLIP$_S$ (c-FLIP$_S$-EGFP) or GFP-tagged caspase-8 DEDs in combination with c-FLIP$_S$ tagged with a non-fluorescent mutant of EGFP (C8 DED1-DED2-EGFP + c-FLIP$_S$-R96S-EGFP) for 24 h before fixing and staining with Hoechst. Cells were imaged using a Zeiss LSM510 confocal microscope, and a representative field for each transfection is shown. Far right panels show enlargement of areas arrowed in GFP panels. Scale bar, 20 μm.

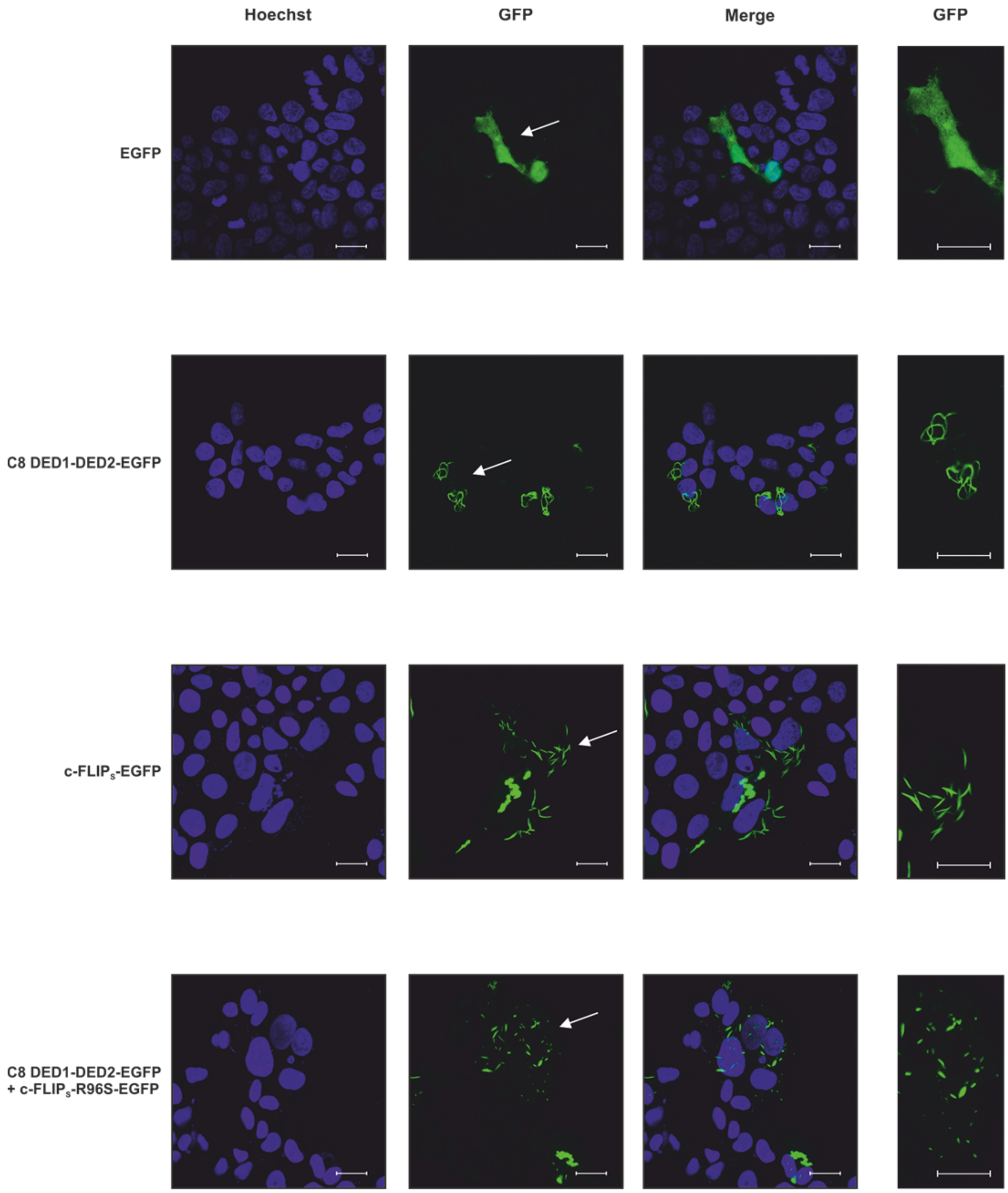

Figure S6, related to Figure 6. c-FLIP$_S$ blocks caspase-8 DED filament formation in HEK293 cells

HEK293 cells were transfected with empty vector (EGFP), GFP-tagged caspase-8 DEDs (C8 DED1-DED2-EGFP), GFP tagged c-FLIP$_S$ (c-FLIP$_S$-EGFP) or GFP-tagged caspase-8 DEDs in combination with c-FLIP$_S$ tagged with a non-fluorescent mutant of EGFP (C8 DED1-DED2-EGFP + c-FLIP$_S$-R96S-EGFP) for 24 h before fixing and staining with Hoechst. Cells were imaged using a Zeiss LSM510 confocal microscope, and a representative field for each transfection is shown. Far right panels show enlargement of areas arrowed in GFP panels. Scale bar, 20 μm.

A

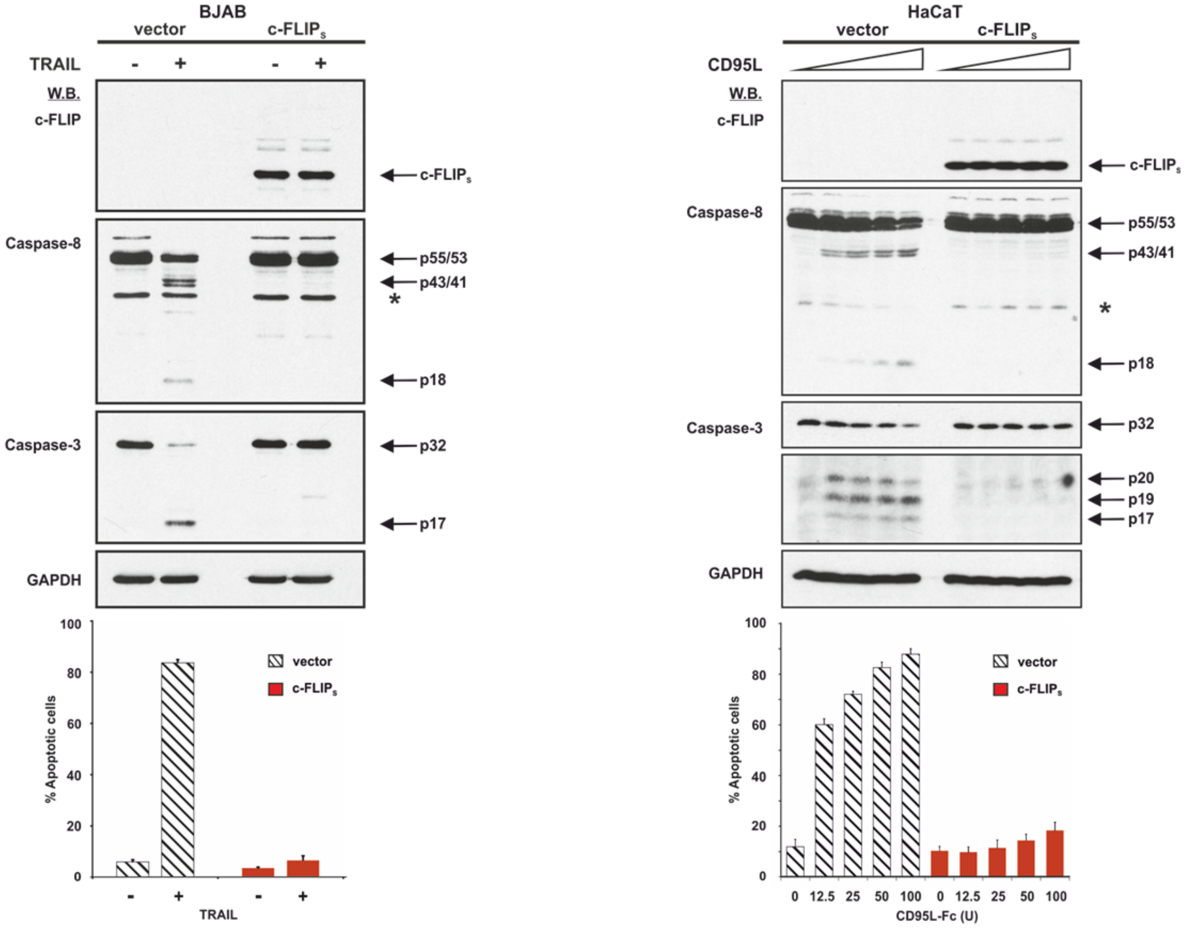
BJAB
vector
c-FLIPs
TRAIL
- + - +
W.B.
c-FLIP
c-FLIPs
Caspase-8
p55/53
p43/41
*
p18
Caspase-3
p32
p17
GAPDH
% Apoptotic cells
100
80
60
40
20
0
vector
c-FLIPs
- + - +
TRAIL
HaCaT
vector
c-FLIPs
CD95L
W.B.
c-FLIP
c-FLIPs
Caspase-8
p55/53
p43/41
*
p18
Caspase-3
p32
p20
p19
p17
GAPDH
% Apoptotic cells
vector
c-FLIPs
0 12.5 25 50 100 0 12.5 25 50 100
CD95L-Fc (U)

B

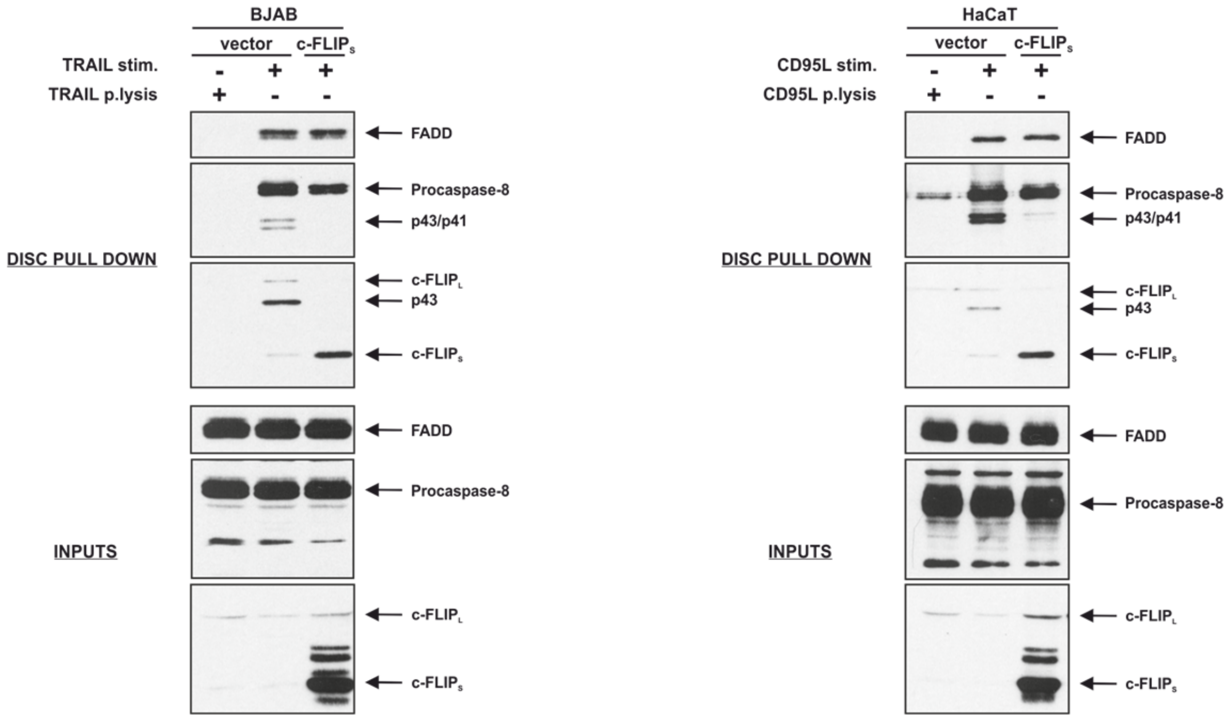
BJAB
vector
c-FLIPs
TRAIL stim.
TRAIL p.lysis
- + +
+ - -
DISC PULL DOWN
FADD
Procaspase-8
p43/p41
c-FLIPL
p43
c-FLIPs
INPUTS
FADD
Procaspase-8
c-FLIPL
c-FLIPs
HaCaT
vector
c-FLIPs
CD95L stim.
CD95L p.lysis
- + +
+ - -
DISC PULL DOWN
FADD
Procaspase-8
p43/p41
c-FLIPL
p43
c-FLIPs
INPUTS
FADD
Procaspase-8
c-FLIPL
c-FLIPs

Figure S7, related to Figure 6. c-FLIP$_S$ blocks CD95 or TRAIL-induced apoptosis by inhibiting DED-mediated procaspase-8 oligomerization.

(**A**) Control (vector) and c-FLIP$_S$ expressing BJAB cells were stimulated for 4 h with 1µg/ml TRAIL (left panel). Control (vector) and c-FLIP$_S$ expressing HaCaT cells were stimulated for 24 h with increasing amounts of CD95L (0 – 100 U/ml) (right panel). Cells were analyzed by immunoblotting for c-FLIP, caspase-8 and caspase-3; GAPDH was included as loading control. Following treatment, apoptotic cell death was assessed by PS externalization (Mean ± SEM; n=3); control cells (hatched bars), c-FLIP$_S$ expressing cells (red bars). *non-specific band. (**B**) Native TRAIL or CD95 DISCs were isolated from control and c-FLIP$_S$ expressing BJAB or HaCaT cells, respectively. DISCs and cleared lysate supernatants (Inputs) were analyzed for FADD, caspase-8, and c-FLIP. Precipitates of non-stimulated cells served as controls (TRAIL p.lysis; CD95L p.lysis).

Supplemental Experimental Procedures

Materials - Media and serum were purchased from Invitrogen (Paisley, UK). Antibodies were sourced as follows: FADD mouse monoclonal antibody (mAb) was from BD Transduction Laboratories; GST tag mouse mAb was from Novagen; Myc mouse mAb antibody, Bid rabbit polyclonal antibody, and cleaved caspase-3 (Asp175) rabbit polyclonal antibody were from Cell Signaling; caspase-8 mouse mAb (C15) and c-FLIP mouse mAb (NF-6) were from Enzo® Life Sciences. c-FLIP$_L$ p43 cleavage fragment was detected with NF-6 mAb and c-FLIP$_L$ p12 cleavage fragment with Myc mAb. Horseradish peroxidase-conjugated secondary antibodies were obtained from Sigma (goat anti-mouse) and DAKO (goat anti-rabbit). HRP-conjugated goat anti-mouse IgG1 and IgG2b were from Southern Biotechnology. The generation of Fc-CD95L was described previously (Geserick et al., 2008). Annexin V-APC was purchased from Life Technologies Ltd. The caspase-8 substrate, Ac-Ile-Glu-Thr-Asp-amino-4-trifluoromethyl coumarin (Ac-IETD.AFC) and the caspase inhibitor z-Val-Ala-Asp(OME)-FMK (zVAD.FMK) were from MP Biomedicals. Recombinant human Bid was from R&D Systems. All other chemicals were of analytical grade and obtained from Sigma-Aldrich or Fisher.

Expression constructs - GST-TRAIL-R1/R2-IcD and CD95-IcD fusion proteins were generated as described previously (Harper et al., 2003). Full length FADD (r-FADD) was generated as described previously (Hughes et al., 2009). Full length procaspase-8b (MACHα2/Mch5b) was cloned in pcDNA3.1 (Invitrogen) and untagged proteins produced by *in vitro* transcription/translation (IVT) (Insect System (Qiagen)), incorporating ^{35}S-methionine (Amersham) (Hughes et al., 2009). Mutations were made using the Stratagene QuikChange Site-Directed Mutagenesis kit and confirmed by DNA sequencing. Proteins from c-FLIP$_{L/S}$-Myc in pcDNA6.1 were produced by IVT (TNT T7-coupled reticulocyte lysate system (Promega)).

Cell culture – Caspase-8-deficient Jurkat T-cells (I9.2) (kind gift from J. Blenis), MCF-7-Fas (MCF-7) cells (kind gift from M. Jattella) or BJAB cell lines stably expressing pCFG5 vector or c-FLIP$_S$ were maintained in RPMI medium, supplemented with 10% fetal bovine serum and 2 mM Glutamax™. HeLa cells, HEK293 cells (both ECACC), or HaCaT cell lines stably expressing pCFG5 vector or c-FLIP$_S$ (Geserick et al., 2009), were maintained in DMEM medium, supplemented with 10% fetal bovine serum. Cells were grown in an atmosphere of 5% CO2 in air at 37°C and maintained in logarithmic growth phase by routine passage every 3-4 days.

Determination of Apoptosis by Annexin V Staining - Apoptotic cells were quantified by measuring externalized phosphatidylserine (PS) assessed by Annexin-V-APC labelling and propidium iodide uptake as described previously (Sun et al., 1999) and were analyzed on a FACSCalibur flow cytometer using CellQuest software (BD Biosciences, San Jose, CA).

Western Blot Analysis - SDS-PAGE and Western immunoblotting were carried out as described previously (MacFarlane et al., 1997).

In vitro GST pull-downs - Jurkat cell lysates were made essentially as described (Harper et al., 2003; Hughes et al., 2013). For receptor pull-downs, 10 mg of cell lysate (20 mg/ml) was incubated with 10 μg of purified GST-TRAIL-R1-IcD fusions bound to glutathione-Sepharose beads for 16 h at 23°C. Where indicated, caspase-8-deficient lysates were supplemented with the specified amounts of IVT generated r-procaspase-8b in the presence of zVAD.fmk (5 μM). Bead-associated complexes were washed four times in PBS containing protease inhibitors, released from beads by boiling for 5 min in SDS sample buffer, and analysed by SDS-PAGE/Western blotting.

Pre-assembly of r-DISC complexes - The r-DISC was initially reconstituted in caspase assay buffer using GST-TRAIL-R1-IcD (10 µg), r-FADD (5 µg) and ^{35}S-labeled r-Casp-8b (160 µl) for 16 h at either 16°C or 20°C. Control reconstitutions contained beads coated with GST alone. The resulting bead complexes were washed four times in PBS (the corresponding supernatant post 1st bind was retained). A second bind was performed by incubating c-FLIP$_{L/S}$ (0 - 150 µl) with the pre-assembled r-DISC in caspase assay buffer for 16 h at either 16°C or 20°C. Bead-associated complexes were washed four times in PBS (the corresponding supernatant post 2nd bind was retained), released from beads by boiling for 5 min in SDS sample buffer, and analysed by SDS-PAGE/Western blotting.

LC-MS/MS identification and quantification of DISC proteins - TRAIL and CD95 DISC proteins were analysed by LC-MS/MS. Briefly, purified protein complexes were separated on SDS-PAGE gels, stained with colloidal coomassie, serially sectioned and the gel slices destained before reduction/alkylation of the proteins and digestion with trypsin (Boyd et al., 2009; Dickens et al., 2012). Extracted tryptic peptides were concentrated to dryness and resuspended in 5% formic acid (FA) and acetonitrile (9:1), spiked with 20-40 fmol/µl ADH1 and BSA MassPREP standards (Waters Corporation, Manchester, UK). Aliquots (2-4 µl) were applied to a reverse phase BEH130 C18 column (25 cm X 75 µm X 1.7 µm I.D.) using a Waters nanoAcquity UPLC system interfaced to a Synapt G2-S HDMS mass

spectrometer. Peptides were eluted (0.3 µl/min) with 50 min, 3-40 % (0.1% FA/acetonitrile) gradients and analysed in data-independent acquisition (DIA) and ion mobility ($HDMS^E$) modes using a T-wave velocity of 650 m/sec (Craxton et al., 2015). Stepped 4 eV and 20-50 eV voltage switching generated collision induced (CID) peptide fragmentation. Low energy and CID LC-MS/MS data were acquired (1 sec cycle scan time and 50-2000 *m/z* mass range) and processed using Waters ProteinLynx Global SERVER (PLGS 3.0) using the UniProt Human database (UniProtKB release 2014_11, 20,265 entries). Peptide mass and fragment mass tolerances were set to auto, with one missed cleavage and variable modifications for methionine oxidation and carbamidomethylation of cysteines. The PLGS "TOP 3" method with a false discovery rate of 1% was used for absolute protein quantification as described previously (Silva et al., 2006a; Silva et al., 2006b).

Supplemental References

Boyd, R.S., Jukes-Jones, R., Walewska, R., Brown, D., Dyer, M.J., and Cain, K. (2009). Protein profiling of plasma membranes defines aberrant signaling pathways in mantle cell lymphoma. Mol Cell Proteomics *8*, 1501-1515.

Craxton, A., Somers, J., Munnur, D., Jukes-Jones, R., Cain, K., and Malewicz, M. (2015). XLS (c9orf142) is a new component of mammalian DNA double-stranded break repair. Cell Death Differ *22*, 890-897.

Dickens, L.S., Boyd, R.S., Jukes-Jones, R., Hughes, M.A., Robinson, G.L., Fairall, L., Schwabe, J.W.R., Cain, K., and MacFarlane, M. (2012). A Death Effector Domain Chain DISC Model Reveals a Crucial Role for Caspase-8 Chain Assembly in Mediating Apoptotic Cell Death. Mol Cell *47*, 291-305.

Geserick, P., Drewniok, C., Hupe, M., Haas, T.L., Diessenbacher, P., Sprick, M.R., Schon, M.P., Henkler, F., Gollnick, H., Walczak, H., *et al.* (2008). Suppression of cFLIP is sufficient to sensitize human melanoma cells to TRAIL- and CD95L-mediated apoptosis. Oncogene *27*, 3211-3220.

Geserick, P., Hupe, M., Moulin, M., Wong, W.W., Feoktistova, M., Kellert, B., Gollnick, H., Silke, J., and Leverkus, M. (2009). Cellular IAPs inhibit a cryptic CD95-induced cell death by limiting RIP1 kinase recruitment. J Cell Biol *187*, 1037-1054.

Harper, N., Hughes, M., MacFarlane, M., and Cohen, G.M. (2003). Fas-associated death domain protein and caspase-8 are not recruited to the tumor necrosis factor receptor 1 signaling complex during tumor necrosis factor-induced apoptosis. J Biol Chem *278*, 25534-25541.

Hughes, M.A., Harper, N., Butterworth, M., Cain, K., Cohen, G.M., and MacFarlane, M. (2009). Reconstitution of the death-inducing signaling complex reveals a substrate switch that determines CD95-mediated death or survival. Mol Cell *35*, 265-279.

Hughes, M.A., Langlais, C., Cain, K., and MacFarlane, M. (2013). Isolation, characterisation and reconstitution of cell death signalling complexes. Methods *61*, 98-104.

MacFarlane, M., Cain, K., Sun, X.M., Alnemri, E.S., and Cohen, G.M. (1997). Processing/activation of at least four interleukin-1beta converting enzyme-like proteases occurs during the execution phase of apoptosis in human monocytic tumor cells. J Cell Biol *137*, 469-479.

Silva, J.C., Denny, R., Dorschel, C., Gorenstein, M.V., Li, G.Z., Richardson, K., Wall, D., and Geromanos, S.J. (2006a). Simultaneous qualitative and quantitative analysis of the Escherichia coli proteome: a sweet tale. Mol Cell Proteomics *5*, 589-607.

Silva, J.C., Gorenstein, M.V., Li, G.Z., Vissers, J.P.C., and Geromanos, S.J. (2006b). Absolute quantification of proteins by LCMSE - A virtue of parallel MS acquisition. Mol Cell Proteomics *5*, 144-156.

Sun, X.M., MacFarlane, M., Zhuang, J., Wolf, B.B., Green, D.R., and Cohen, G.M. (1999). Distinct caspase cascades are initiated in receptor-mediated and chemical-induced apoptosis. J Biol Chem *274*, 5053-5060.
